# A Novel Synthesis Strategy for Poly(Arylene‐Vinylene) Derivatives by Elemental Sulfur‐Mediated Polyolefination

**DOI:** 10.1002/marc.202500166

**Published:** 2025-03-26

**Authors:** Peter Conen, Florian J. O. Niedermaier, Sophia Abou El Mirate, Michael A. R. Meier

**Affiliations:** ^1^ Institute of Organic Chemistry (IOC) Karlsruhe Institute of Technology (KIT) Kaiserstr. 12 76131 Karlsruhe Germany; ^2^ Institute of Biological and Chemical Systems (IBCS‐FMS) Karlsruhe Institute of Technology (KIT) Kaiserstr. 12 76131 Karlsruhe Germany

**Keywords:** aggregation induced emission, conjugated polymers, elemental sulfur, step‐growth polymerization

## Abstract

Herein, a new synthetic access to poly(arylene‐1,2‐diarylvinylene) (PAV‐DA) derivatives is presented by a novel polyolefination of bifunctional *N*‐tosylhydrazones using base‐activated elemental sulfur. PAV‐DAs are particularly interesting due to their aggregation‐induced emission (AIE) properties, resulting in high solid‐state photoluminescence quantum yields. The presented procedure allows the successful synthesis of eight homopolymers and two copolymers with different arylene backbones and aryl side chains from easily accessible monomers, not requiring the use of expensive or highly hazardous materials. The polymers are obtained in good yields and number average molecular weights up to 26.9 kDa. Thermal analysis reveals exceptionally high thermal stability with degradation temperatures as high as 541 °C and glass transition temperatures of up to 263 °C. Furthermore, it is found that the polymer properties are easily adjustable via copolymerization.

## Graphical abstract

A new synthetic access to poly(arylene‐1,2‐diaryl vinylene) derivatives, a class of conjugated polymers with aggregation‐induced emission properties, via an elemental sulfur‐mediated polyolefination of bifunctional *N*‐tosyl hydrazones is presented. The described procedure is more convenient and at least similarly effective to established procedures, as showcased by the synthesis and full characterization of eight homopolymers and two copolymers.

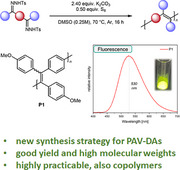

## 1 Introduction

Aggregation‐induced emission (AIE), first described by Tang and coworkers in 2001, refers to the property of an emissive substance to exhibit enhanced luminescence upon increasing aggregation.^[^ ^1^ ^]^ This untypical behavior is in contrast to the aggregation‐caused quenching (ACQ) effect usually observed for conventional luminophores. It renders AIE‐active compounds highly interesting for applications that require luminescent materials to be utilized in their solid state. As such, the field of AIE has received significant attention since its inception, and AIE‐active compounds have seen a plethora of potential uses, for instance as physical and chemical sensors, in bioimaging, or the manufacture of optoelectronic devices.^[^ ^2^, ^3^, ^4^, ^5^, ^6^, ^7^ ^]^ In this context, AIE‐active polymeric materials have similarly been thoroughly researched due to their superior processability and mechanical properties compared to small molecules.^[^ ^8^, ^9^, ^10^, ^11^, ^12^, ^13^ ^]^

It is commonly accepted that AIE properties are the result of both the restriction of intramolecular motion in the aggregated state, as well as the impediment of *π–π*‐stacking due to the typically twisted structure of compounds that show AIE, resulting in the blocking of non‐radiative channels of relaxation. Tetraphenylethylene (TPE), a frequently used template for the design of AIE luminophores, is an archetypal example of an AIE‐active molecule.^[^ ^14^, ^15^, ^16^, ^17^ ^]^

The linear polymeric counterparts of TPEs, poly(arylene‐1,2‐diarylvinylenes) (PAV‐DAs), similarly possess high photoluminescence quantum yields in the solid state. Some PAV‐DAs have further been identified as polymers of intrinsic microporosity, rendering them promising as optical gas sensors based on fluorescence quenching, for instance for the detection of oxygen or nitroaromatic compounds.^[^ ^18^, ^19^, ^20^, ^21^, ^22^ ^]^ Furthermore, PAV‐DAs, as well as other poly(arylene‐vinylene) (PAV) derivatives, have been utilized as luminophores in electro‐optical devices, such as LEDs or solar concentrators.^[^ ^23^, ^24^, ^25^ ^]^ The most prevalent synthesis method for PAV‐DAs is the conversion of diketones into bis(*gem*‐dichlorides), followed by a reductive polyolefination using metallic reducing agents such as chromium(II) salts or cobalt octacarbonyl.^[^ ^19^, ^20^, ^26^, ^27^, ^28^ ^]^ The McMurry coupling of carbonyl compounds with titanium species and a reducing agent, which is the most common method for the synthesis of monomeric TPEs, has only been scarcely used for the synthesis of PAV‐DAs.^[^ ^14^, ^29^, ^30^ ^]^ Other reported procedures are the base‐induced polycondensation of bis(benzhydryl chlorides), the acid‐induced polymerization of bifunctional diazo compounds, or polymerizations based on transition‐metal‐catalyzed cross‐coupling reactions such as the Suzuki–Miyaura reaction.^[^ ^31^, ^32^, ^33^, ^34^, ^35^ ^]^ Most of these methods suffer from severe drawbacks, such as the need for highly toxic or unstable materials, as well as expensive catalysts, significantly compromising their practicability.

**Scheme 1:**
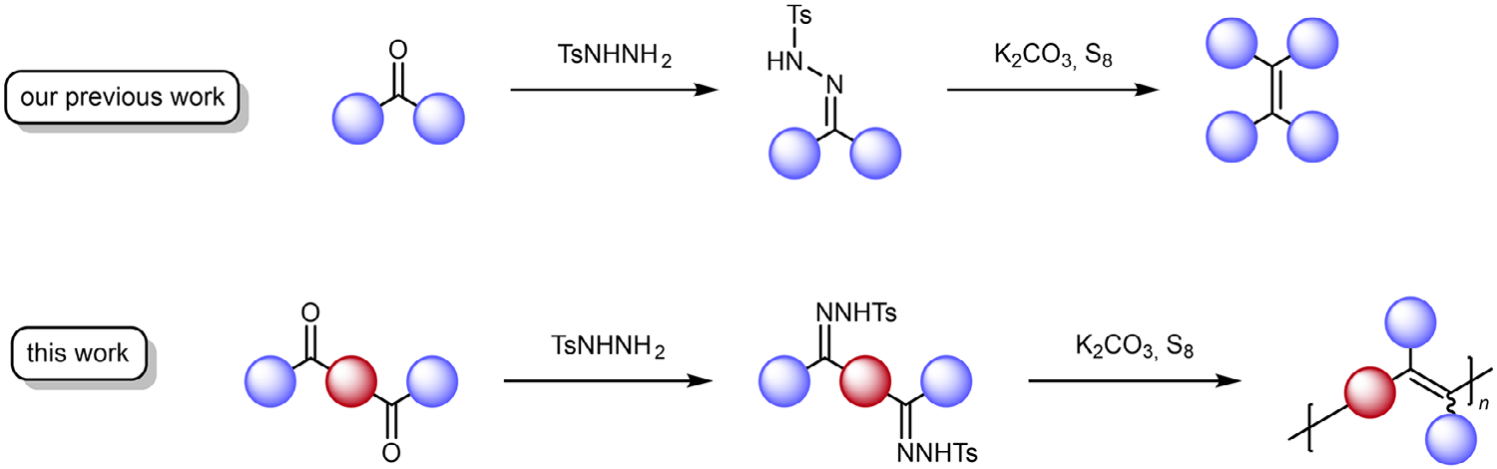
Sulfur‐mediated olefination reaction employed to monofunctional ketones for TPE synthesis (top) and to difunctional ketones for polymerization reactions (bottom).

We have recently reported a synthesis strategy for TPE derivatives via a newly developed homoolefination of benzophenone‐derived *N*‐tosylhydrazones (NTHs) in the presence of base‐activated elemental sulfur.^[^ ^36^, ^37^ ^]^ Herein, we demonstrate the applicability of this method toward bifunctional NTHs, providing novel access to a range of PAV‐DAs (**Scheme** **1**). Bifunctional NTHs are easily accessible from the corresponding diketones, which in turn can be efficiently synthesized via Friedel–Crafts acylation. The presented procedure stands out in comparison to previously reported methods in terms of practicability due to its simple operation, bench‐stable reactants, and lack of expensive and/or highly hazardous materials such as cobalt octacarbonyl, while simultaneously providing similar or superior product yields and molecular weights.

## 2 Results and Discussion

### 2.1 Monomer Synthesis

**Scheme 2:**
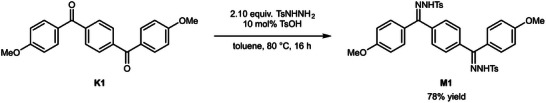
Synthesis of model monomer M1, to be used for polyolefination according to Scheme 1, from the corresponding diketone K1.

First, a methoxy‐substituted substrate model monomer M1 was prepared from diketone K1 (**Scheme** **2**), allowing for easy monitoring of all steps via ^1^H‐NMR spectroscopy due to its methoxy groups. The corresponding diketone K1 was synthesized according to a slightly adapted literature procedure via simple Friedel–Crafts acylation of anisole with commercially available terephthaloyl chloride in the presence of AlCl_3_, obtaining K1 in a yield of 84%.^[^ ^38^ ^]^

For the synthesis of M1 from K1, our previously reported procedure developed for monoketones was initially attempted, using stoichiometric tosyl hydrazide and catalytic toluenesulfonic acid (10 mol%) in refluxing ethanol.^[^ ^36^ ^]^ However, the conversion of K1 remained incomplete, even after extending the reaction time to 72 h and employing a slight excess of tosyl hydrazide. A literature procedure reported by Wang et al. for the synthesis of the corresponding unsubstituted bisNTH (M2, vide infra), employing no catalyst and using methanol as the solvent, unfortunately, left K1 completely unreacted.^[^ ^39^ ^]^ Gratifyingly, it was found that conducting the reaction under our original conditions in toluene instead of ethanol resulted in a complete conversion of K1 after 16 h, yielding monomer M1 in a yield of 78% (Scheme 2). Notably, no formation of monosubstituted NTH, resulting from the condensation of only one of the two carbonyl groups of K1, was observed. Furthermore, ^1^H‐NMR indicated the formation of only one of the three possible stereoisomers of M1, greatly simplifying the workup of the reaction, which could be performed by simple filtration and washing. M1 could hence be synthesized in an overall yield of 66% from cheap and commercially available starting materials in an efficient two‐step process without requiring any elaborate workup methods.

### 2.2 Optimization of Polymerization Conditions

#### 2.2.1 Initial Experiments and Workup Optimization

**Scheme 3:**
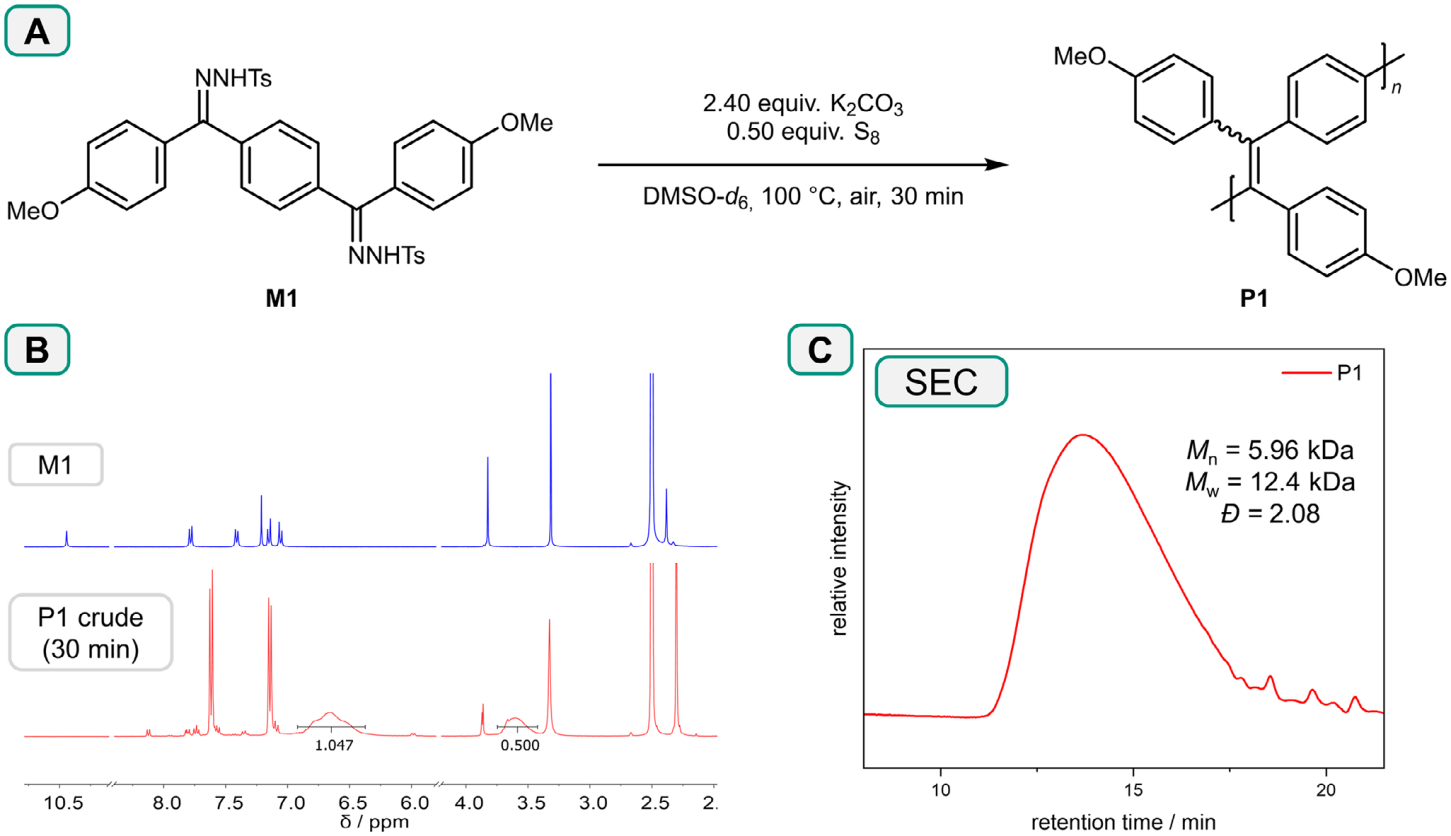
A) Reaction conditions employed for the initial synthesis of P1. B) ^1^H NMR spectra of M1 (top) and the crude reaction mixture of the first polymerization after 30 min (bottom). C) SEC trace of the isolated P1.

Our previously reported conditions for the homocoupling of monofunctional NTHs were employed for the first polymerization attempt.^[^ ^36^ ^]^ Accordingly, M1 was reacted with 2.40 equivalents of potassium carbonate in the presence of 0.50 equivalents of elemental sulfur (corresponding to S_8_) (**Scheme** **3**A). The reaction was conducted at 100 °C under air atmosphere in deuterated dimethyl sulfoxide (DMSO‐*d_6_*) (c(M1) = 0.25 m) and the progress of the polymerization was followed via ^1^H‐NMR spectroscopy. Full conversion of M1 was observed after 30 min, as apparent by the disappearance of its N–H resonance at 10.45 ppm. Furthermore, the emergence of broad signals in both the aromatic and methoxy spectral regions indicated the successful formation of a polymer (Scheme 3B). In order to isolate the product, the reaction mixture was diluted with dichloromethane (DCM), filtered over celite, concentrated in vacuo, and precipitated into cold MeOH, yielding P1 as a dirty yellow powder. Size exclusion chromatography (SEC) measurements (Scheme 3C) indicated a number average molecular weight *M*_n_ of 6 kDa and a dispersity *Ð* of 2.08. However, the obtained yield exceeded 100%, which was attributed to the presence of residual elemental sulfur in the product. This was confirmed via differential scanning calorimetry (DSC), revealing an endothermic peak ≈120 °C, corresponding to the melting temperature of sulfur. Hence, the workup procedure was further optimized before continuing to optimize the polymerization conditions. First, DCM was substituted with chloroform, which was found to be a superior solvent for the polymer. Furthermore, the chloroform phase was subjected to additional washing steps with sodium sulfite solution and water in order to more efficiently remove elemental sulfur. With the optimized workup method, P1 could be isolated in a yield of 46%, now as a bright yellow powder. In this instance, the successful removal of sulfur was confirmed by the absence of a melting peak in the DSC measurement and further underlined by elemental analysis (see Figure S1, Supporting Information). Furthermore, as expected, P1 exhibited yellowish‐green AIE fluorescence under irradiation with ultraviolet light.

After establishing a workup method, further optimizations of the reaction conditions were attempted. Interestingly, it was found that increasing the reaction temperature up to 140 °C led to a decrease in the number average molecular weight of P1, while also exhibiting unsatisfactorily low yields (see Table S1, Supporting Information). It was thus assumed that higher molecular weight polymers can be obtained at lower reaction temperatures. However, in our original report of the reaction using mono‐NTHs, lowering the reaction temperatures below 100 °C was dismissed as not feasible and not further investigated due to incomplete conversion of the starting materials even after prolonged reaction times.^[^ ^36^ ^]^ Hence, further studies were needed in order to examine the applicability of lower reaction temperatures to the olefination reaction, especially since the temperature studies in our previous work were conducted using a different base (i.e., NaH).

#### 2.2.2 Optimization Study of the Monofunctional Homoolefination

**Scheme 4:**
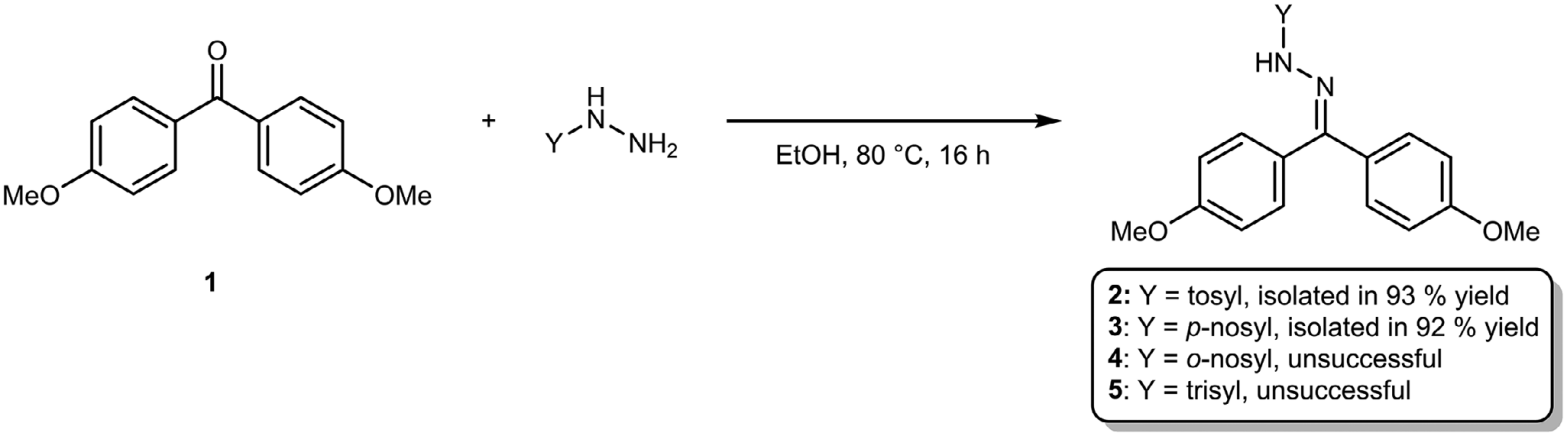
Synthesis attempts of differently substituted hydrazones. Only p‐nosylhydrazone 3 could be successfully isolated.

Further optimization studies using the monofunctional substrate 2, a suitable model for M1, were thus performed. First, monofunctional NTH 2 was synthesized from benzophenone 1 as the new model substance (**Scheme** **4**).^[^ ^36^ ^]^ It was found that the olefination still proceeded well at 90 °C, with a full conversion of 2 reached after 90 min (**Table** **1**, entry 2). At 80 °C, 93% conversion was achieved after 90 min, which increased to 99% after 3 h (Table 1, entry 3). The reaction was simultaneously attempted at 80 °C using vacuum‐dried reagents, flame‐dried glassware, and an argon atmosphere, achieving 99% conversion after 90 min, with full conversion after 3 h (Table 1, entry 4). Generally, fewer unidentifiable signals were observed in the crude NMR spectra of the experiment under an inert atmosphere, indicating fewer side reactions, which is of significant importance to the efficiency of step‐growth polymerizations. Hence, an inert atmosphere was used from this point onward. The reaction could similarly be conducted at 70 °C with 2 being fully converted after 16 h. At 60 °C, we could not achieve complete conversion of 2 even after 72 h (Table 1, entries 5–6).

**Table 1:** Temperature screening reactions using monofunctional sulfonylhydrazones 2 and 3. The reactions were conducted in DMSO‐d_6_ (0.5 m) using 1.20 equiv. K_2_CO_3_ and 0.25 equiv. S_8_. ^1)^ determined by ^1^H‐NMR.

Entry	Hydrazone	Temperature [°C]	Atmosphere	Time [h]	Conversion^1)^
1	2	100	Air	0,5	Full
2	2	90	Air	1.5	Full
3	2	80	Air	1.5	93
				3	99
4	2	80	Argon	1.5	99
				3	Full
5	2	70	Argon	2	64
				4	85
				16	Full
6	2	60	Argon	4	45
				23	94
				72	98
7	3	70	Argon	2	92
				4	96
				16	Full
8	3	60	Argon	4	51
				23	99
				48	Full

Since the rate‐determining step of the olefination reaction is the initial base‐induced decomposition of the NTH into a diazo compound, it was speculated that substitution of the tosyl group of 2 with a better leaving group could potentially allow for the reaction temperature to be lowered even further.^[^ ^36^ ^]^ This can be achieved using more sterically demanding moieties like the triisopropylbenzenesulfonyl (trisyl) moiety or electron‐deficient groups such as nitrobenzenesulfonyl (nosyl) or trifluoromethylbenezenesulfonyl (triftosyl) moieties.^[^ ^40^, ^41^ ^]^ For instance, triftosylhydrazones have been reported to readily decompose at temperatures as low as −40 °C.^[^ ^42^ ^]^

Hence, hydrazides bearing *p*‐nosyl, trisyl, and *o*‐nosyl moieties were synthesized from the commercially available sulfonyl chlorides and hydrazine according to literature procedures, and subsequently reacted with benzophenone 1 in order to generate the corresponding sulfonylhydrazones (Scheme 4).^[^ ^43^ ^]^ *p*‐Nosylhydrazone 3 could be generated in a yield of 92%, albeit an excess of nosyl hydrazide had to be used due to its competing thermal decomposition into the corresponding sulfinic acid. In the other instances, benzophenone 1 was recovered unreacted, suggesting thermal decomposition of the activated hydrazides as the only occurring reaction. Attempting the syntheses at room temperature yielded the same results. It was thus concluded that the mismatch of low electrophilicity of aromatic ketones and the thermal instability of trisyl and *o*‐nosyl hydrazides rendered a synthesis of the corresponding hydrazones 4 and 5 unfeasible. As anticipated, *p*‐nosylhydrazone 3 was indeed found to undergo the olefination reaction at a slightly higher rate than tosylhydrazone 2 (Table 1, entries 7–8). It was possible to achieve a full conversion of 3 at 60 °C within 48 h. Further complementary optimization experiments using different bases and solvents were conducted, however, none were found to be as efficient as the already used potassium carbonate / DMSO system (see **Table** **2**, Supporting Information).

**Table 2:** Optimization experiments for the polymerization of M1. The reactions were conducted in DMSO under inert conditions using 2.40 equiv. K_2_CO_3_ and 0.50 equiv. S_8_. The reaction times were adapted based on the screenings in Table 1.

Entry	Temp.[°C]	Yield	Concentration	*M*_n_ [g mol]^−1^	*M*_w_ [g mol]^−1^	*Ð*
1	100	40%	0.25 m	7800	16 600	2.12
2	120	42%	0.25 m	7800	17 500	2.33
3	90	60%	0.25 m	8800	20 000	2.28
4	80	64%	0.25 m	10 800	24 800	2.3
5	70	76%	0.25 m	16 400	34 200	2.11
6	80	72%	0.5 m	10 700	24 100	2.25
7	80	74%	0.125 m	10 400	21 300	2.05

In order to be able to conduct the polymerization at 60 °C, it was attempted to synthesize the bifunctional *p*‐nosylhydrazone M1’ from K1. Unfortunately, it was observed that the thermal decomposition of *p*‐nosyl hydrazide was more significant in this case. Using 3 equiv. of nosyl hydrazide, only 38% of K1 was converted into M1’ after 8 h, while the remaining hydrazide had fully decomposed (see Section 3.3, Supporting Information). We assume that the low solubility of K1 is responsible for slowing down the hydrazone condensation compared to the thermal decomposition of the hydrazide. Due to these complications, it was opted to not further follow this approach and to instead maintain the more readily accessible tosylhydrazone M1 as the monomer of choice.

#### 2.2.3 Polymerization Under Optimized Conditions

As shown in Table 1, the sulfur‐mediated olefination of monofunctional *N*‐tosylhydrazones could be conducted at 70 °C under inert conditions at the cost of a somewhat longer reaction time but the gain of fewer side reactions. These findings were subsequently applied to the polymerization of M1. The beneficial effect of inert conditions and pre‐dried reagents was apparent when repeating the polymerization at 100 °C, furnishing P1 with an increased number average molecular weight of 7.8 kDa (Table 2, entry 1). Furthermore, as expected, it was found that conducting the polymerization at lower temperatures indeed resulted in a further increase of the molecular weight of P1 (Table 2, entries 2–5), reaching a maximum *M*_n_ of 16.4 kDa at a reaction temperature of 70 °C.

Finally, it was found that variation in the amount of solvent had little to no effect on the efficiency of the polymerization (Table 2, entries 4 and 6–7). Notably, however, increasing the concentration to 0.5 m resulted in a very heterogeneous reaction mixture and stirring difficulties. For this reason, no further optimizations of the stoichiometric ratios of the reactants, i.e., using more sulfur and/or base, were attempted.

### 2.3 Polymerization Scope and Polymer Characterization

After establishing an optimized polymerization method, the synthetic scope of the procedure was investigated. Thus, a range of diketones K2–K8, comprising a structural variety of aryl substituents and arylene backbones, were synthesized similarly to K1 via Friedel–Crafts acylation and obtained in yields between 37 and 83%. Subsequently, the diketones were converted to their respective bis(NTH) monomers M2–M8. In all cases, both the ketone and NTH syntheses could be carried out using the same general protocols used for the synthesis of M1, providing generally good yields and pure products. Merely in the case of M8, slightly lower yields were observed, which we assume is due to the solubilizing effect of the *tert*‐butyl substituents, resulting in increased product losses during washing steps.

**Scheme 5:**
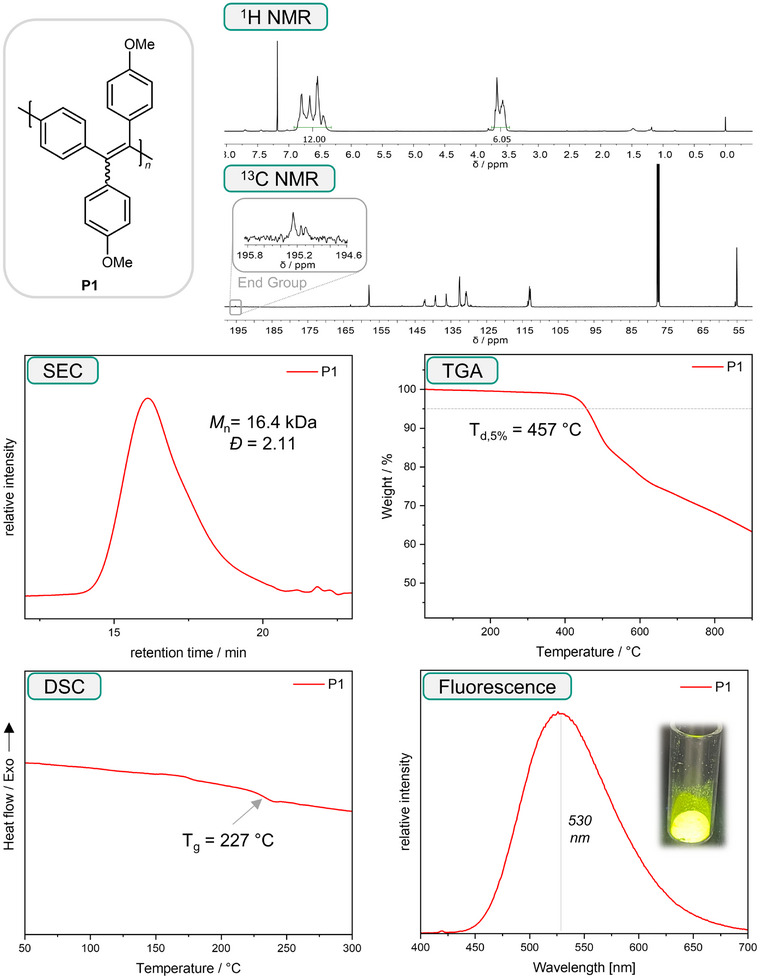
Characterization of polymer P1, including ^1^H and ^13^C NMR, SEC, TGA, DSC, and Fluorescence.

The synthesized bis(NTH)s were subsequently subjected to the optimized polymerization conditions. In all cases, ^1^H‐NMR spectroscopy confirmed the full conversion of M1–M8 after 24 h, and subsequently the products P2–P8 could be isolated using the previously described workup procedure, obtaining yields between 24% and 86% (**Table** **3**). All polymers were fully characterized using ^1^H‐ and ^13^C‐NMR spectroscopy. Exemplarily, the full characterization of P1 from Table 3 is shown in **Scheme** **5**. SEC measurements revealed number average molecular weights between 9.0 and 26.9 kDa and dispersities ≈2, as expected for step‐growth polymerizations. The only exception is P7, which exhibited a very low molecular weight (*M*_n_ = 1.7 kDa), a high dispersity of 4.97, and a low product yield of merely 24%. Contrarily, P2 was obtained with a high number average molecular weight of 26.2 kDa, which significantly outperforms other recently reported methods. For instance, Scherf et al. obtained P2 with an *M*_n_ of 8.3 kDa using a reductive polyolefination with cobalt octacarbonyl (yield 61%).^[^ ^19^, ^44^ ^]^ Older reports for the synthesis of P2 feature even lower molecular weights.^[^ ^30^, ^31^ ^]^ For polymers P3–P8, we have generally achieved similar molecular weights and yields to those in the literature.^[^ ^19^, ^20^, ^30^, ^44^ ^]^ It is however noteworthy that in all cases, except for P7, we have achieved more narrow dispersities ≈2, while for instance for P5 and P8 only highly disperse polymers (*Ð* ≈ 5) have been reported to our knowledge.^[^ ^19^, ^20^ ^]^

**Table 3:** Structural scope of the polyolefination and characterization of the obtained poly(arylene‐vinylene)s P1‐P8. * not observed due to the very low molecular weight obtained.

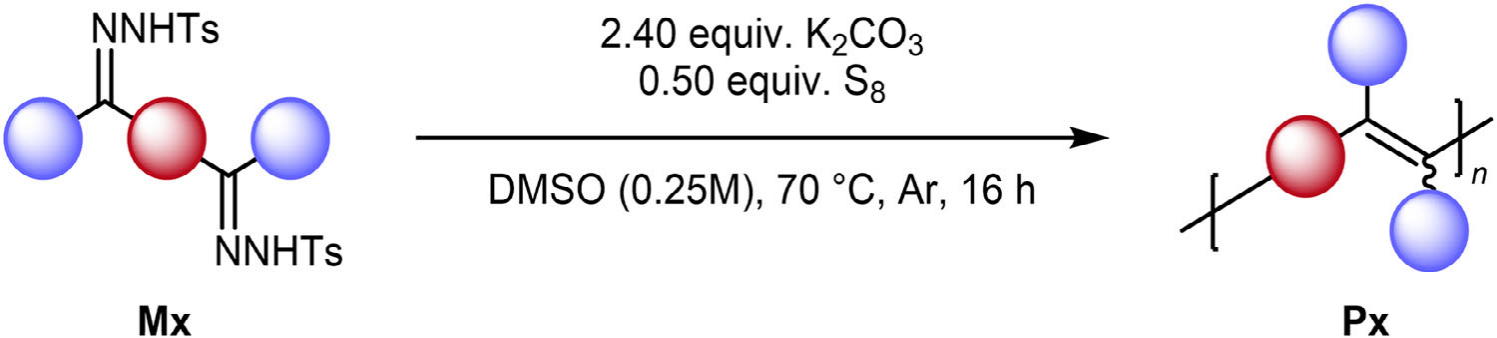							
Polymer	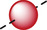	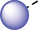	Yield	*M*_n_ [g mol]^−1^	*Ð*	T_d.5%_ /[°C]	T_g_ [°C]
P1	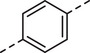	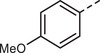	76%	16 200	2.11	457	227
P2	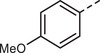	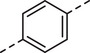	60%	26 900	1.86	541	256
P3	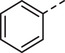	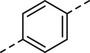	76%	14 700	1.90	516	155
P4	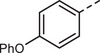	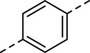	88%	12 500	2.23	471	161
P5	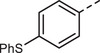	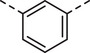	47%	10 300	2.01	538	194
P6	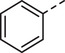	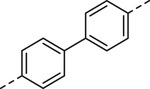	74%	9300	1.61	535	263
P7	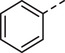	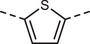	24%	1670	4.97	430	n.o.*
P8	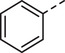	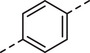	73%	14 900	2.19	514	175

Notably, the polyolefination reaction can result in the formation of both *(E)*‐ and *(Z)‐*configured double bonds. However, it was unfortunately not possible for us to gain any insights into the isomeric ratio in our obtained polymers due to poor resolution in the ^1^H and ^13^C NMR spectra. While there is generally little or no mention of the isomeric ratio of PAV‐DAs synthesized via polyolefination reactions in recent literature reports, a manuscript published by Feast et al. in 1999 claims that PAV‐DA derivatives synthesized via McMurry coupling possess a near‐equal ratio of *(E)*‐ and *(Z)‐*double bonds.^[^ ^19^, ^20^, ^34^ ^]^ We have previously observed that our sulfur‐mediated olefination similarly produces statistical isomeric mixtures for unsymmetrical monofunctional substrates.^[^ ^36^ ^]^ Hence, it might be assumed that the same also applies to the present case.

The thermal properties of all polymers were examined using thermogravimetric analysis (TGA) and DSC (see Scheme 5 for exemplary data for P1, all other data can be found in the, Section 3.2, Supporting Information). TGA measurements showed significant thermal stability of all polymers, as shown by the very high degradation temperatures (T_d,5%_, temperature at which 5% weight loss was detected) in the range between 430 and 538 °C. The obtained results are in good agreement with available data for literature‐known polymers (P2–P5). The high thermal stability most likely stems from the conjugated hydrocarbon character of the polymers. DSC measurements revealed very high glass transition temperatures for the synthesized polymers, ranging from 155 °C for P3 to 263 °C for P6. It was generally observed that the presence of additional substituents on the phenyl side chains lowered the T_g_ (for instance P3, P4, P8), while the polymers bearing unsubstituted phenyl side chains exhibit the highest glass transition temperatures (P2, P6). For P7, we could not detect a glass transition temperature, possibly due to its low degree of polymerization. Furthermore, DSC measurements confirmed the successful removal of elemental sulfur from all polymers due to the absence of an endothermic melting peak ≈120 °C.

As anticipated, most of the synthesized polymers exhibited characteristic AIE fluorescence under UV irradiation, being non‐emissive in solution but highly emissive in aggregated states (compare Scheme 5 for P1). Most polymers were obtained as yellow solids and exhibited yellowish‐green fluorescence (measured as nanoprecipitate in ethanol/chloroform 9:1) with emission maxima between 513 and 531 nm. The exceptions were P5 and P7, the former being an off‐white solid with a blue fluorescence at 473 nm, and the latter being a dark red solid and non‐emissive both in solution and in the solid state.

In order to determine the nature of the polymer end groups, a ^13^C‐NMR study (see Scheme 5) of a sample of P1 sample revealed a low‐intensity resonance at 195 ppm, which is indicative of a carbonyl function (the carbonyl resonance of K1 is at 194 ppm). This is easily explained considering the polymerization mechanism. The reaction proceeds via a diazo intermediate, which subsequently reacts to a thioketone with elemental sulfur, as recently explained in the literature.^[^ ^36^, ^37^ ^]^ The thioketone subsequently reacts with a prior intermediate of the reaction mechanism to form a thiirane, representing the addition step of the polymerization. Nearing complete conversion of the NTH, we expect that once no more diazo compound is being generated, a small amount of thioketone that cannot react further remains as the polymer end group. Subsequently, due to the aqueous workup conditions, the thiocarbonyl functions are hydrolyzed to carbonyl functions, which were observed in ^13^C NMR.

**Scheme 6:**
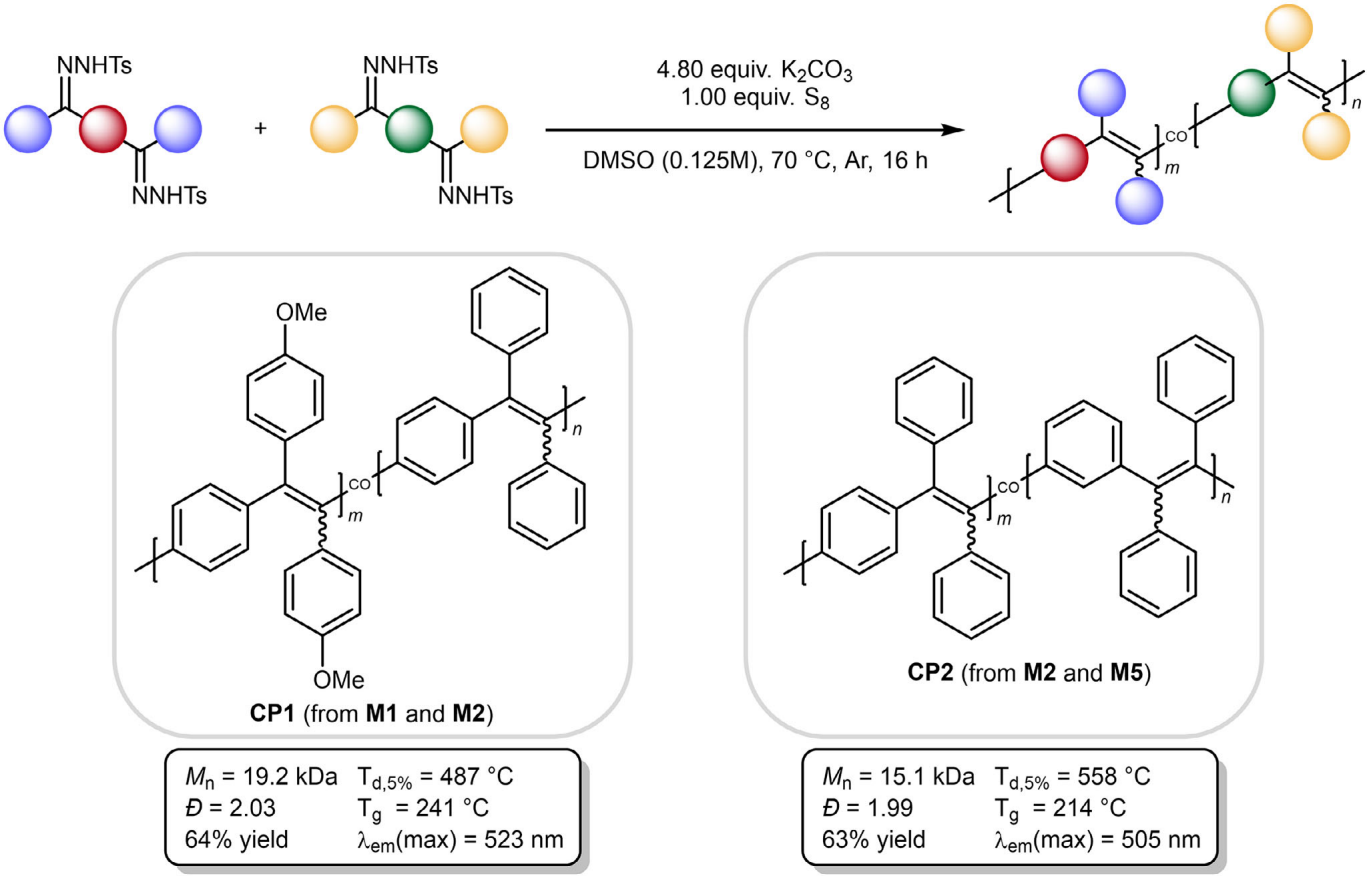
Synthesis and characterization of copolymers CP1 and CP2.

Next, the synthesis of copolymers was attempted by mixing monomers M1 and M2 (CP1) as well as M1 and M5 (CP2), respectively in equal ratios (**Scheme** **6**). Both copolymers could be isolated as yellow solids, underlining the versatility of polymer design using the newly established synthetic procedure.

**Figure 1:**
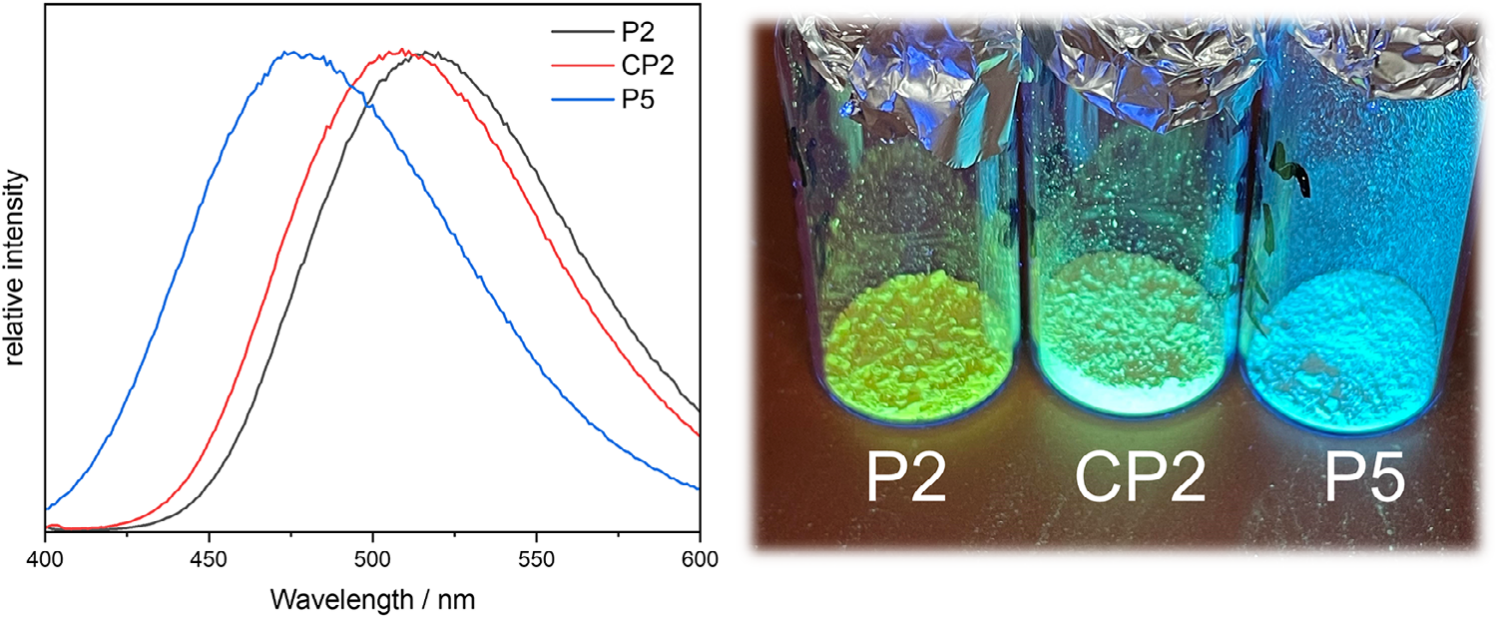
Comparison of the fluorescence spectra of copolymer CP2 and the respective homopolymers P2 and P5.

The structure of CP1 could be confirmed via the ratio of aromatic and methoxy protons in the ^1^H NMR spectrum, CP2 showed overlaying peaks in the aromatic region due to the chemically very similar structure of the monomer units. Furthermore, the success of the copolymerizations was indicated by SEC analysis, showing high molecular weights (*M*_n_ (CP1) = 19.2 kDa, *M*_n_ (CP2) = 15.1 kDa) and dispersities ≈2. The copolymers share the already observed characteristics of the homopolymers, i.e., high thermal stability, high glass transition temperatures, as well as AIE fluorescence. The emission maxima of CP1 and CP2 were observed between those of the respective homopolymers. This is particularly noticeable for CP2, which exhibits greenish‐blue fluorescence at 505 nm (**Figure** **1**). The same trend was observable for the glass transition temperatures of the copolymers with respect to the homopolymers, with CP1 exhibiting a T_g_ of 241 °C (227 and 256 °C for P1 and P2, respectively), and CP2 showing a glass transition at 214 °C (256 and 194 °C for P2 and P5, respectively). These results show that a straightforward copolymerization allows for an easy property adjustment.

## 3 Conclusion

In this manuscript, a new strategy to access poly(arylene‐1,2‐diarylvinylene) (PAV‐DA) derivatives via an elemental sulfur‐mediated polyolefination of bifunctional *N*‐tosylhydrazones is presented. The monomers were easily accessible in good yields by a simple two‐step procedure via Friedel–Crafts acylation and subsequent hydrazone condensation. Eight homopolymers comprising different arylene backbones and aryl side chains were successfully obtained in good yields, exhibiting high molecular weights (*M*_n_ up to 26.4 kDa). The new polyolefination has proven advantageous compared to established literature procedures, providing superior yields and molecular weights for several examples, while also being significantly more practicable due to its bench‐stable reagents and lack of expensive or hazardous materials. All synthesized polymers were fully characterized using ^1^H and ^13^C NMR spectroscopy, IR spectroscopy, SEC, TGA, DSC, and fluorescence spectroscopy, revealing interesting characteristics such as AIE fluorescence and exceptional thermal stability. Furthermore, we have successfully synthesized two PAV‐DA copolymers, allowing for an easy adjustment of the desired polymer properties and thus greatly increasing the feasibility of the new procedure. In light of the promising application possibilities of AIE‐active polymers such as PAV‐DAs, we hope that our new polymerization method will incentivize further studies on these interesting materials due to their newly enhanced accessibility.

## Conflict of Interest

The authors declare no conflict of interest.

## Supporting information



Supporting Information

## Data Availability

The data that support the findings of this study are available in the supplementary material of this article.
