# Supplementary material for: A Novel Synthesis Strategy for Poly(Arylene‐Vinylene) Derivatives by Elemental Sulfur‐Mediated Polyolefination
Source: Macromol Rapid Commun. 2025 Mar 26;46(23):2500166. doi: 10.1002/marc.202500166 (PMC12687711; doi:10.1002/marc.202500166)
Supplement: Supplementary file 1 — Supporting Information [file MARC-46-2500166-s001.pdf]

SUPPLEMENTARY INFORMATION

A novel synthesis strategy for poly(arylene-vinylene) derivatives by elemental sulfur-mediated polyolefination

P. Conen, F. J. O. Niedermaier, S. Abou El Mirate, M. A. R. Meier*

P. Conen, F. J. O. Niedermaier, S. Abou El Mirate, M. A. R. Meier
Institute of Organic Chemistry (IOC)
Karlsruhe Institute of Technology (KIT)
Kaiserstr. 12, 76131 Karlsruhe, Germany
E-mail: m.a.r.meier@kit.edu

P. Conen, M. A. R. Meier
Institute of Biological and Chemical Systems (IBCS-FMS)
Karlsruhe Institute of Technology (KIT)
Kaiserstr. 12, 76131 Karlsruhe, Germany

Table of Contents

1. Methods and Instrumentation 2
2. Materials 4
3. Synthesis and Characterization 5
   3.1 Monomer synthesis 5
      3.1.1 Diketones 5
      3.1.2 *N*-Tosylhydrazones 21
   3.2 Polymer synthesis 37
   3.3 Other syntheses 67
   3.4 Screening Results 76
      3.4.1 Polymerization Screenings 76
      3.4.2 Screening of monofunctional sulfonylhydrazone coupling 78
4. Supplementary References 79

1. Methods and Instrumentation

Nuclear Magnetic Resonance Spectroscopy (NMR)

^{1}H and ^{13}C-NMR spectra were recorded on a *BRUKER Avance 400* NMR spectrometer at 400 MHz for ^{1}H and 101 MHz for ^{13}C-NMR. Unless otherwise stated, all spectra were recorded at ambient temperature. The chemical shift was reported in parts per million (ppm) and referenced to characteristic signals of deuterated solvents, *e.g.* DMSO-d_6 at 2.50 ppm for ^{1}H (39.52 ppm for ^{13}C) and chloroform-d_1 at 7.26 ppm for ^{1}H (77.16 ppm for ^{13}C). For centrosymmetrical signals, the centroid shift was given and for multiplets, the signal range was reported. The multiplets arising from spin-spin coupling were abbreviated as follows: s = singlet, d = doublet, t = triplet, q = quartet, quint. = quintet, sext. = sextet, m = multiplet, br = broad signal. Coupling constants J were reported in Hz. Furthermore, the 2D-NMR methods ^{1}H,^{1}H-correlated spectroscopy (COSY), ^{1}H,^{13}C-heteronuclear single quantum coherence spectroscopy (HSQC), ^{1}H-^{13}C-heteronuclear multiple bond correlation spectroscopy (HMBC) and ^{1}H-^{1}H-nuclear Overhauser enhancement spectroscopy (NOESY) were performed for signal assignment and structure elucidation.

Infrared Spectroscopy (IR)

IR measurements were performed on a *Bruker ALPHA attenuated total reflection (ATR)* IR spectrometer in a range from 4000 to 500 cm^{-1} at ambient temperature. The bands were characterized according to their transmission (T) intensity as follows: vs = very strong (0 – 10% T), s = strong (10 – 40% T), m = medium (40 – 70% T), w = weak (70 – 90% T), vw = very weak (>90% T). Broad signals were labelled with br.

UV/Vis Spectroscopy

Absorption spectroscopy was performed on an *Agilent Cary 3500 UV-Vis* multicell spectrometer with a Peltier Element. Spectra were recorded at a cell temperature of 20 °C in a range of 200 nm to 800 nm in 1 nm intervals using a scanning speed of 600 nm/min. Samples were measured using 1 cm *STARNA* quartz cuvettes in a concentration of 100 µM in tetrahydrofuran.

Fluorescence Spectroscopy

Emission spectra were recorded on a *HORIBA FluoroMax Plus* spectrometer. Spectra were recorded at a cell temperature of 20°C in 1 nm intervals using an increment time of 200 ms and an integration time of 100 ms. Excitations wavelengths and slit sizes were adapted for each sample individually. Samples were prepared by injecting 10 µL of a 10 mM stock solution of the analyte in THF into 5 mL of water with 10 vol% THF under rapid stirring (corresponding to 20 µM of analyte in water with 10 vol% THF). For further details, see Chapter 3.7.

Thin Layer Chromatography (TLC)

TLC analyses were performed using aluminium plates coated with silica and a fluorescence indicator (*Merck, Silica Gel 60, F 254, layer thickness 0.25 mm*). The spots were visualized by examining their fluorescence under UV light (λ = 365 nm) or their fluorescence quenching at a wavelength of 254 nm. If necessary, the TLC plates were stained with a potassium permanganate staining solution (1.50 g $KMnO_4$, 10.0 g K_2CO_3 and 0.50 g NaOH in 200 mL water).

Column Chromatography

Column Chromatography was performed as the primary workup method using *Silica Gel 60* (*Merck*, mesh size 40 – 63 µm) and quartz sand (*Bernd Kraft*) as column material and HPLC grade solvents as mobile phase.

Size Exclusion Chromatography (SEC)

SEC measurements were performed on a Shimadzu SEC system equipped with a Shimadzu isocratic pump (LC-20AD), a Shimadzu refractive index detector (30 °C) (RID-20A), a Shimadzu 164 autosampler (SIL-20A) and a Shimadzu column oven (30 °C). The column system comprised a SDV 5 µm, 8×50 mm precolumn, a SDV 5 µm, 1,000 A, 8×300 mm column and a SDV 5 µm, 100,000 A, 8×300 mm column supplied by PSS, Germany. A mixture of THF stabilized with 250 ppm butylated hydroxytoluene (≥99.9%) and 2 vol% triethylamine (≥99.5%) supplied by Sigma Aldrich was used at a flow rate of 1.00 mL/min. Calibration was carried out by injection of ten narrow PMMA standards ranging from 1,102 to 981,000 kDa.

Thermogravimetric Analysis (TGA)

Thermogravimetric analysis was performed on a TGA5500 from TA instruments. 5-15 mg of a sample were placed in an aluminum pan and heated at a rate of 10 K min−1 from ambient temperature to 600-1000 °C under nitrogen flow. The onset was evaluated using Trios v5.0.044608 software.

Differential Scanning Calorimetry (DSC)

DSC experiments were performed on a Mettler Toledo DSC 3 using a Huber Intracooler TC100 and aluminum crucibles (40 and 100 µL). Measurements were performed under nitrogen flow (50 mL min^{-1}) in three consecutive heating-cooling cycles from 25 °C up to 300 °C with a heating rate of 20 K min^{-1}. Each measurement was performed using 3-7 mg of substance for sample preparation. T_g values were determined as the onset of the transition in the second heating cycle.

2. Materials

Unless otherwise specified, all chemicals were used as received.

Substance	Supplier	Purity
Terephthaloyl chloride	TCI	>99%
Anisole	Sigma-Aldrich	99%
Aluminium chloride	Acros Organics	98.5%, anhydrous
Sodium hydroxide	Bernd Kraft	p.a.
Ethanol	VWR	HPLC grade
Benzene	TCI	<99.5%
Diphenyl ether	Fisher Scientific	<99%
Diphenyl sulfide	ChemPur	98%
Isophthaloyl chloride	Fisher Scientific	98%
4,4'-Biphenyldicarboxylic acid	ChemPur	98%
Oxalyl chloride	Sigma-Aldrich	98%
N,N-Dimethylformamide	VWR	HPLC grade
Dichloromethane	VWR	HPLC grade
2,5-Thiophenedicarboxylic acid	ChemPur	98%
tert-Butylbenzene	Fisher Scientific	99%
Tosyl hydrazide	Sigma-Aldrich	97%
p-Toluenesulfonic acid monohydrate	Acros Organics	>99%, extra pure
Toluene	VWR	HPLC grade
Dimethylsulfoxide	Acros Organics	99.7%, extra dry
Dimethylsulfoxide-d_6	Eurisotop	>99.9 atom-% D
Chloroform-*d*	Eurisotop	>99.9 atom-% D
Chloroform	VWR	HPLC grade
Sulfur	BASF	Technical
Potassium carbonate	Fisher Scientific	>99%, anhydrous
4,4'-Dimethoxybenzophenone	Acros Organics	97%
Hydrazine hydrate	Sigma-Aldrich	99%
p-Nosyl chloride	Sigma-Aldrich	97%
o-Nosyl chloride	TCI	97%
Trisyl Chloride	Sigma-Aldrich	97%
Tetrahydrofuran	Sigma Aldrich	>99,8%, distilled before use
Ethyl acetate	VWR	HPLC grade
Cyclohexane	VWR	HPLC grade
Methanol	VWr	HPLC grade
Acetonitrile	Fisher Scientific	HPLC grade
Triglyme	Sigma-Aldrich	99%
Ethylene glycol	Riedel-de Haën	>99.5%
2-Methyltetrahydrofuran	Acros Organics	Anhydrous
Dimethyl carbonate	Sigma-Aldrich	>99%

3. Synthesis and Characterization

3.1 Monomer synthesis

The procedures for diketone syntheses were adapted from Carreira *et al*.[1]
The procedure for tosylhydrazone syntheses were adapted from Meier *et al*.[2]

3.1.1 Diketones

Synthesis of 4,4'-dimethoxyterephthalophenone (**K1**)

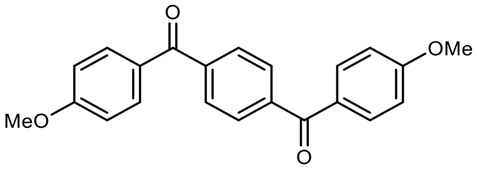

In a Schlenk flask under argon atmosphere, terephthaloyl chloride (5.00 g, 24.6 mmol, 1.00 equiv.) was suspended in anisole (53.3 g, 53.8 mL, 493 mmol, 20.0 equiv). The mixture was placed in an ice bath and aluminium chloride (7.88 g, 59.1 mmol, 2.40 equiv.) was added in small portions. The mixture was stirred at room temperature for 16 hours. The crude mixture was subsequently poured onto ice. The precipitate was filtered, washed twice with 10% NaOH, once with water, once more with EtOH and dried *in vacuo*. **K1** was obtained as a light pink solid in a yield of 77% (6.58 g, 19.0 mmol).

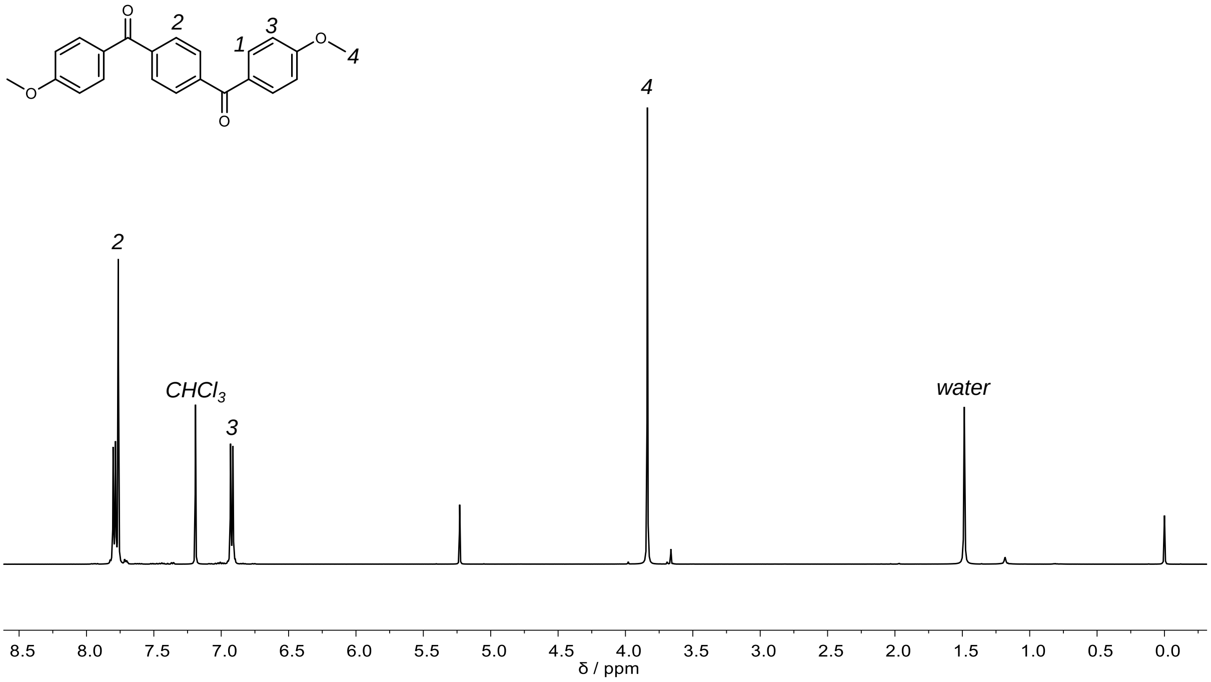

^{1}H NMR (500 MHz, Chloroform-*d*): δ/ppm = 7.82 – 7.78 (m, 4H), 7.76 (s, 4H), 6.95 – 6.89 (m, 4H), 3.84 (s, 6H).

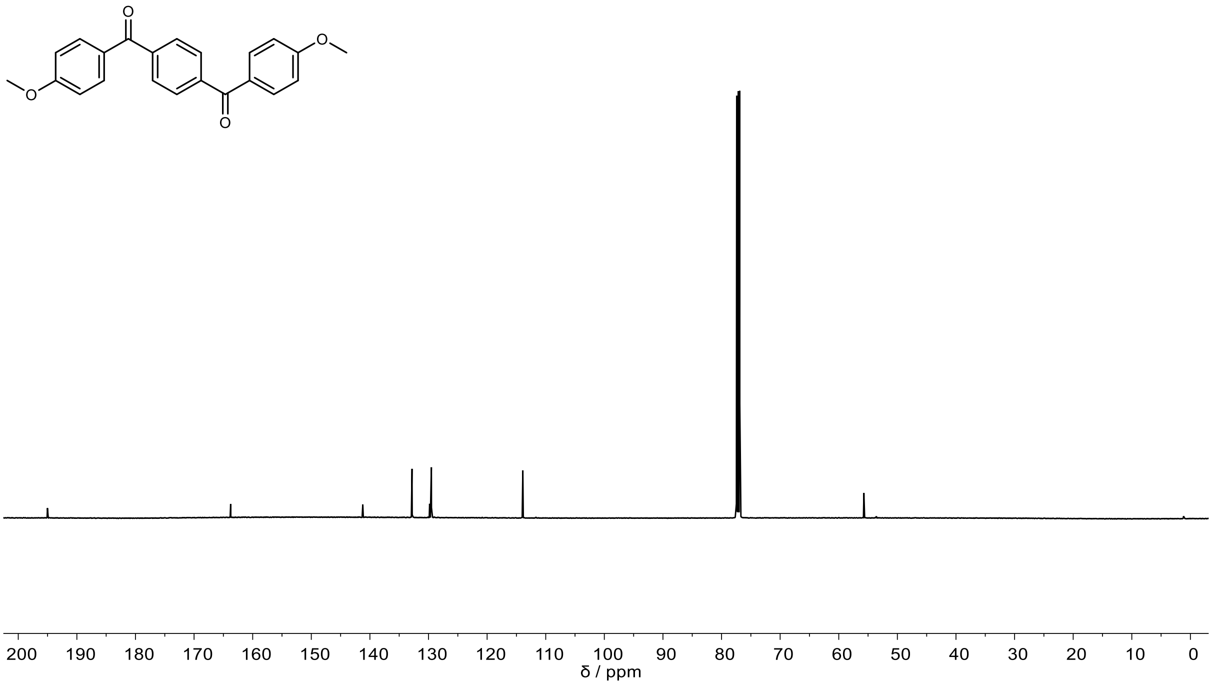

^{13}C NMR (126 MHz, Chloroform-*d*): δ/ppm = 195.02, 163.76, 141.21, 132.81, 129.81, 129.51, 113.91, 55.71.

IR (ATR platinum diamond): ν/cm^{-1} = 3069 (vw), 3061 (vw), 2976 (vw), 2950 (vw), 2925 (vw), 2843 (vw), 1639 (s), 1594 (s), 1510 (m), 1493 (w), 1469 (w), 1458 (w), 1440 (w), 1421 (vw), 1397 (w), 1341 (w), 1318 (s), 1302 (m), 1277 (m), 1261 (vs), 1195 (m), 1177 (s), 1154 (s), 1113 (m), 1082 (vw), 1016 (s), 977 (vw), 969 (w), 928 (s), 860 (m), 845 (s), 839 (m), 821 (w), 796 (m), 749 (s), 691 (s), 638 (w), 627 (m), 578 (m), 522 (w), 514 (w), 434 (m), 416 (w), 405 (w).

Synthesis of terephthalophenone (**K2**)

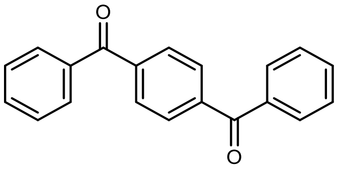

In a Schlenk flask under argon atmosphere, terephthaloyl chloride (2.00 g, 9,85 mmol, 1.00 equiv.) was suspended in benzene (17.5 mL, 15.4 g, 197 mmol, 20.0 equiv.). The mixture was placed in a water bath and aluminium chloride (2.89 g, 21.7 mmol, 2.20 equiv.) was added in small portions. The mixture was stirred at room temperature for 16 hours. The crude mixture was subsequently poured onto ice. The precipitate was filtered, washed twice with 10% NaOH, once with water, once more with EtOH and dried *in vacuo*. **K1** was obtained as a white solid in a yield of 73% (2.06 g, 7.21 mmol).

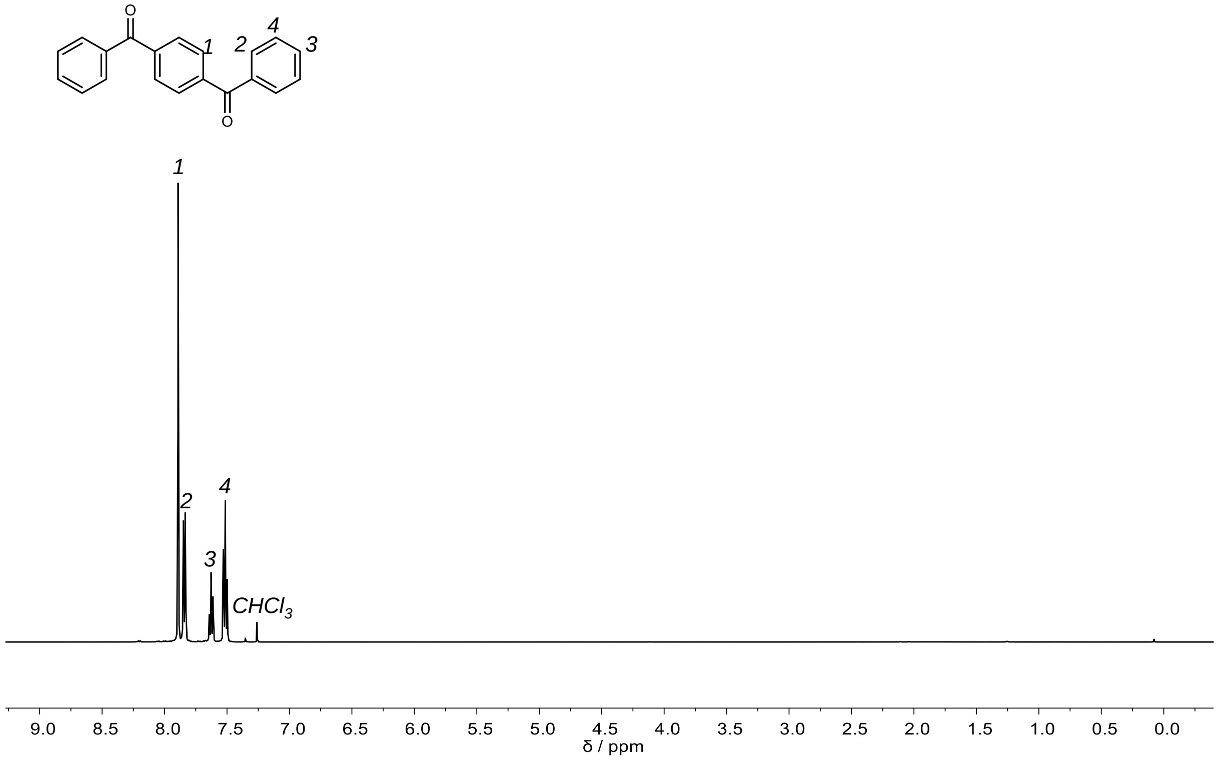

^{1}H NMR (500 MHz, Chloroform-*d*): δ/ppm = 7.89 (s, 4H), 7.86 – 7.82 (m, 4H), 7.65 – 7.60 (m, 2H), 7.51 (t, *J* = 7.8 Hz, 4H).

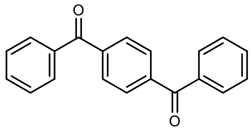

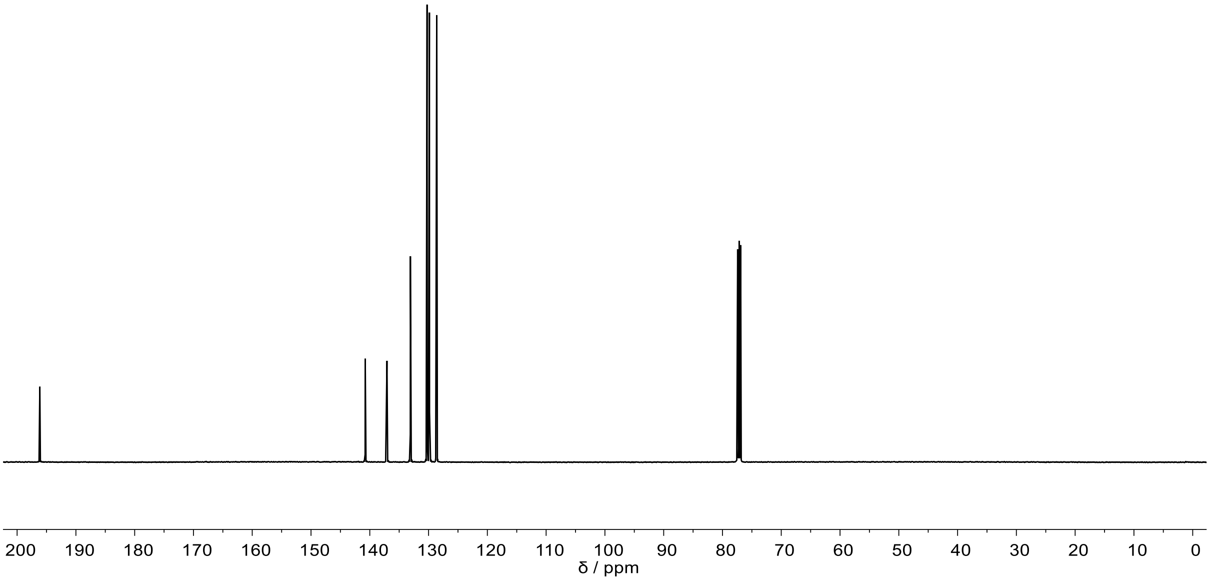

^{13}**C NMR** (126 MHz, Chloroform-*d*): δ/ppm = 196.14, 140.78, 137.08, 133.09, 130.25, 129.86, 128.61.

IR (ATR platinum diamond): *v*/cm^{-1} = 1654 (s), 1594 (m), 1576 (w), 1539 (w), 1500 (w), 1446 (w), 1401 (w), 1382 (w), 1310 (m), 1288 (w), 1263 (m), 1203 (w), 1181 (m), 1156 (m), 1109 (w), 1086 (w), 1074 (w), 1022 (w), 998 (w), 977 (w), 969 (w), 938 (m), 922 (m), 856 (m), 847 (m), 782 (m), 732 (w), 706 (s), 691 (vs), 675 (s), 619 (w), 584 (m), 522 (w), 465 (m), 450 (m), 418 (w).

Synthesis of 4,4'-diphenoxyterephthalophenone (**K3**)

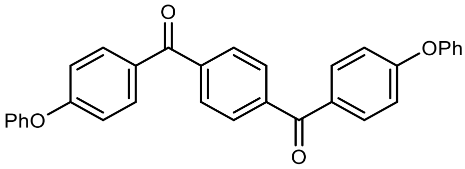

In a Schlenk flask under argon atmosphere, terephthaloyl chloride (2.00 g, 9.85 mmol, 1.00 equiv.) was suspended in diphenyl ether (15.7 mmol, 16.8 g, 98.5 mmol, 10.0 equiv.). The mixture was placed in an ice bath and aluminium chloride (2.89 g, 21.7 mmol, 2.20 equiv.) was added in small portions. The mixture was stirred at room temperature for 16 hours. The crude mixture was subsequently poured onto ice. The precipitate was filtered, washed twice with 10% NaOH, once with water, once more with EtOH and dried *in vacuo*. **K3** was obtained as a white solid in a yield of 65% (3.03 g, 6.44 mmol).

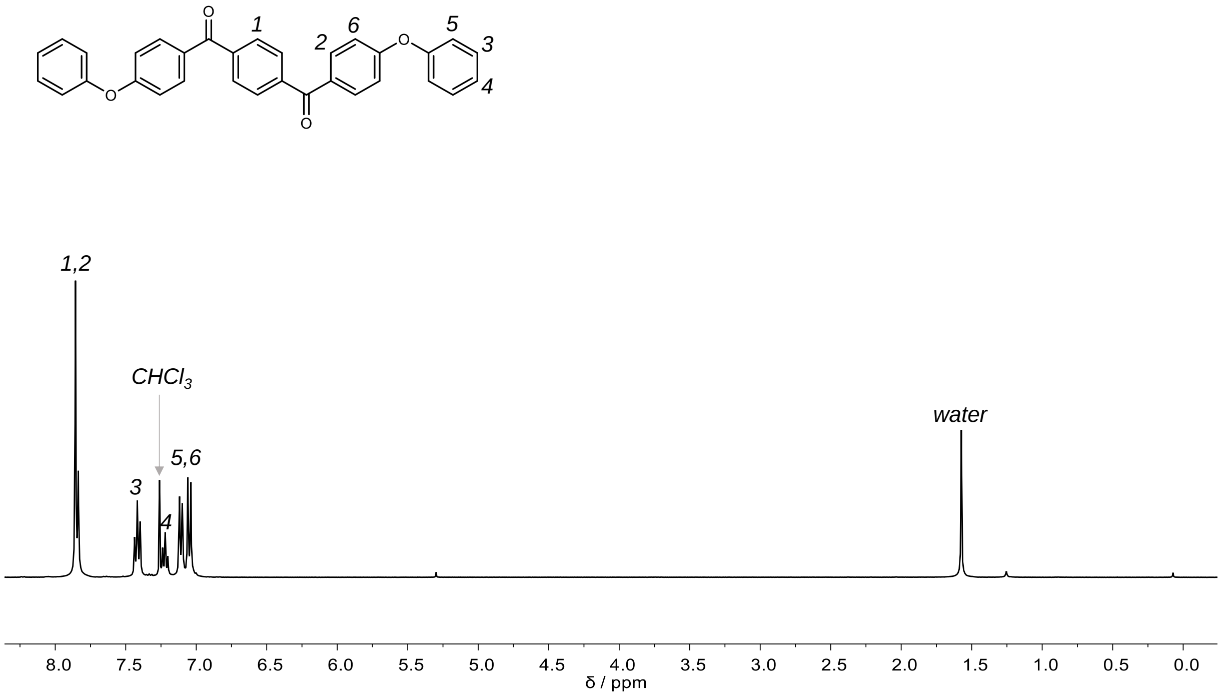

^{1}H NMR (400 MHz, Chloroform-*d*): δ/ppm = 7.85 (d, *J* = 8.0 Hz, 8H), 7.48 – 7.35 (m, 4H), 7.25 – 7.19 (m, 2H), 7.14 – 7.08 (m, 4H), 7.08 – 7.02 (m, 4H).

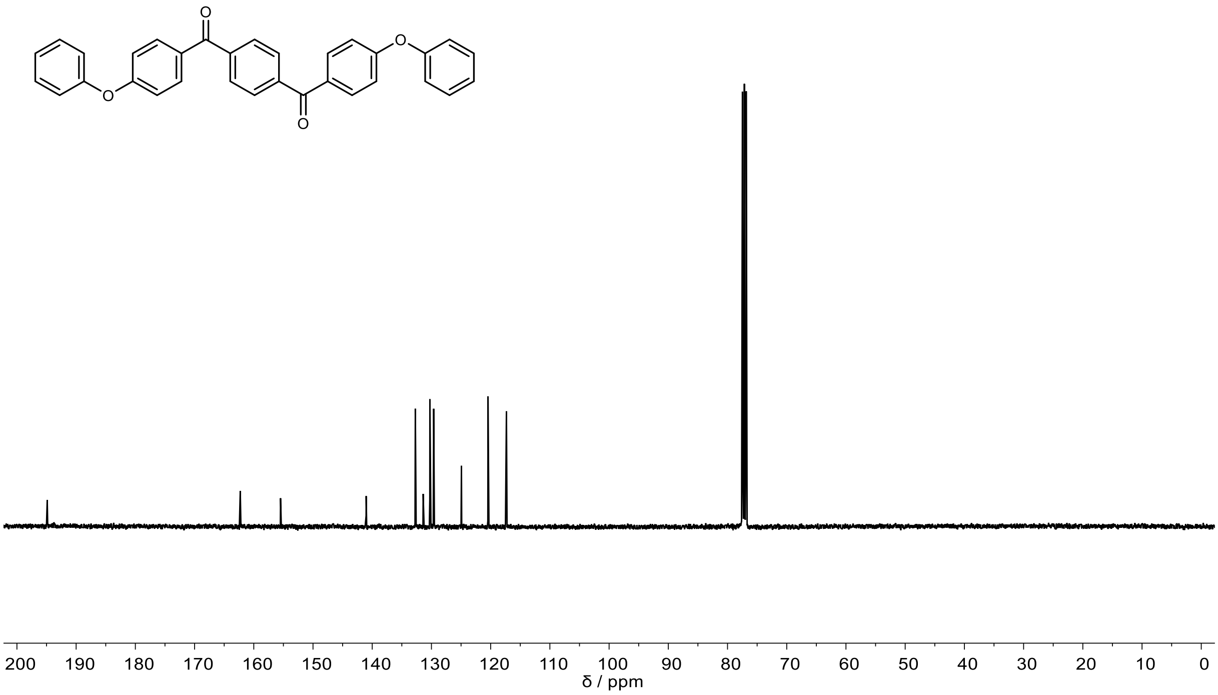

^{13}C NMR (101 MHz, Chloroform-*d*): δ/ppm = 194.88, 162.28, 155.48, 141.02, 132.72, 131.39, 130.26, 129.62, 124.93, 120.44, 117.34.

IR (ATR platinum diamond): ν/cm^{-1} = 3065 (vw), 3038 (vw), 1643 (s), 1586 (s), 1487 (m), 1454 (w), 1413 (w), 1401 (w), 1378 (w), 1304 (m), 1286 (m), 1251 (s), 1197 (m), 1152 (s), 1119 (m), 1109 (m), 1072 (m), 1016 (w), 1004 (w), 977 (w), 961 (w), 926 (m), 901 (m), 872 (m), 864 (m), 839 (s), 829 (m), 792 (m), 743 (vs), 687 (vs), 658 (m), 638 (w), 611 (w), 597 (w), 570 (w), 508 (m), 500 (m), 492 (m), 465 (w), 432 (w), 409 (vw).

Synthesis of 4,4'-bis(phenylthio)terephthalophenone (**K4**)

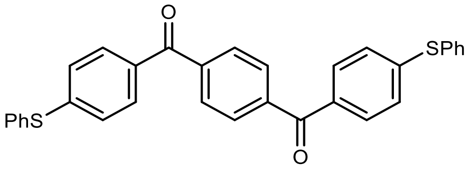

In a Schlenk flask under argon atmosphere, terephthaloyl chloride (1.00 g, 4.93 mmol, 1.00 equiv.) was suspended in diphenyl sulfide (8.34 mL, 9.78 g, 49.3 mmol, 10.0 equiv.). The mixture was placed in an ice bath and aluminium chloride 1.45 g (10.8 mmol, 2.20 g) was added in small portions. The mixture was stirred at room temperature for 16 hours. The crude mixture was subsequently poured onto ice. The precipitate was filtered, washed twice with 10% NaOH, once with water, once more with EtOH and dried *in vacuo*. **K4** was obtained as a white solid in a yield of 62% (1.52 g, 3.03 g).

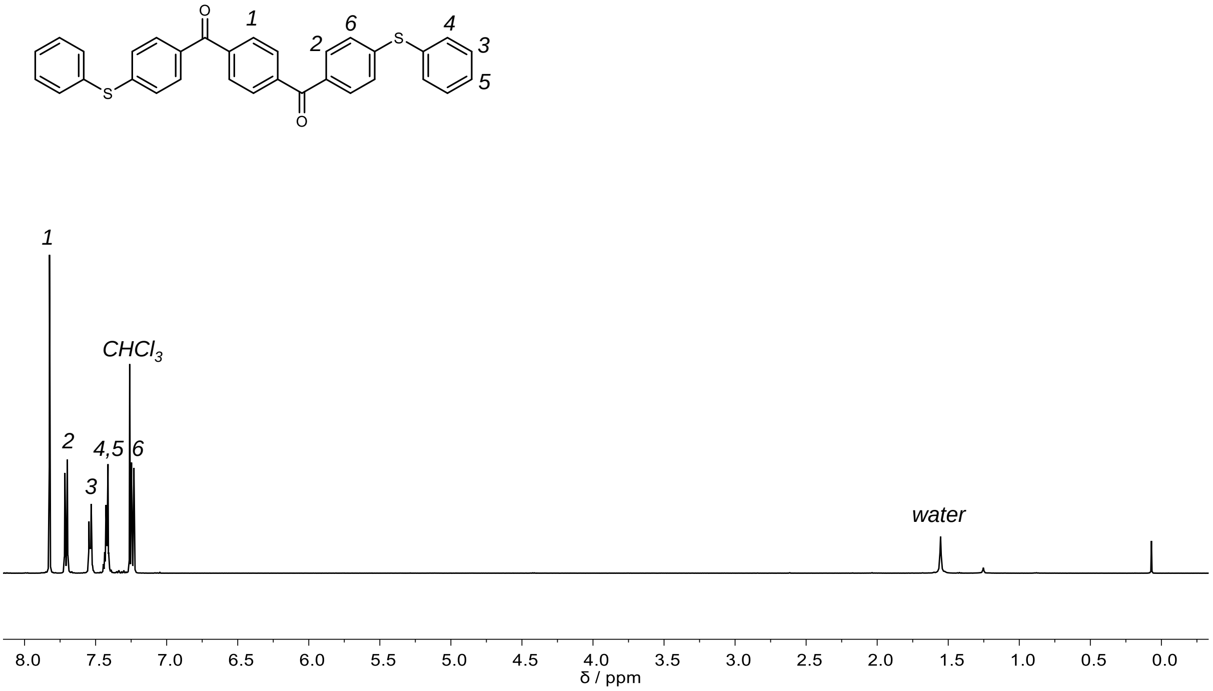

^{1}H NMR (500 MHz, Chloroform-*d*): δ/ppm 7.82 (s, 4H), 7.73 – 7.68 (m, 4H), 7.55 – 7.51 (m, 4H), 7.41 (m, 6H), 7.25 – 7.21 (m, 4H).

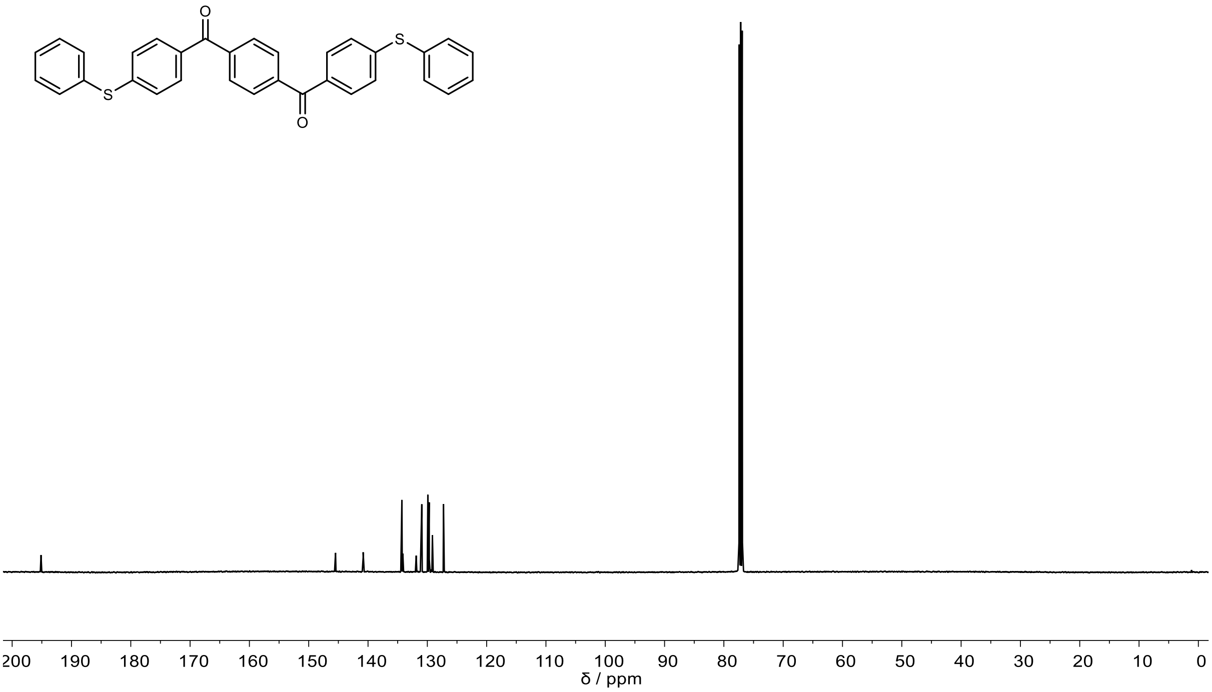

^{13}**C NMR** (126 MHz, Chloroform-*d*): δ 195.13, 145.48, 140.82, 134.29, 134.13, 131.88, 130.93, 129.91, 129.68, 129.14, 127.27.

IR (ATR platinum diamond): $\tilde{v}$/cm^{-1} = 3057 (vw), 1643 (s), 1586 (m), 1549 (w), 1497 (vw), 1485 (vw), 1475 (w), 1440 (w), 1399 (w), 1306 (m), 1273 (s), 1183 (w), 1156 (w), 1121 (vw), 1078 (m), 1022 (w), 1012 (w), 1000 (vw), 977 (vw), 963 (w), 924 (s), 860 (m), 841 (w), 827 (m), 749 (w), 734 (vs), 695 (w), 683 (vs), 631 (w), 617 (vw), 597 (vw), 516 (w), 498 (w), 483 (w), 422 (w), 411 (vw).

Synthesis of isophthalophenone (**K5**)

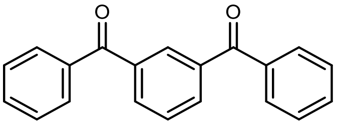

In a Schlenk flask under Ar atmosphere, isophthaloyl chloride (2.00 g, 9.85 mmol, 1.00 equiv.) was suspended in benzene (17.5 mL, 15.4 g, 197 mmol, 20.0 equiv.). The mixture was placed in a water bath and aluminium chloride (2.89 g, 21.7 mmol, 2.20 equiv.) was added in small portions. The mixture was stirred at room temperature for 16 hours. The crude mixture was subsequently poured onto ice. The precipitate was filtered, washed twice with 10% NaOH, once with water, once more with EtOH and dried *in vacuo*. **K5** was obtained as a white solid in a yield of 83% (2.35 g, 8.20 mmol).

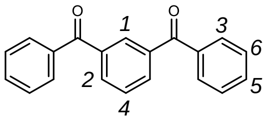

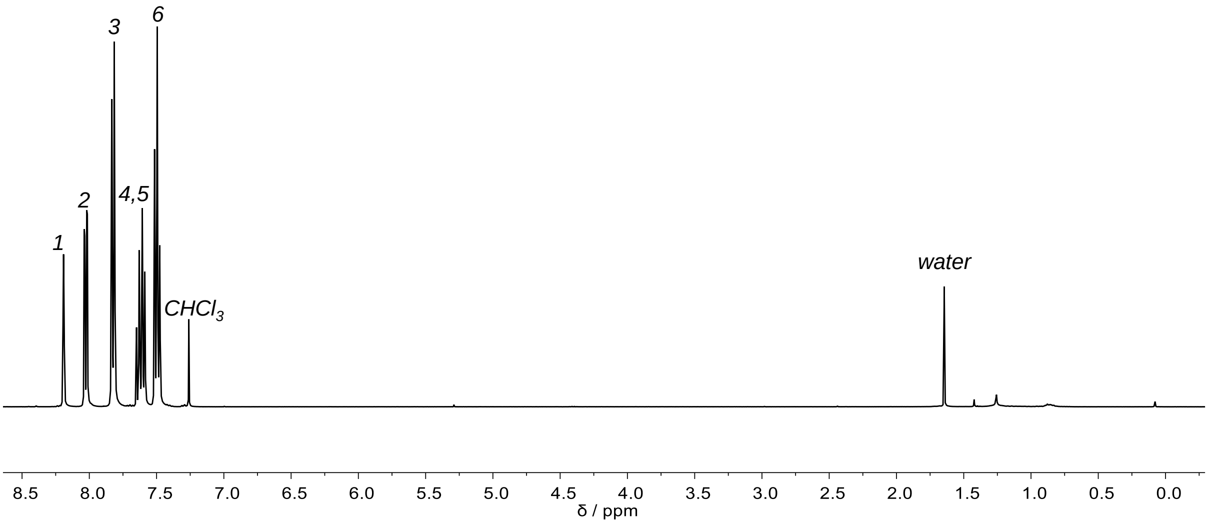

^{1}H NMR (400 MHz, Chloroform-*d*): δ/ppm = 8.21 – 8.17 (m, 1H), 8.03 (dd, *J* = 7.7, 1.8 Hz, 2H), 7.86 – 7.78 (m, 4H), 7.67 – 7.57 (m, 3H), 7.54 – 7.45 (m, 4H).

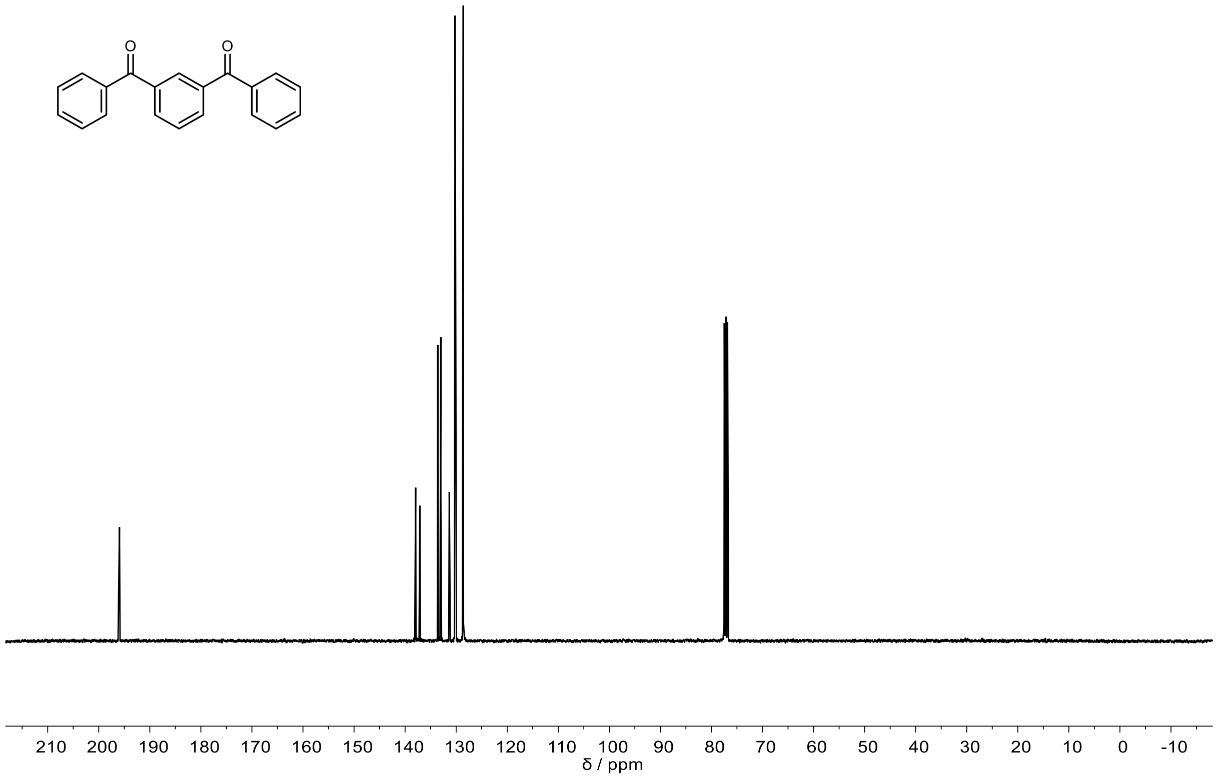

^{13}C NMR (101 MHz, Chloroform-*d*): δ/ppm = 195.94, 137.93, 137.11, 133.61, 133.01, 131.34, 130.21, 128.65, 128.61.

IR (ATR platinum diamond): ν/cm^{-1} = 3063 (vw), 3052 (vw), 3036 (vw), 3024 (vw), 2923 (vw), 1654 (s), 1594 (m), 1576 (m), 1514 (vw), 1467 (vw), 1444 (m), 1397 (vw), 1318 (w), 1310 (w), 1286 (m), 1265 (m), 1247 (m), 1166 (m), 1115 (m), 1076 (w), 1002 (m), 991 (m), 973 (w), 946 (w), 930 (w), 909 (w), 850 (vw), 821 (w), 798 (w), 778 (m), 757 (w), 689 (vs), 648 (m), 638 (m), 617 (w), 592 (w), 555 (w), 510 (vw), 446 (w), 438 (w), 420 (vw), 405 (vw).

Synthesis of 4,4'-bis(benzoyl)biphenyl (**K6**)

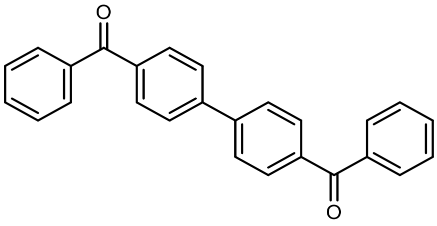

In a Schlenk flask under argon atmosphere, 4,4'-biphenyldicarboxylic acid (2.42 g, 9.99 mmol, 1.00 equiv.) was dissolved in 8 mL of dichloromethane and oxalyl chloride (2.23 mL, 3.30 mg, 26.0 mmol, 2.60 equiv.) was added. The mixture was stirred at room temperature for 30 minutes and then refluxed for 2 hours. Afterwards, the solvent was removed *in vacuo*. The residue was subsequently dissolved in benzene (17.9 mL, 15.6 g, 200 mmol, 20.0 equiv.). The mixture was placed in a water bath and aluminium chloride (2.93 g, 22.0 mmol, 2.20 equiv) was added in small portions. The mixture was stirred at room temperature for 16 hours. The crude mixture was subsequently poured onto ice. The precipitate was filtered, washed twice with 10% NaOH, once with water, once more with EtOH and dried *in vacuo*. **K6** was obtained as a white solid in a yield of 82% (2.96 mmol, 81.6 mmol).

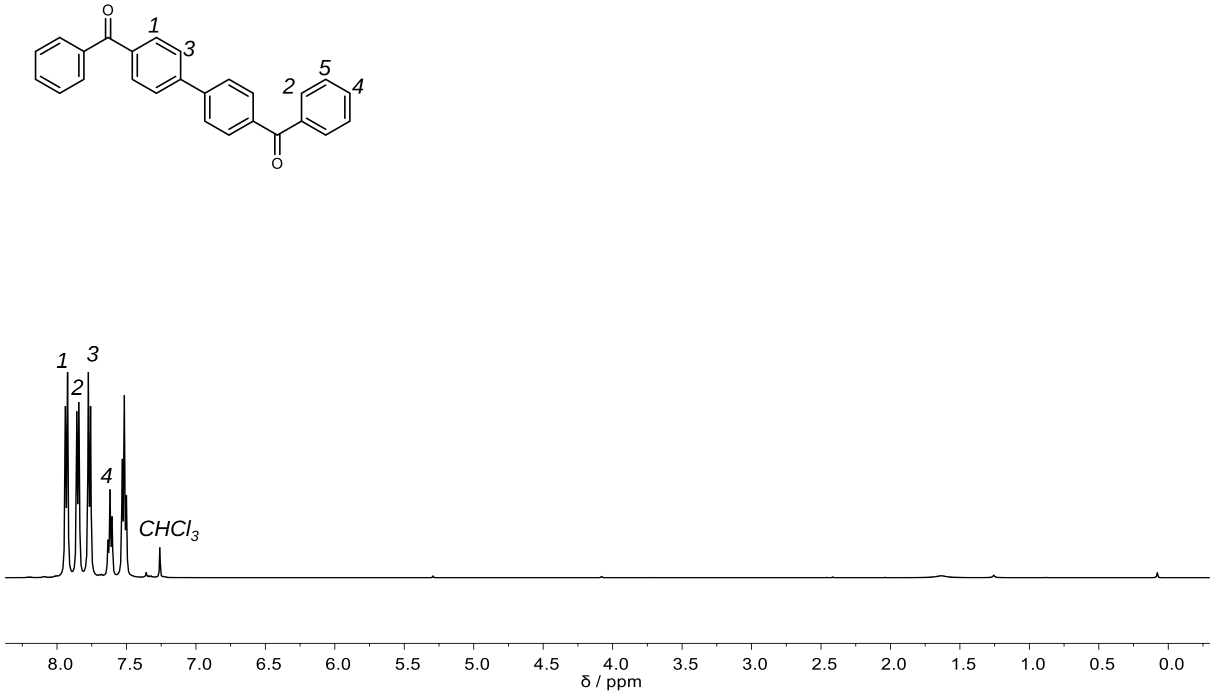

^{1}H NMR (500 MHz, Chloroform-*d*): δ/ppm 7.97 – 7.89 (m, 4H), 7.88 – 7.82 (m, 4H), 7.80 – 7.73 (m, 4H), 7.65 – 7.58 (m, 2H), 7.55 – 7.48 (m, 4H).

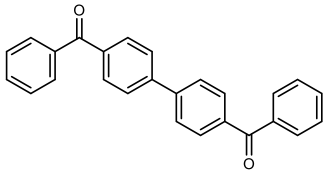

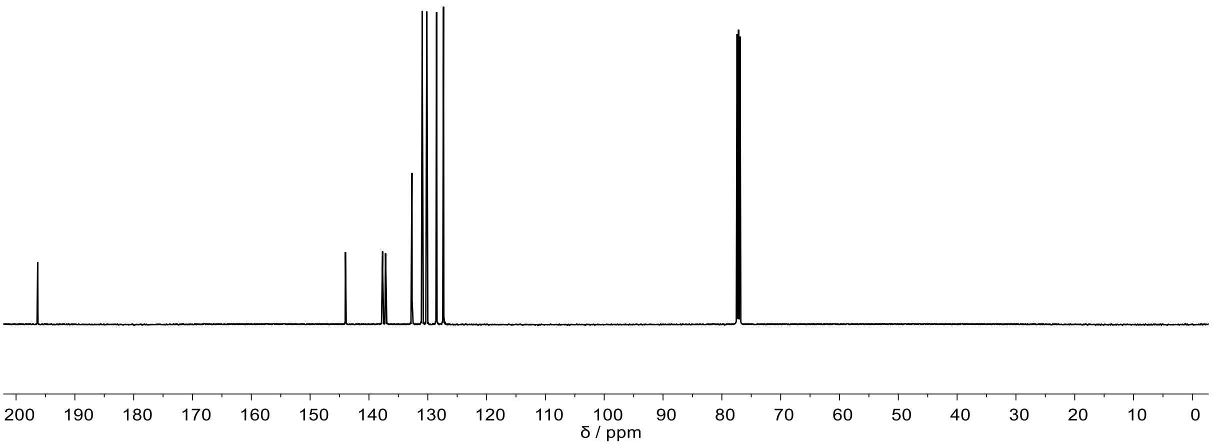

^{13}C NMR (126 MHz, Chloroform-*d*): δ/ppm = 196.32, 144.00, 137.68, 137.18, 132.68, 130.93, 130.16, 128.51, 127.32.

IR (ATR platinum diamond): ν/cm^{-1} = 3050 (vw), 3036 (vw), 1643 (s), 1602 (m), 1596 (m), 1578 (w), 1549 (w), 1491 (vw), 1444 (w), 1411 (vw), 1395 (w), 1380 (vw), 1368 (vw), 1331 (w), 1316 (w), 1306 (w), 1284 (m), 1179 (w), 1152 (w), 1111 (vw), 1074 (w), 1026 (w), 1000 (w), 987 (vw), 971 (w), 938 (m), 919 (m), 841 (m), 788 (m), 734 (m), 714 (m), 691 (vs), 644 (w), 636 (m), 617 (w), 566 (vw), 450 (w).

Synthesis of 2,5-bis(benzoyl)thiophene (**K7**)

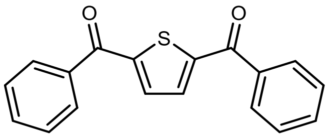

In a Schlenk flask under argon atmosphere, 2,5-thiophenedicarboxylic acid (2.00 g, 11.6 mmol, 1.00 equiv.) was dissolved in 20 mL dichloromethane and oxalyl chloride (2.59 mL, 3.83 g, 30.2 mmol, 2.60 equiv.) was added. The mixture was stirred at room temperature for 30 minutes and subsequently refluxed for 2 hours. Afterwards, the solvent was removed *in vacuo*. The residue was subsequently dissolved in in benzene (20.8 mL, 18.2 g., 232 mmol, 20.0 equiv.). The mixture was placed in a water bath and aluminium chloride (3.41 g, 25.6 mmol, 2.20 equiv.) was added in small portions. The mixture was stirred at room temperature for 16 hours. The crude mixture was subsequently poured onto ice. The precipitate was filtered, washed twice with 10% NaOH, once with water, once more with EtOH and dried *in vacuo*. **K7** was obtained as a white solid in a yield of74% (2.50 g, 8.57 mmol).

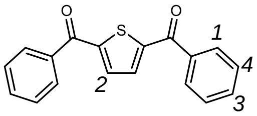

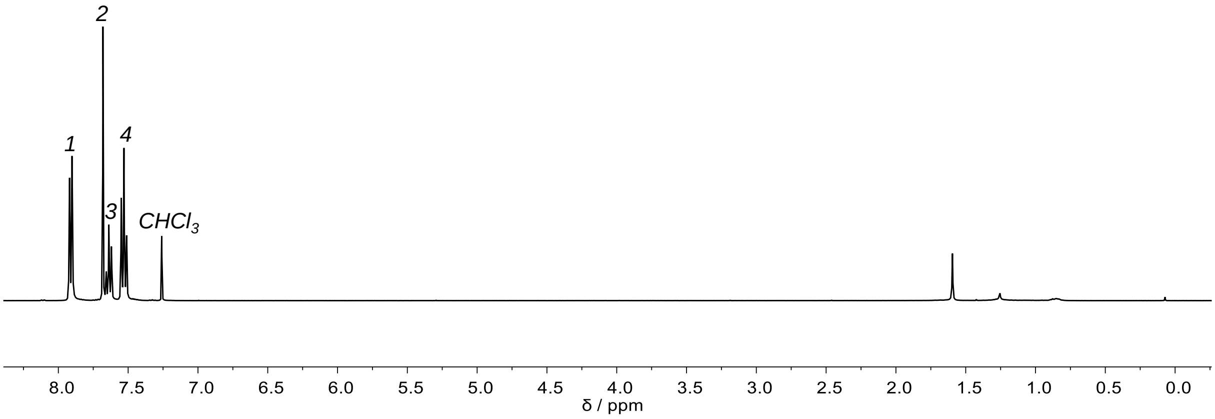

^{1}H NMR (400 MHz, Chloroform-*d*): δ/ppm = 7.94 – 7.88 (m, 4H), 7.68 (s, 2H), 7.66 – 7.60 (m, 2H), 7.56 – 7.49 (m, 4H).

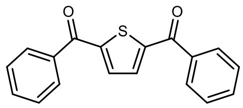

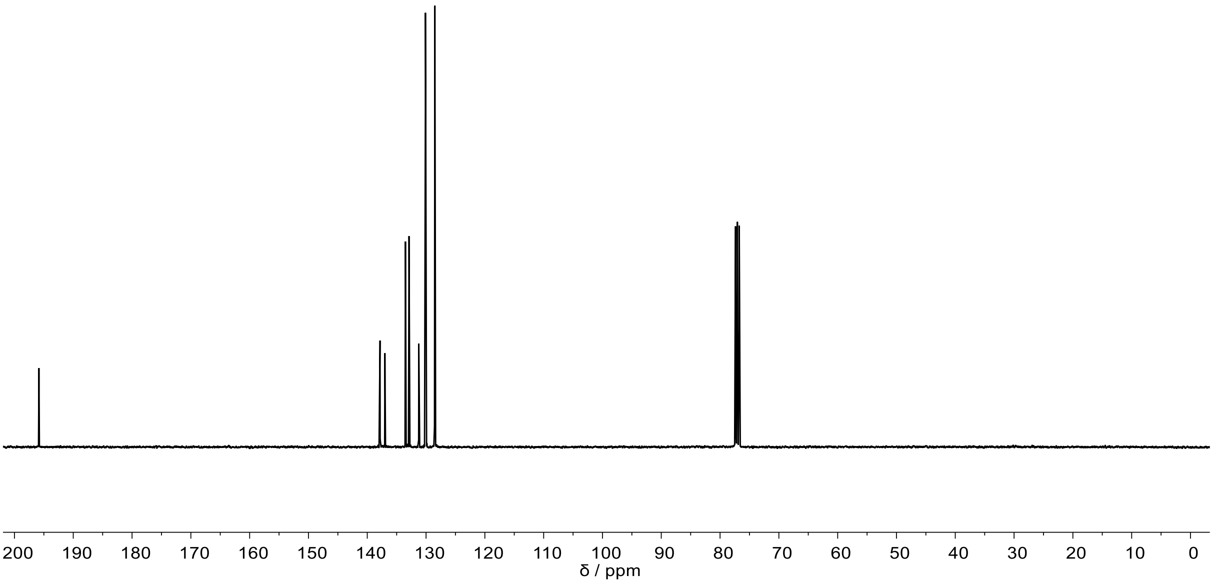

^{13}C NMR (101 MHz, Chloroform-*d*): δ/ppm = 195.83, 137.83, 137.00, 133.50, 132.90, 131.23, 130.10, 128.55, 128.50.

IR (ATR platinum diamond): *v*/cm^{-1} = 3116 (vw), 3057 (vw), 1631 (s), 1596 (m), 1576 (m), 1512 (m), 1444 (m), 1405 (vw), 1345 (w), 1316 (m), 1286 (m), 1275 (m), 1253 (m), 1216 (m), 1203 (w), 1177 (w), 1127 (m), 1076 (m), 1059 (w), 1049 (w), 1022 (w), 1000 (w), 979 (vw), 969 (vw), 936 (vw), 928 (vw), 911 (w), 880 (m), 868 (w), 858 (w), 843 (w), 831 (w), 788 (w), 749 (vw), 716 (m), 701 (vs), 691 (s), 673 (s), 654 (m), 615 (w), 588 (w), 555 (w), 535 (w), 465 (w).

*Synthesis of 4,4'-bis(*tert*-butyl)terephthalophenone* (**K8**)

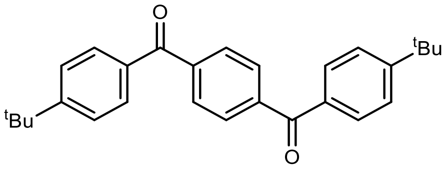

In a Schlenk flask under argon atmosphere, terephthaloyl chloride (2.00 g, 9.85 mmol, 1.00 equiv.) was suspended in *tert*-butylbenzene (30.4 mL, 26.4 g, 197 mmol, 20.0 equiv.). The mixture was placed in a water bath and aluminium chloride (2.89 g, 21.7 mmol, 2.20 equiv.) was added in small portions. The mixture was stirred at room temperature for 16 hours. The crude mixture was subsequently poured onto ice. The precipitate was filtered, washed twice with 10% NaOH, once with water, once more with EtOH and dried in vacuo. **K8** was obtained as a white solid in a yield of 34% (1.45 g, 3.65 mmol).

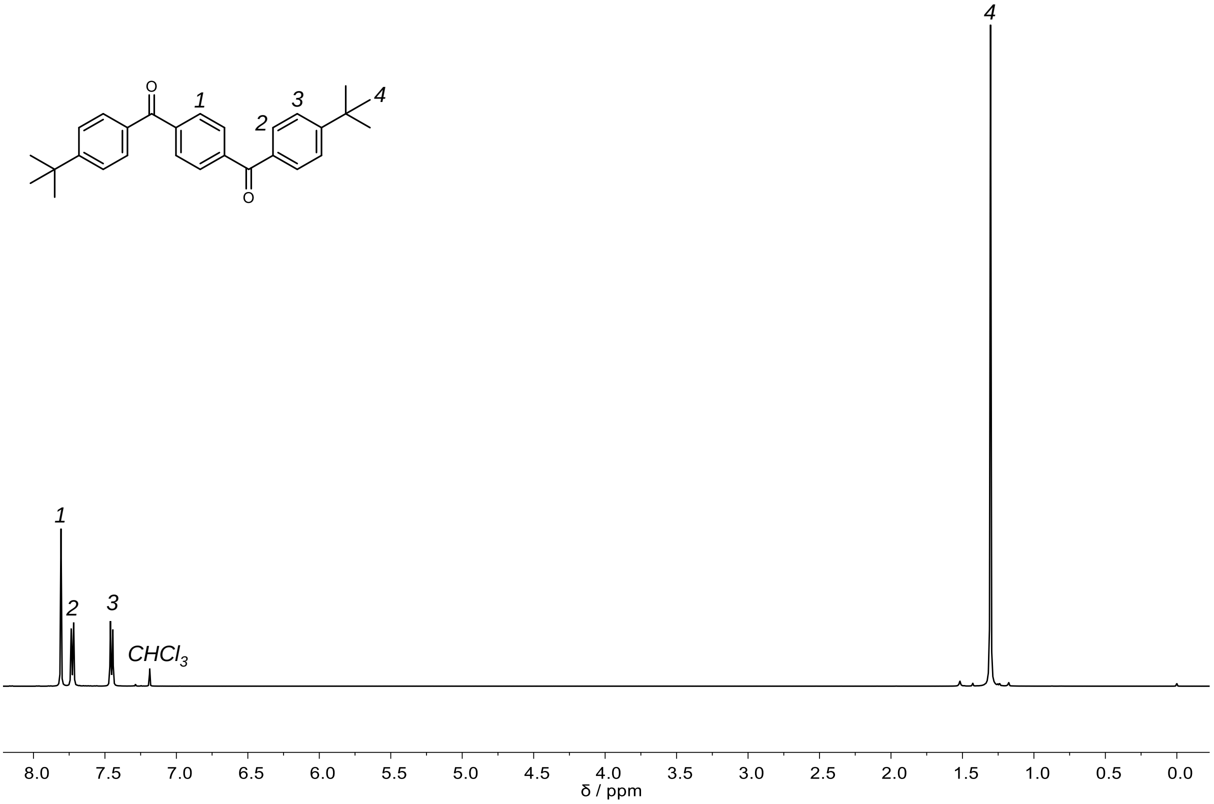

^{1}H NMR (500 MHz, Chloroform-*d*): δ/ppm = 7.81 (s, 4H), 7.76 – 7.68 (m, 4H), 7.50 – 7.40 (m, 4H), 1.30 (s, 18H).

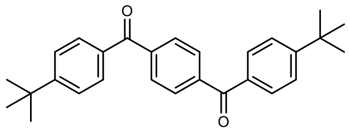

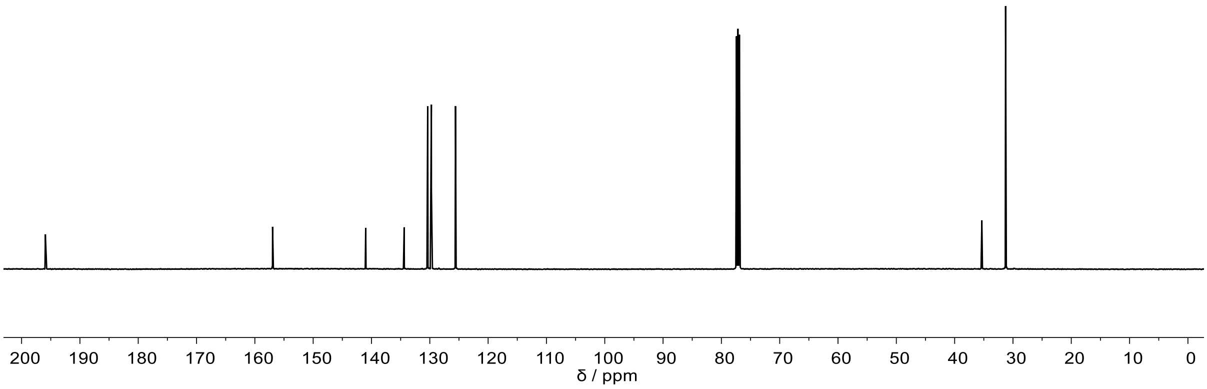

^{13}C NMR (126 MHz, Chloroform-*d*): δ/ppm = 195.92, 156.96, 140.99, 134.40, 130.36, 129.73, 125.59, 35.34, 31.26.

IR (ATR platinum diamond): *v*/cm^{-1} = 2960 (w), 2902 (w), 2865 (vw), 1643 (vs), 1604 (s), 1559 (w), 1497 (w), 1469 (vw), 1460 (vw), 1405 (w), 1360 (w), 1314 (m), 1298 (m), 1277 (s), 1195 (w), 1158 (w), 1127 (vw), 1105 (m), 1024 (w), 1016 (w), 979 (vw), 971 (w), 928 (vs), 862 (m), 837 (s), 761 (m), 734 (w), 695 (s), 640 (vw), 613 (vw), 564 (w), 543 (m), 473 (vw), 455 (vw), 442 (m), 416 (vw).

3.1.2 *N*-Tosylhydrazones

<u>General procedure for the synthesis of bifunctional *N*-tosylhydrazones:</u>

In a glass pressure vial, the respective diketone was suspended in toluene (c = 0.25 M) and tosyl hydrazide (2.20 equiv.) along with *p*-toluenesulfonic acid monohydrate (0.10 equiv.) was added. The vial was closed and stirred at 80 °C for 16 hours. Afterwards, the mixture was cooled to room temperature, the precipitate was filtered, washed with cold ethanol and dried under vacuum. If necessary, the product was further purified via column chromatography.

Synthesis of 4,4'-dimethoxyterephthalophenone bisNTH (**M1**)

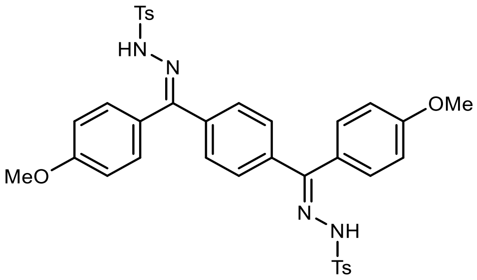

Synthesized from **K1** according to general procedure in a scale of 2.25 g (6.48 mmol, 1.00 equiv.). Obtained as a slightly yellow solid in a yield of 78% (3.45 g, 5.05 mmol).

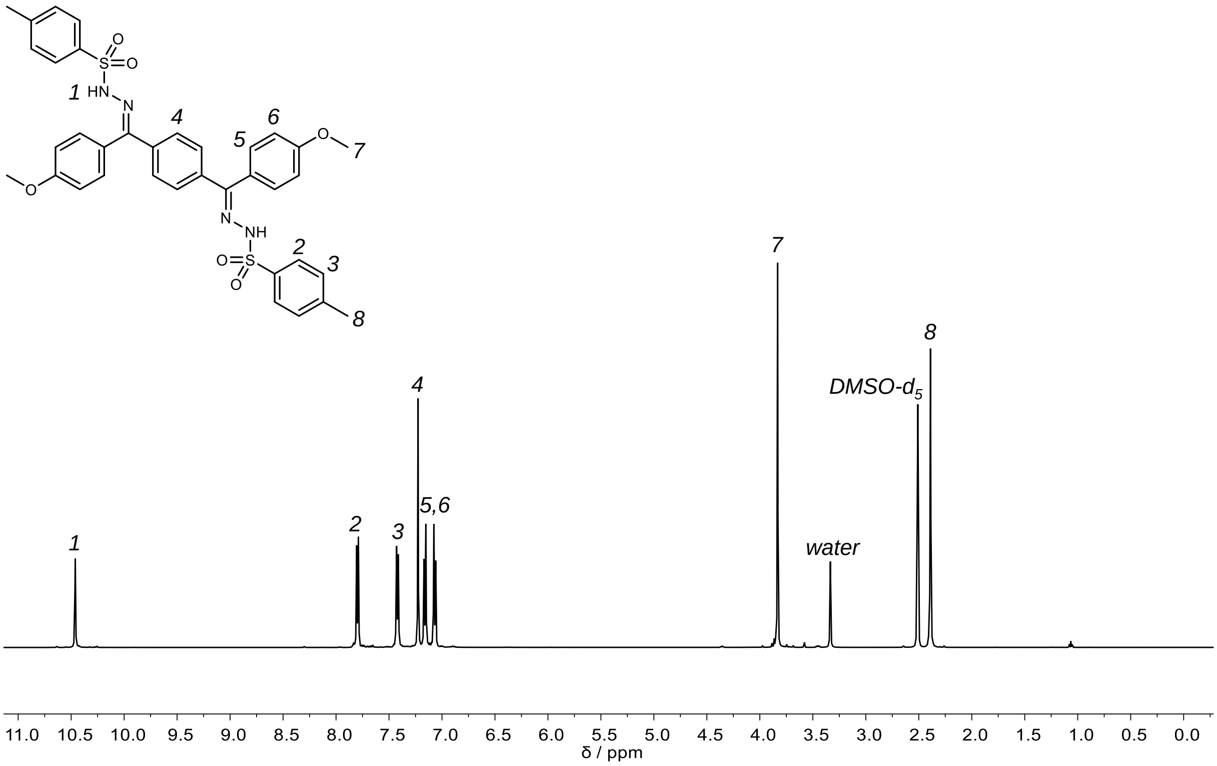

^{1}H NMR (500 MHz, DMSO-*d*$_6$): δ/ppm = 10.46 (s, 2H), 7.83 – 7.74 (m, 4H), 7.50 – 7.37 (m, 4H), 7.23 (s, 4H), 7.20 – 7.11 (m, 4H), 7.09 – 7.02 (m, 4H), 3.83 (s, 6H), 2.39 (s, 6H).

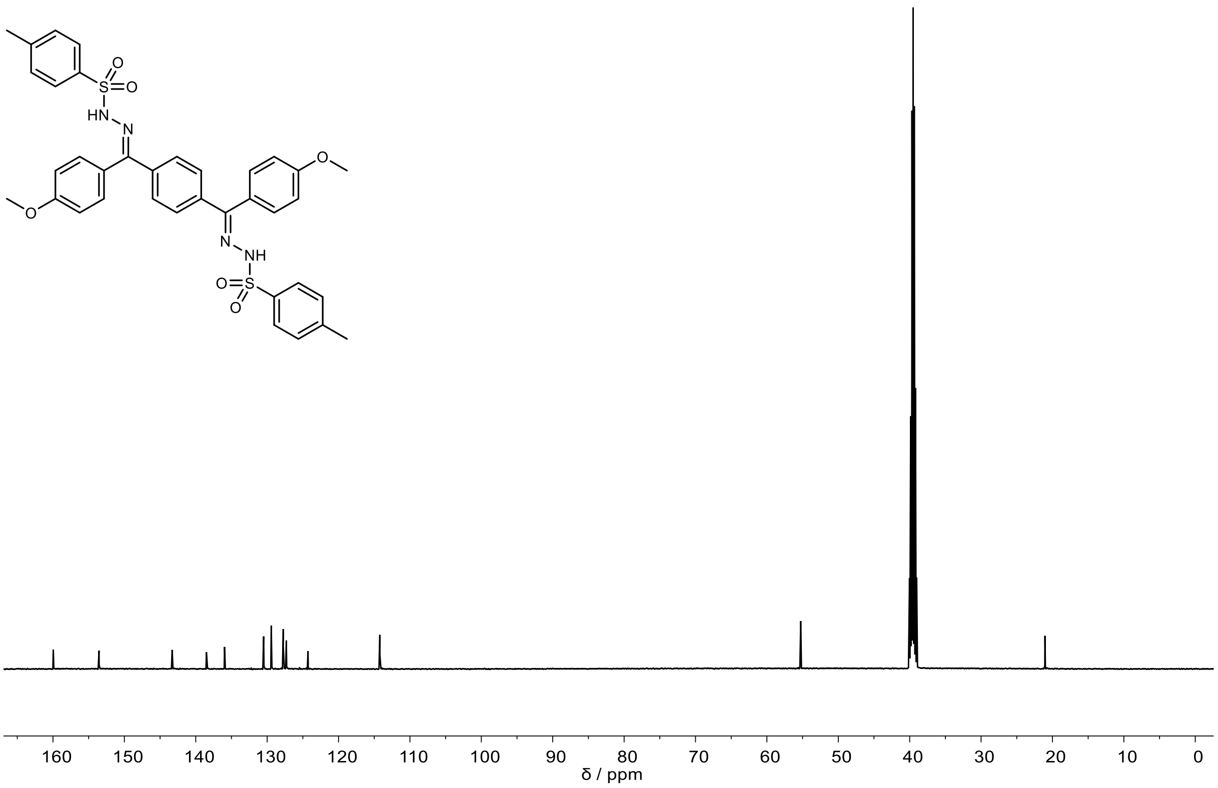

^{13}C NMR (126 MHz, DMSO-*d*6): δ/ppm = 159.96, 153.55, 143.31, 138.50, 135.97, 130.49, 129.43, 127.75, 127.32, 124.27, 114.22, 55.25, 21.05.

IR (ATR platinum diamond): *v*/cm^{-1} = 3178 (w), 1604 (m), 1571 (w), 1512 (w), 1493 (vw), 1440 (vw), 1421 (vw), 1372 (w), 1337 (m), 1316 (w), 1304 (w), 1298 (w), 1281 (w), 1255 (m), 1193 (w), 1185 (w), 1175 (m), 1160 (vs), 1123 (w), 1090 (w), 1045 (w), 1026 (m), 991 (vw), 959 (w), 946 (w), 878 (w), 858 (m), 839 (m), 810 (w), 792 (w), 738 (w), 710 (s), 689 (m), 662 (m), 625 (w), 578 (m), 557 (vs), 545 (s), 525 (m), 500 (vw), 490 (w), 483 (w), 469 (vw).

Synthesis of Terephthalophenone bisNTH (**M2**)

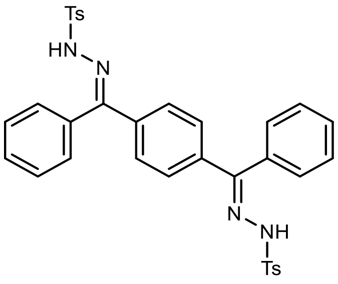

Synthesized from **K2** according to general procedure in a scale of 1.68 g (5.87 mmol, 1.00 equiv.). Obtained as a white solid in a yield of 89% (3.42 g, 5.21 mmol).

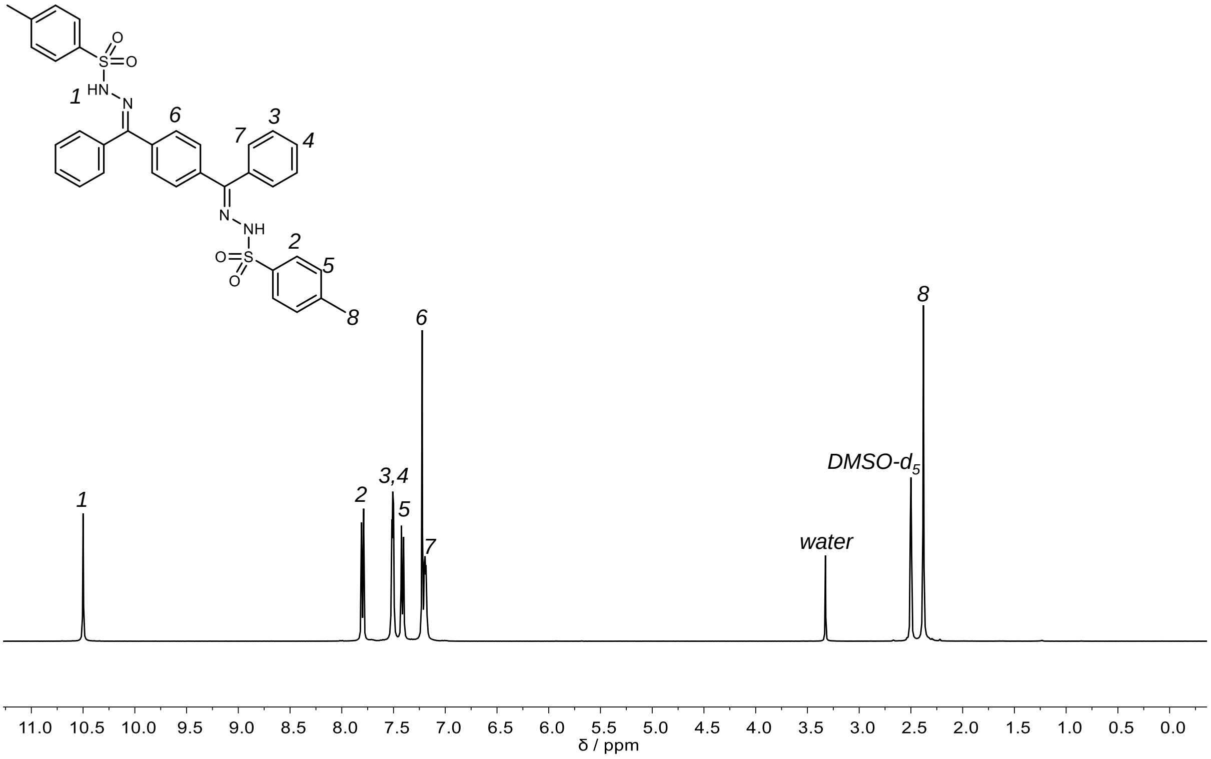

^{1}H NMR (400 MHz, DMSO-d_6): δ 10.50 (s, 2H), 7.88 – 7.71 (m, 4H), 7.59 – 7.47 (m, 6H), 7.47 – 7.36 (m, 4H), 7.22 (s, 4H), 7.21 – 7.17 (m, 4H), 2.38 (s, 6H).

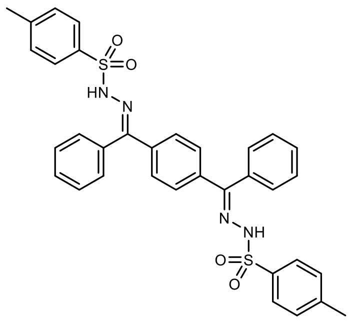

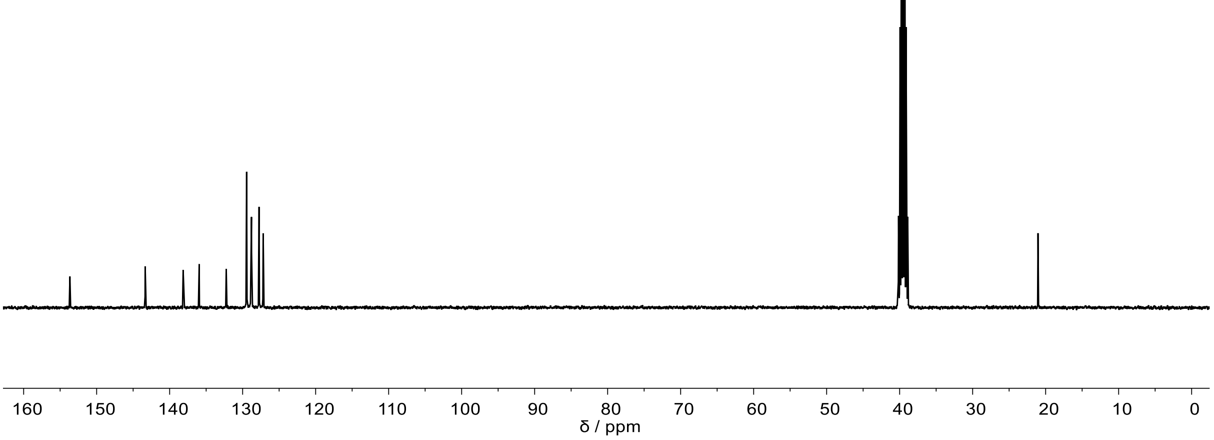

^{13}C NMR (101 MHz, DMSO-*d*$_6$): δ/ppm = 153.66, 143.35, 138.15, 135.95, 132.25, 129.46, 128.85, 128.79, 127.75, 127.19, 21.05.

IR (ATR platinum diamond): *v*/cm^{-1} = 3277 (vw), 3180 (w), 1596 (vw), 1491 (vw), 1444 (vw), 1397 (w), 1378 (m), 1345 (w), 1312 (w), 1183 (m), 1164 (vs), 1117 (vw), 1094 (vw), 1059 (m), 1028 (w), 1020 (w), 998 (vw), 973 (m), 928 (w), 872 (m), 845 (m), 808 (m), 775 (s), 718 (m), 703 (m), 669 (m), 662 (m), 603 (m), 557 (vs), 545 (s), 522 (w), 508 (w), 477 (w), 453 (w).

Synthesis of 4,4'-diphenoxyterephthalophenone bisNTH (**M3**)

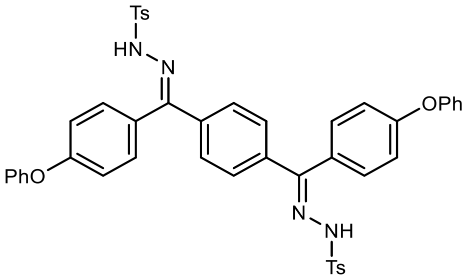

Synthesized from **K3** according to general procedure in a scale of 2.35 g (5.00 mmol, 1.00 equiv.). Obtained as a white solid in a yield of 89% (3.58 g, 4.43 mmol).

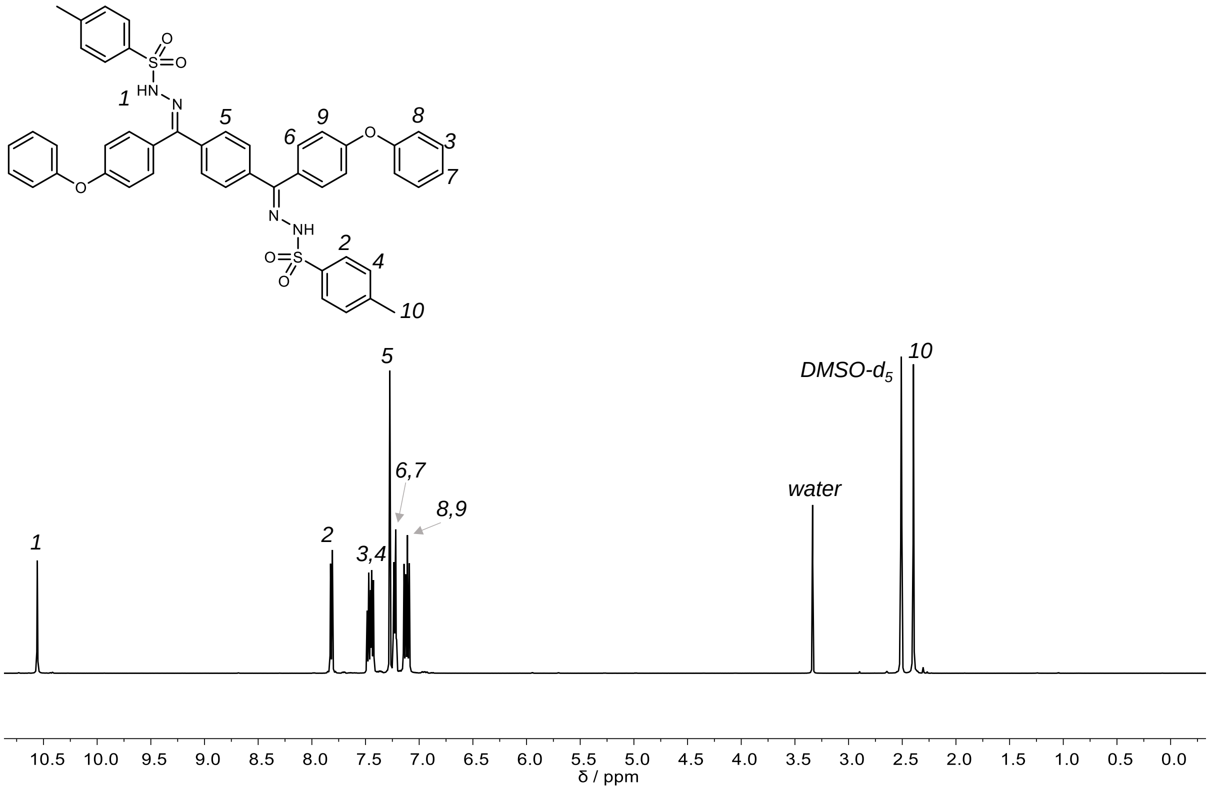

^{1}H NMR (500 MHz, DMSO-d_6): δ/ppm = 10.56 (s, 2H), 7.88 – 7.76 (m, 4H), 7.52 – 7.38 (m, 8H), 7.27 (s, 4H), 7.26 – 7.20 (m, 6H), 7.16 – 7.08 (m, 8H), 2.40 (s, 6H).

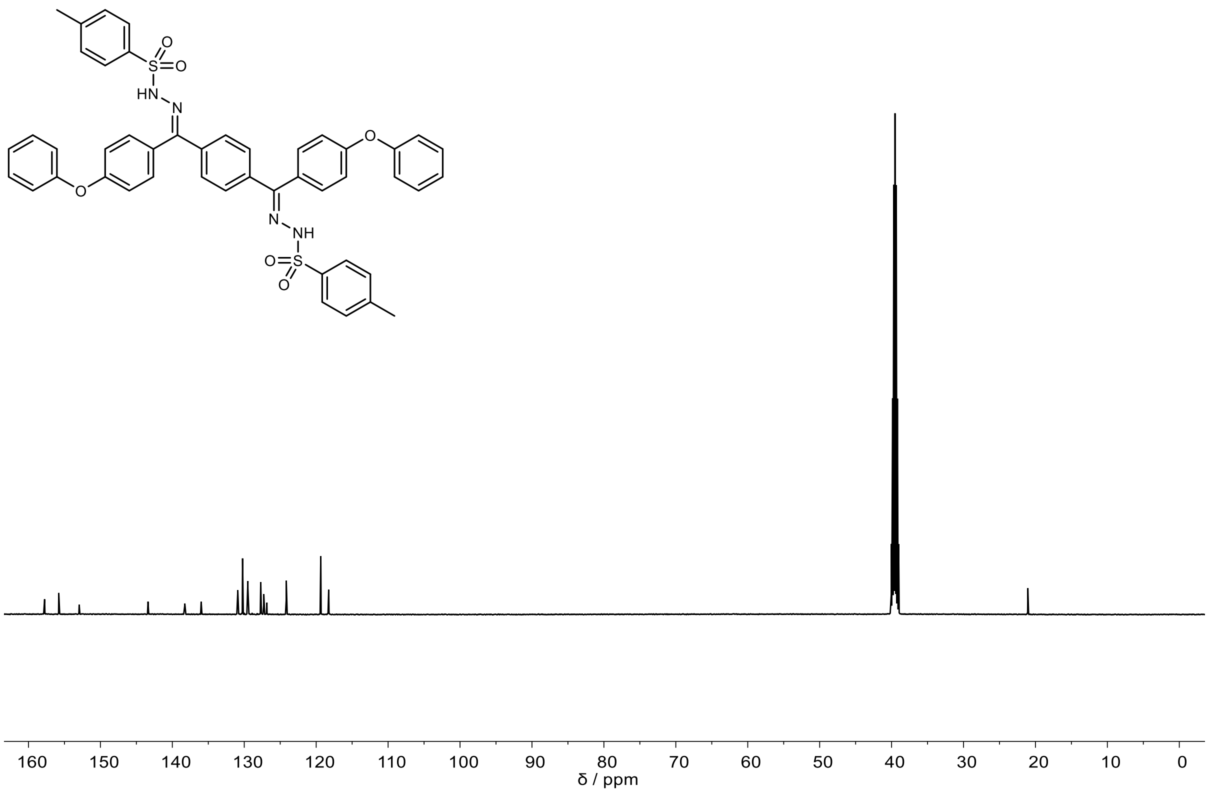

^{13}C NMR (126 MHz, DMSO-*d*$_6$): δ/ppm = 157.76, 155.79, 152.95, 143.38, 138.26, 135.99, 130.90, 130.23, 129.50, 127.71, 127.28, 126.86, 124.16, 119.36, 118.26, 21.06.

IR (ATR platinum diamond): *ν*/cm^{-1} = 3223 (w), 1588 (m), 1500 (w), 1487 (s), 1456 (vw), 1405 (w), 1370 (m), 1349 (s), 1308 (w), 1290 (w), 1230 (vs), 1187 (m), 1166 (vs), 1119 (vw), 1098 (w), 1063 (m), 1014 (w), 977 (m), 963 (vw), 950 (w), 878 (m), 868 (s), 854 (m), 812 (m), 788 (vw), 749 (m), 708 (m), 699 (w), 687 (m), 660 (s), 617 (w), 601 (w), 580 (w), 553 (s), 541 (vs), 508 (w), 496 (m), 479 (m).

Synthesis of 4,4'-bis(phenylthio)terephthalophenone bisNTH (**M4**)

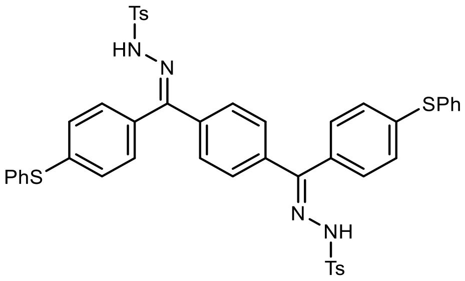

Synthesized from **K4** according to general procedure in a scale of 1.01 g (2.00 mmol, 1.00 equiv.). Obtained as an off-white solid in a yield of 80% (1.35 g, 1.60 mmol).

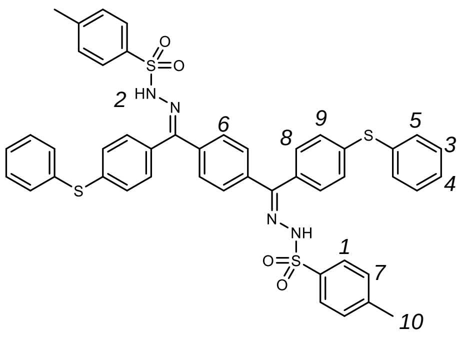

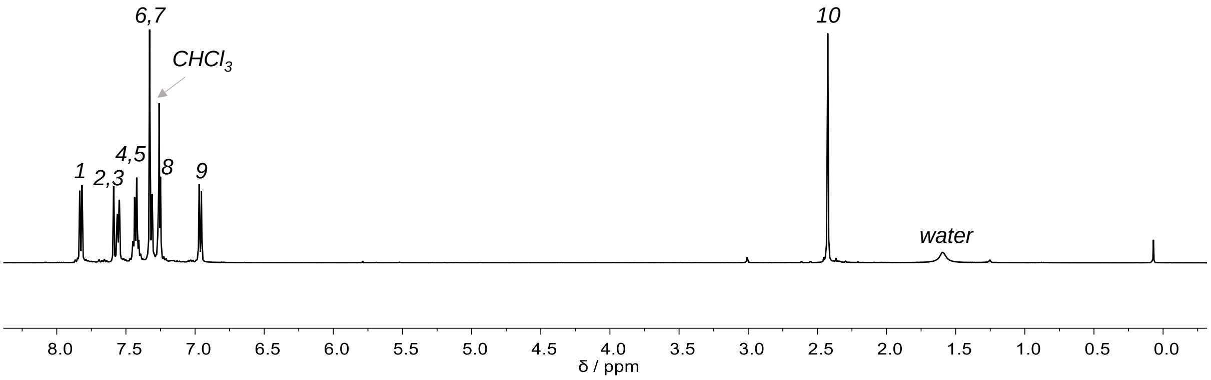

¹H NMR (500 MHz, Chloroform-*d*): δ/ppm 7.85 – 7.80 (m, 4H), 7.60 – 7.58 (m, 2H), 7.57 – 7.53 (m, 4H), 7.46 – 7.39 (m, 7H), 7.35 – 7.30 (m, 8H), 7.28 – 7.23 (m, 7H), 6.99 – 6.92 (m, 4H), 2.42 (s, 6H).

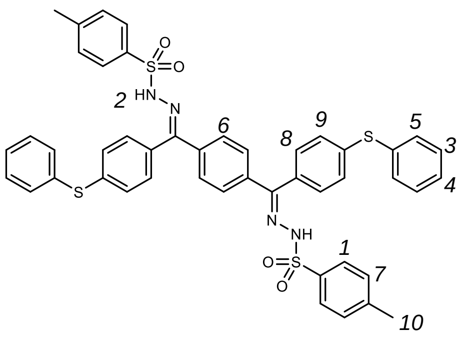

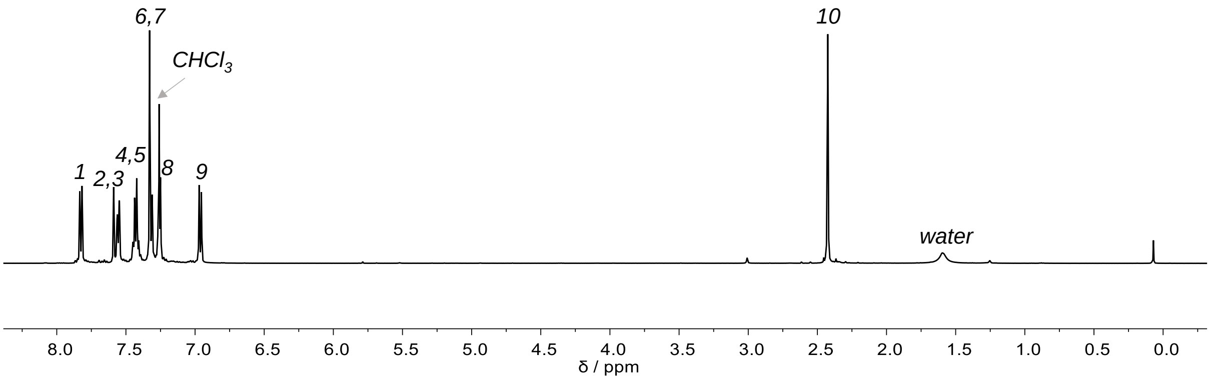

^{13}C NMR (126 MHz, Chloroform-*d*): δ/ppm = 152.81, 144.47, 141.62, 137.74, 135.45, 134.19, 132.12, 129.91, 129.86, 129.04, 128.99, 128.06, 127.85, 127.53, 21.79.

IR (ATR platinum diamond): *v*/cm^{-1} = 3203 (w), 1600 (w), 1489 (w), 1475 (w), 1442 (vw), 1407 (w), 1382 (m), 1345 (m), 1316 (m), 1306 (w), 1290 (w), 1185 (w), 1164 (vs), 1117 (vw), 1109 (vw), 1094 (w), 1082 (w), 1057 (m), 1016 (w), 1000 (vw), 981 (m), 946 (vw), 878 (m), 852 (m), 833 (w), 823 (m), 810 (m), 759 (m), 718 (w), 706 (w), 695 (w), 683 (w), 664 (s), 642 (w), 621 (vw), 555 (vs), 535 (m), 512 (w), 494 (w), 440 (w), 422 (vw).

Synthesis of isophthalophenone bisNTH (**M5**)

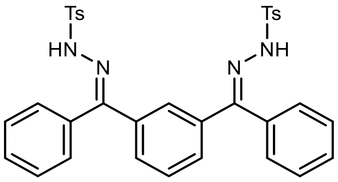

Synthesized from **K5** according to general procedure in a scale of 1.43 g (5.00 mmol, 1.00 equiv.). Obtained as a white solid in a yield of (1.03 g, 1.66 mmol). Purified by column chromatography (cyclohexane / ethyl acetate 5:1).

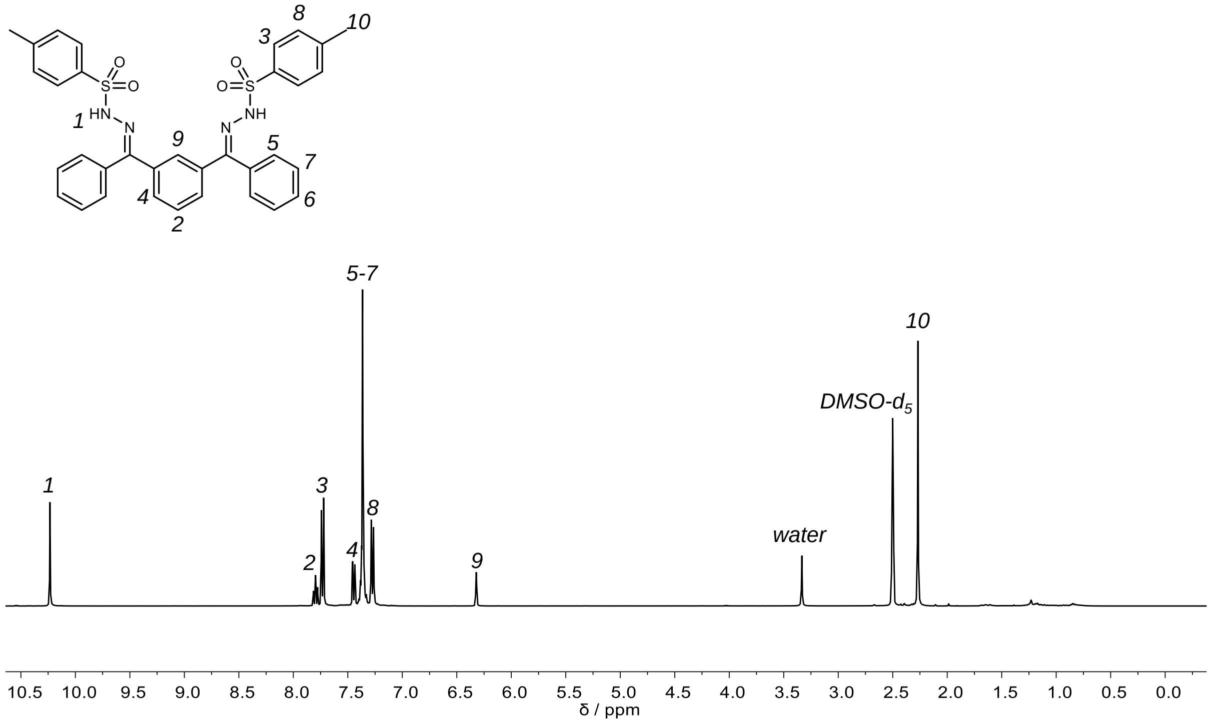

^{1}H NMR (400 MHz, DMSO-d_6): δ/ppm = 10.23 (s, 2H), 7.80 (t, 1H), 7.76 – 7.70 (m, 4H), 7.45 (dd, *J* = 7.7, 1.7 Hz, 2H), 7.37 (t, *J* = 2.0 Hz, 10H), 7.27 (d, *J* = 8.1 Hz, 4H), 6.32 (t, *J* = 1.7 Hz, 1H), 2.27 (s, 6H).

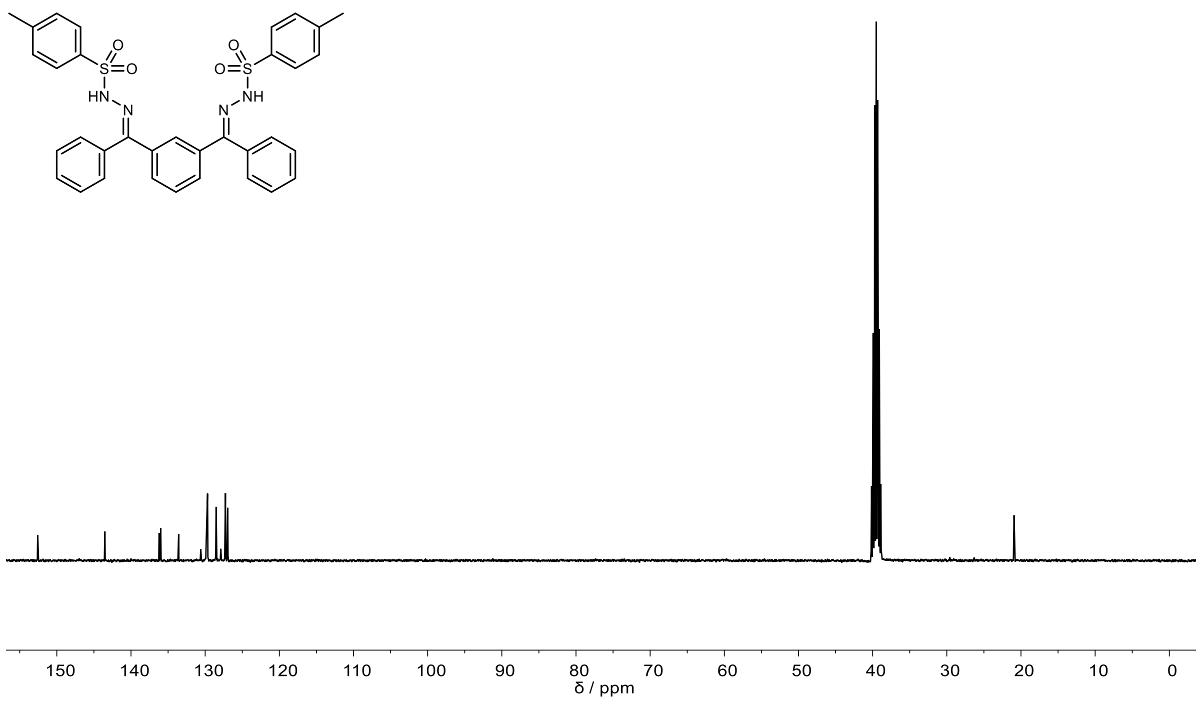

^{13}C NMR (101 MHz, DMSO-d_6): δ/ppm = 152.58, 143.52, 136.21, 135.99, 133.57, 130.59, 129.83, 129.76, 129.68, 128.52, 127.89, 127.27, 126.96, 20.94.

IR (ATR platinum diamond): *v*/cm^{-1} = 3176 (w), 1596 (vw), 1446 (w), 1403 (m), 1347 (m), 1333 (w), 1323 (m), 1308 (w), 1290 (w), 1187 (w), 1166 (s), 1144 (m), 1121 (vw), 1094 (w), 1055 (m), 1028 (w), 1018 (w), 1000 (w), 989 (m), 940 (vw), 924 (w), 899 (w), 878 (vw), 821 (m), 812 (m), 792 (w), 771 (w), 730 (vw), 701 (s), 687 (m), 673 (s), 644 (w), 625 (w), 601 (m), 572 (w), 543 (vs), 500 (w), 483 (m), 428 (w).

Synthesis of 4,4'-bis(benzoyl)biphenyl bis NTH (**M6**)

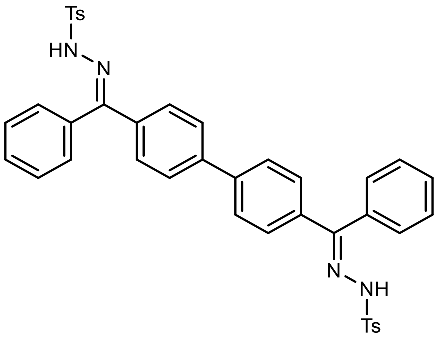

Synthesized from **K6** according to general procedure in a scale 362 mg (1.00 mmol, 1.00 equiv.). Obtained as a white solid in a yield of 65% (452 mg, 0.65 mmol).

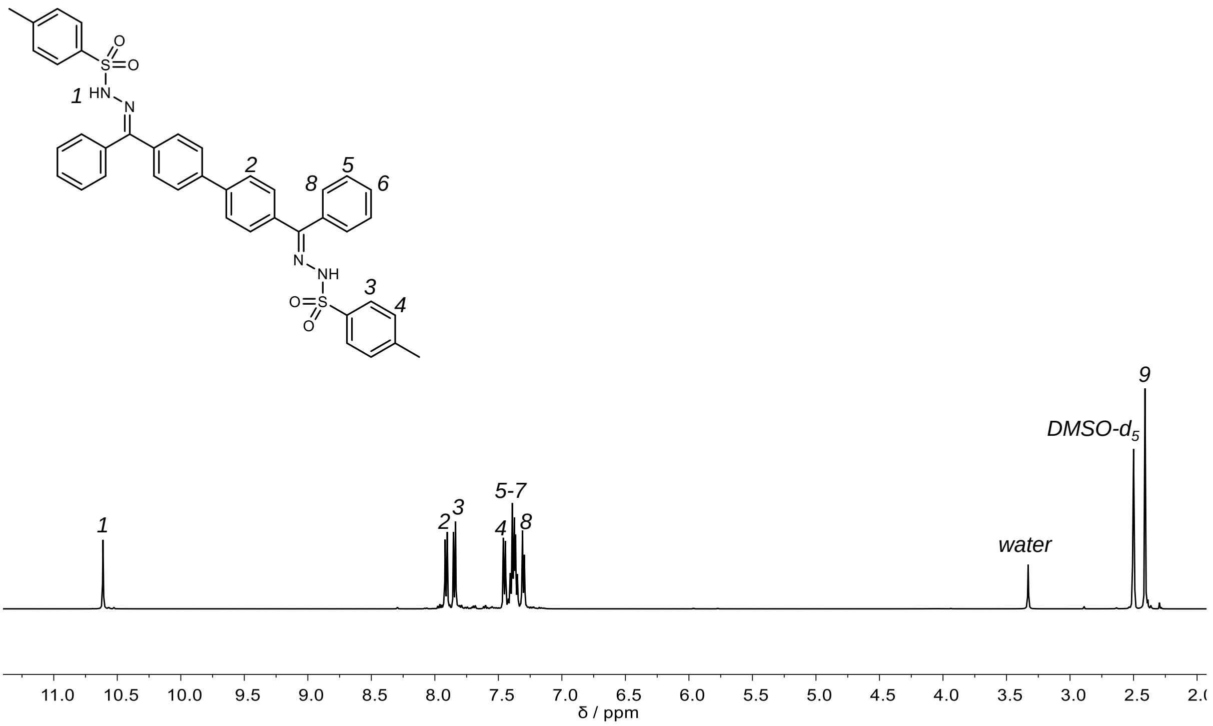

^{1}**H NMR** (500 MHz, DMSO-d_6): δ/ppm = 10.61 (s, 2H), 7.94 – 7.89 (m, 4H), 7.87 – 7.82 (m, 4H), 7.48 – 7.44 (m, 4H), 7.43 – 7.34 (m, 10H), 7.32 – 7.28 (m, 4H), 2.41 (s, 6H).

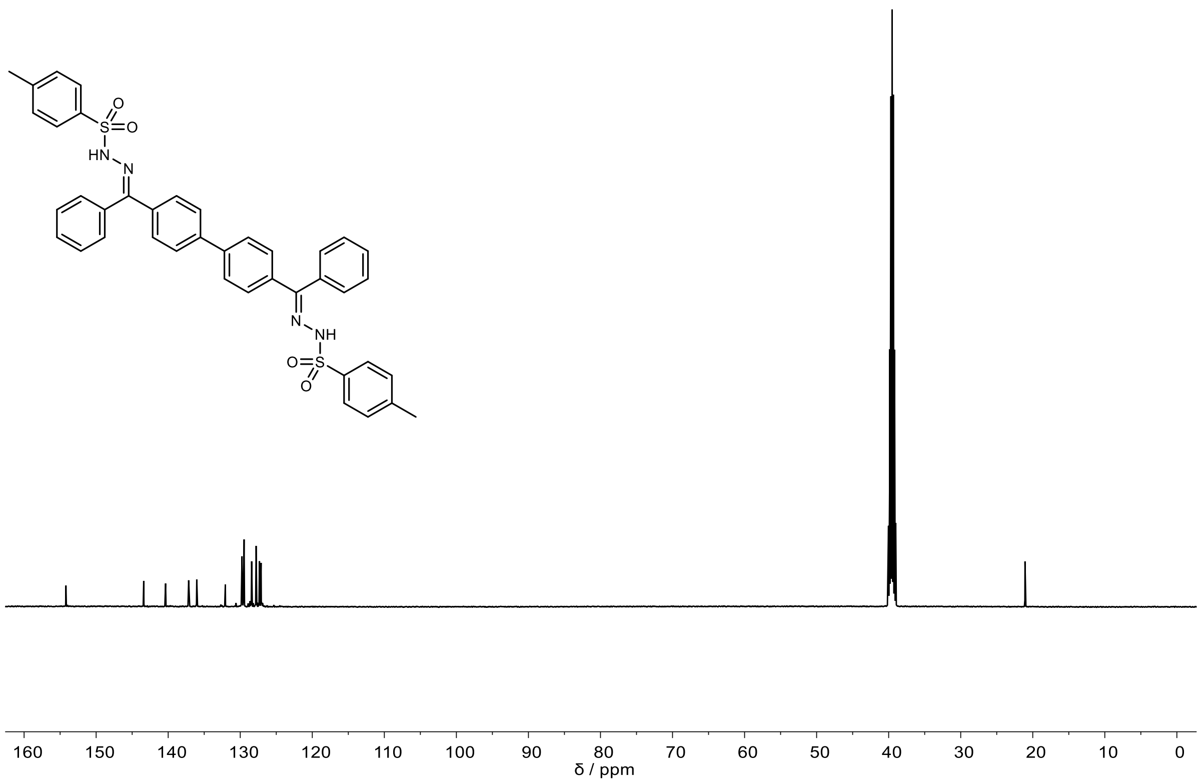

^{13}C NMR (126 MHz, DMSO-*d*$_6$): δ/ppm = 154.19, 143.40, 140.36, 137.16, 136.04, 132.07, 129.78, 129.76, 129.46, 128.42, 127.80, 127.33, 127.11, 21.08.

IR (ATR platinum diamond): *v*/cm^{-1} = 3199 (vw), 1609 (vw), 1598 (w), 1493 (w), 1444 (w), 1401 (w), 1337 (m), 1318 (m), 1304 (w), 1185 (vw), 1160 (vs), 1121 (vw), 1092 (w), 1076 (vw), 1055 (w), 1018 (w), 1004 (vw), 981 (w), 967 (vw), 950 (vw), 876 (m), 823 (m), 806 (w), 778 (m), 747 (vw), 722 (w), 697 (s), 675 (m), 619 (w), 551 (vs), 498 (w), 477 (w).

Synthesis of 2,5-bis(benzoyl)thiophene bisNTH (**M7**)

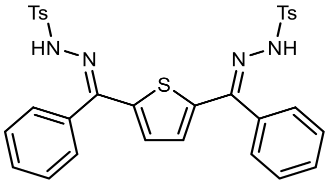

Synthesized from **K7** according to general procedure in a scale of 1.17 g (4.00 mmol, 1.00 equiv.). Obtained as an off-white solid in a yield of 93% (2.35 g, 3.73 mmol).

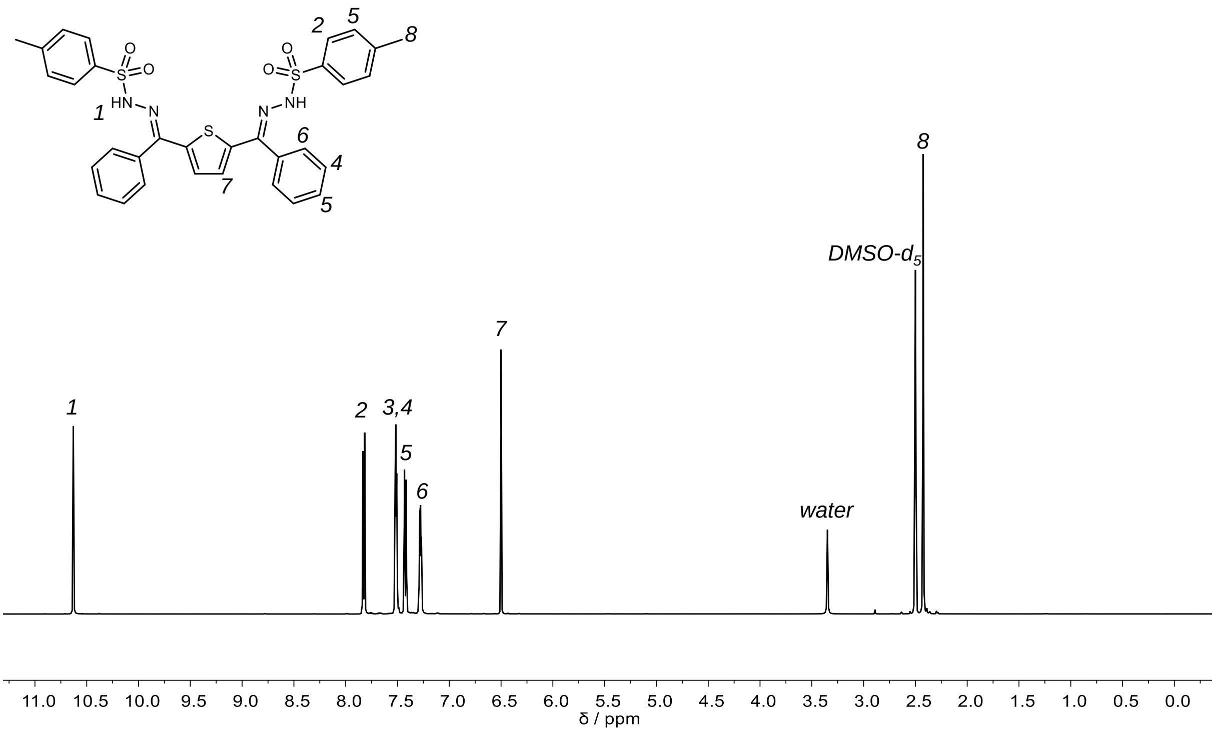

^{1}H NMR (500 MHz, DMSO-d_6): δ/ppm = 10.63 (s, 2H), 7.91 – 7.76 (m, 4H), 7.57 – 7.46 (m, 6H), 7.46 – 7.36 (m, 4H), 7.33 – 7.21 (m, 4H), 6.50 (s, 2H), 2.42 (s, 6H).

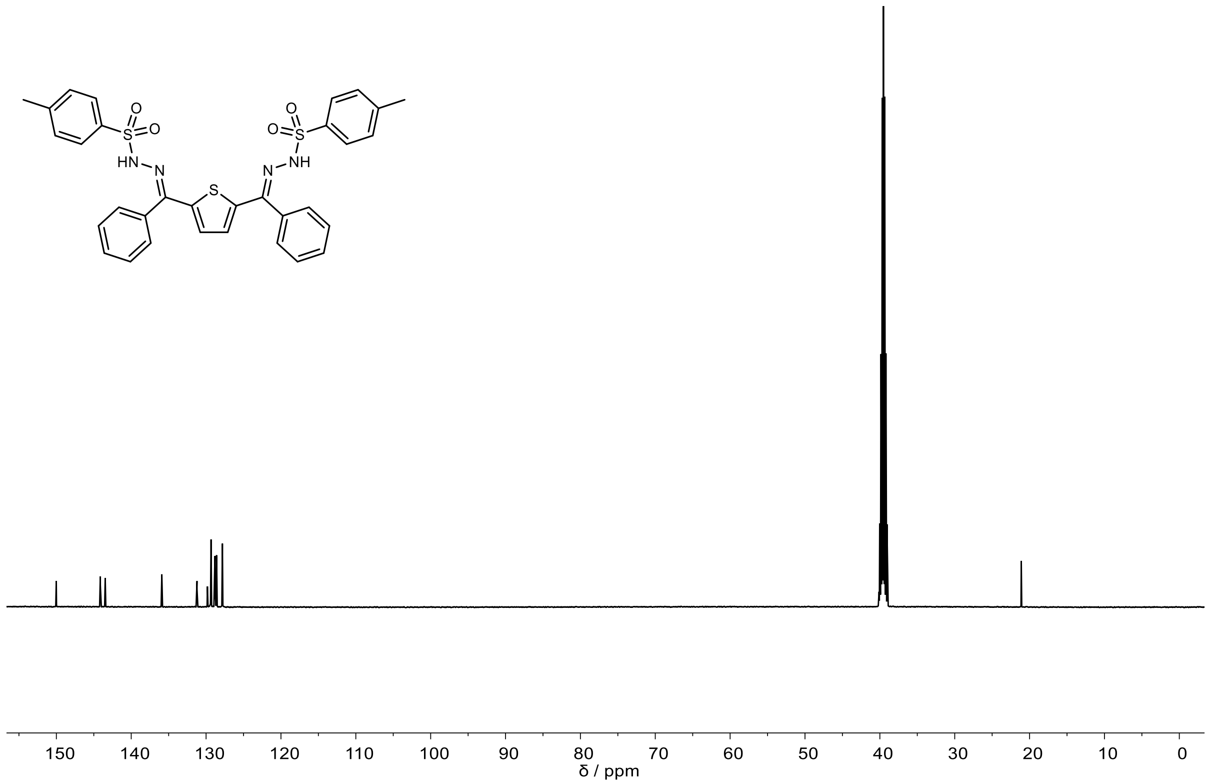

^{13}C NMR (126 MHz, DMSO-d_6): δ/ppm = 150.01, 144.13, 143.47, 135.91, 131.22, 129.82, 129.37, 129.34, 128.82, 128.60, 127.82, 21.11.

IR (ATR platinum diamond): ν/cm^{-1} = 3176 (w), 1596 (w), 1491 (vw), 1444 (w), 1386 (m), 1353 (w), 1343 (m), 1323 (m), 1290 (w), 1187 (w), 1168 (s), 1158 (s), 1121 (vw), 1094 (w), 1068 (m), 1049 (m), 1031 (w), 1018 (w), 1000 (vw), 950 (w), 938 (w), 915 (m), 843 (m), 810 (m), 773 (m), 716 (w), 695 (m), 664 (s), 588 (m), 572 (w), 541 (vs), 518 (m), 494 (w), 469 (w).

*Synthesis of 4,4'-bis(*tert*-butyl)terephthalophenone bisNTH* (**M8**)

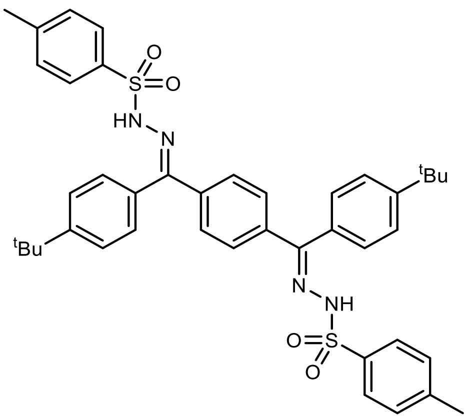

Synthesized from **M8** according to general procedure in a scale of 1.20 g (3.00 mmol, 1.00 equiv.). Obtained as a white solid in a yield of 53% (1.17 mg, 1.60 mmol).

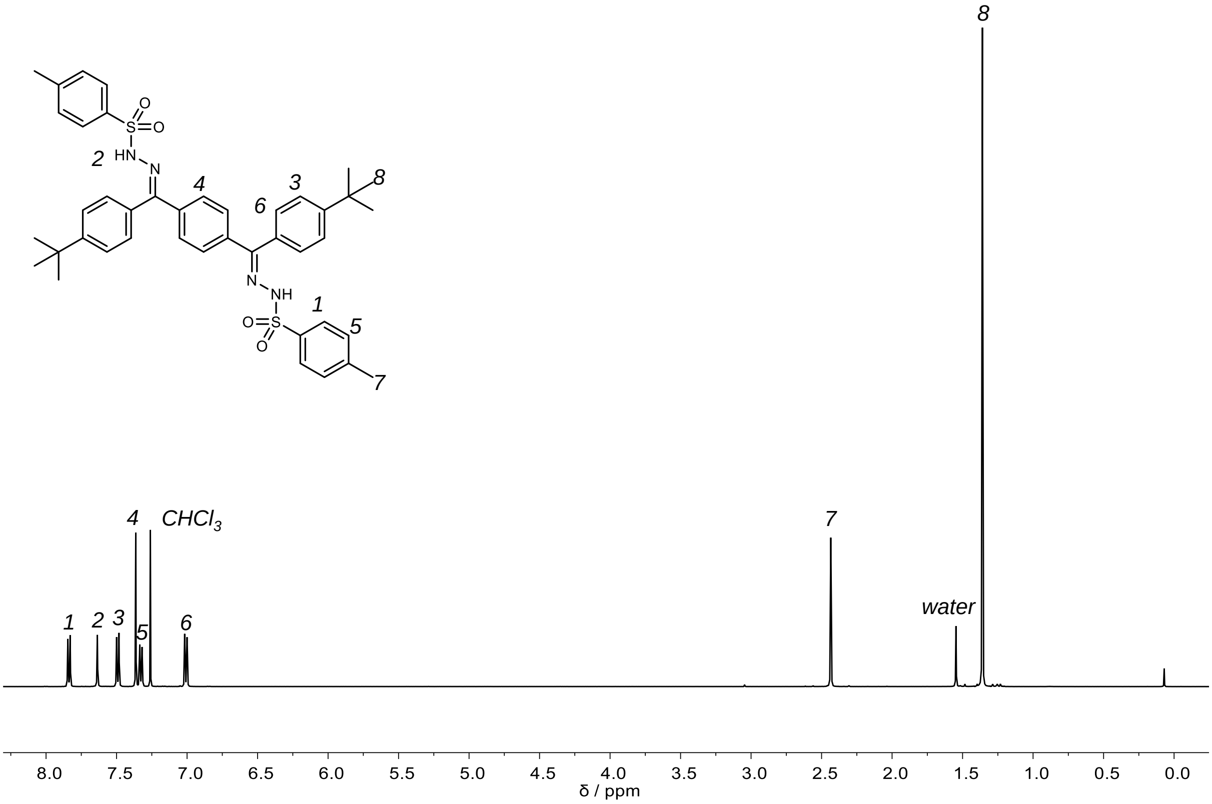

^{1}H NMR (500 MHz, Chloroform-*d*): δ/ppm = 7.86 – 7.81 (m, 4H), 7.64 (s, 2H), 7.52 – 7.47 (m, 4H), 7.36 (s, 4H), 7.35 – 7.31 (m, 4H), 7.04 – 6.97 (m, 4H), 2.43 (s, 6H), 1.36 (s, 18H).

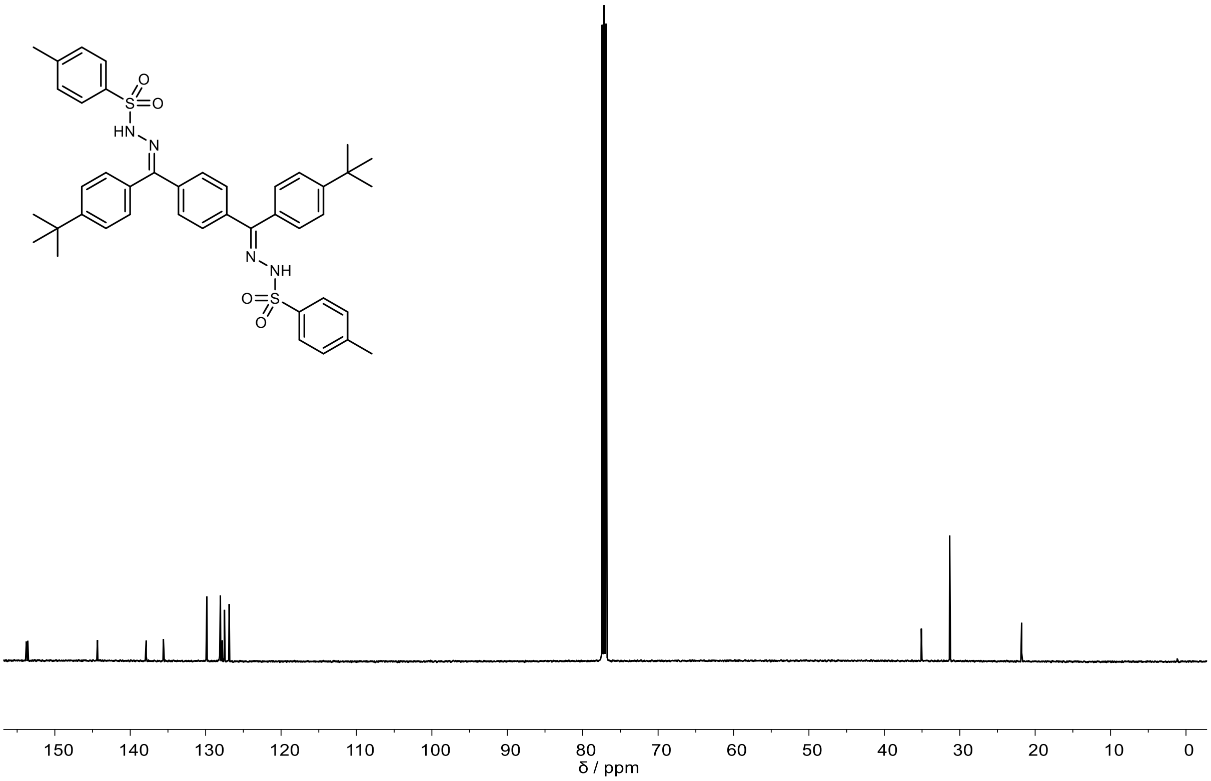

^{13}C NMR (126 MHz, Chloroform-*d*): δ/ppm = 153.77, 153.58, 144.35, 137.88, 135.60, 129.84, 128.11, 128.05, 127.80, 127.51, 126.88, 35.09, 31.33, 21.79.

IR (ATR platinum diamond): *v*/cm^{-1} = 3186 (w), 2966 (vw), 1611 (vw), 1598 (vw), 1497 (vw), 1460 (vw), 1380 (m), 1345 (m), 1310 (w), 1296 (w), 1265 (vw), 1214 (vw), 1189 (w), 1170 (s), 1115 (vw), 1105 (vw), 1094 (w), 1059 (w), 1020 (w), 975 (m), 944 (vw), 876 (w), 854 (w), 841 (w), 831 (w), 812 (w), 761 (m), 753 (m), 728 (w), 706 (w), 679 (m), 652 (w), 636 (w), 617 (w), 570 (s), 553 (vs), 529 (m), 496 (w), 479 (vw), 438 (vw).

3.2 Polymer synthesis

General procedure for the polymerization of bifunctional NTHs

All reagents were dried *in vacuo* before use. All glassware was flame-dried before use. In a Schlenk flask under argon atmosphere, a respective bis(tosylhydrazone) **M**x (0.5 mmol, 1.00 equiv.) was suspended in 1 mL DMSO (c = 0.25 M) and potassium carbonate (2.40 equiv.) and elemental sulfur (0.50 equiv. S_8) were added. The mixture was heated to 70 °C and stirred for 16 hours.
Afterwards, the mixture was cooled to room temperature and diluted with 10 mL of chloroform. After stirring for an additional 5 minutes, the mixture was filtered over celite. The organic phase was washed twice with 10% sodium sulfite solution and thrice with water. After drying over sodium sulfate, the organic phase was concentrated under reduced pressure (V ~ 1mL) and subsequently precipitated into cold MeOH. The precipitated polymer was filtered, washed with MeOH and dried *in vacuo*.

Synthesis of poly-(1,4-phenylene-1,2-bis(methoxyphenyl)vinylene) (**P1**)

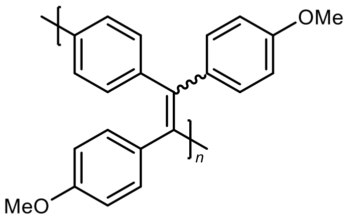

Synthesized from **M1** according to general procedure. Obtained as a yellow solid in a yield of 76% (119 mg, 0.379 mmol).

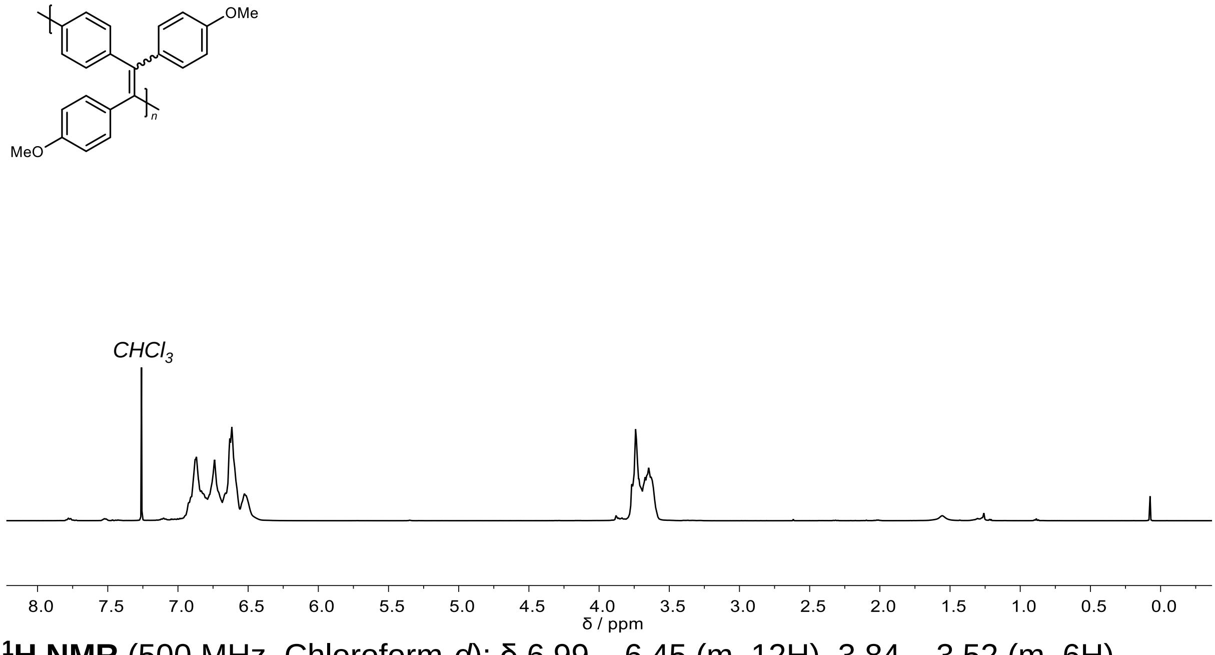

^{1}H NMR (500 MHz, Chloroform-*d*): δ 6.99 – 6.45 (m, 12H), 3.84 – 3.52 (m, 6H).

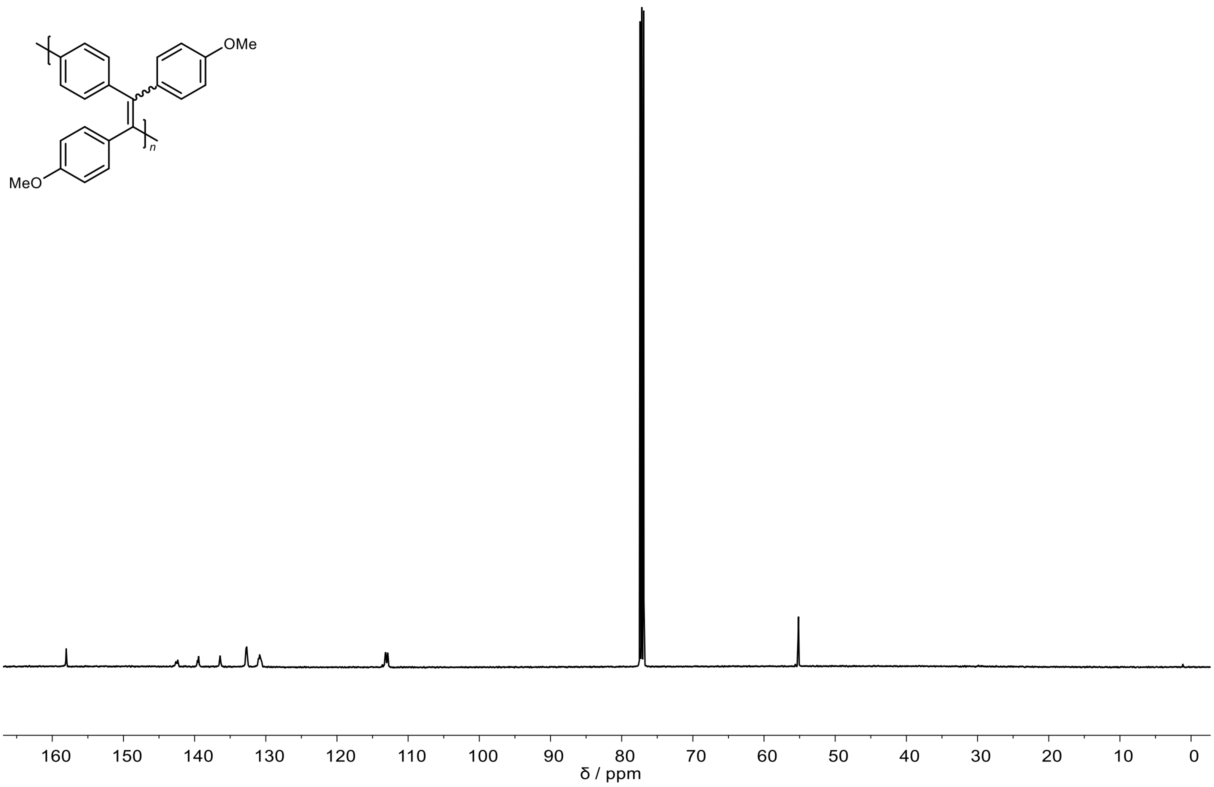

^{13}C NMR (126 MHz, Chloroform-*d*): δ 159.13 – 157.01 (m), 143.29 – 141.35 (m), 140.41 – 138.80 (m), 137.43 – 135.39 (m), 133.70 – 132.41 (m), 131.90 – 129.96 (m), 114.13 – 111.58 (m), 55.80 – 53.79 (m).

IR (ATR platinum diamond): ν/cm^{-1} = 3030 (vw), 2997 (vw), 2950 (vw), 2931 (vw), 2904 (vw), 2832 (vw), 1604 (m), 1574 (w), 1508 (s), 1462 (w), 1440 (w), 1411 (vw), 1401 (vw), 1288 (m), 1242 (vs), 1172 (s), 1140 (vw), 1109 (w), 1033 (m), 975 (vw), 956 (vw), 930 (vw), 860 (vw), 829 (m), 804 (m), 780 (w), 765 (w), 751 (w), 718 (vw), 658 (vw), 625 (vw), 588 (w), 568 (w), 522 (w), 475 (vw).

Elemental Analysis: %**C**, calculated 84.05%, found 81.33%; %**H**, calculated 5.77%, found 5.61%; %**S**, calculated 0.00%, found 0.00 %.

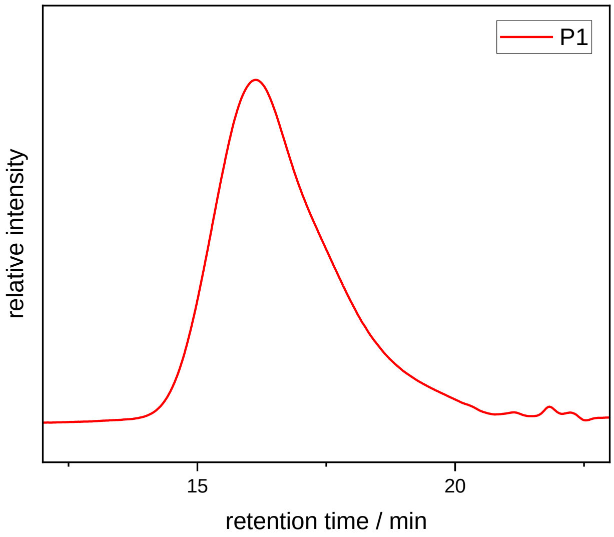

SEC (THF): M_n = 16.2 kDa, M_w = 34.2 kDa, $Đ$ = 2.11.

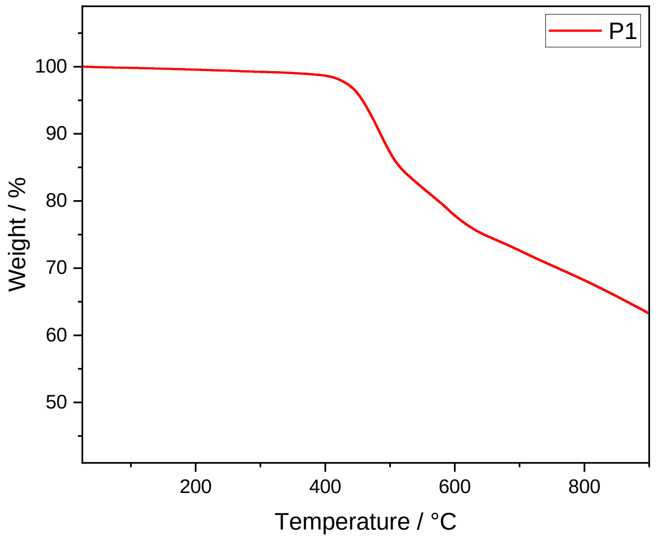

TGA: $T_{d,5\%}$ = 457 °C , Residue = 63.3 %.

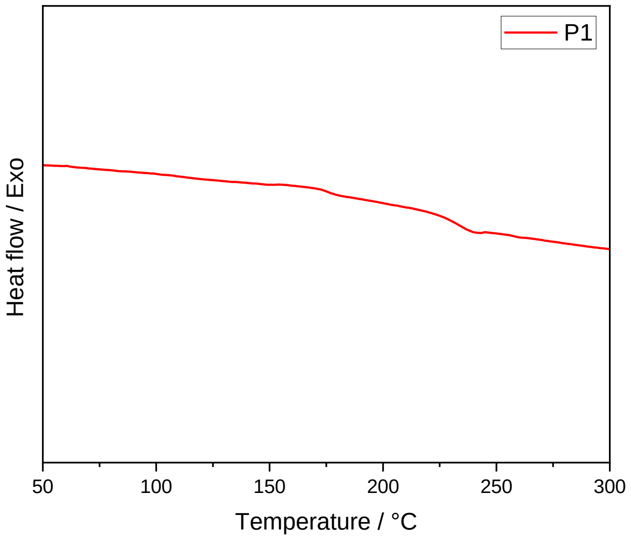

DSC: T_g = 227 °C.

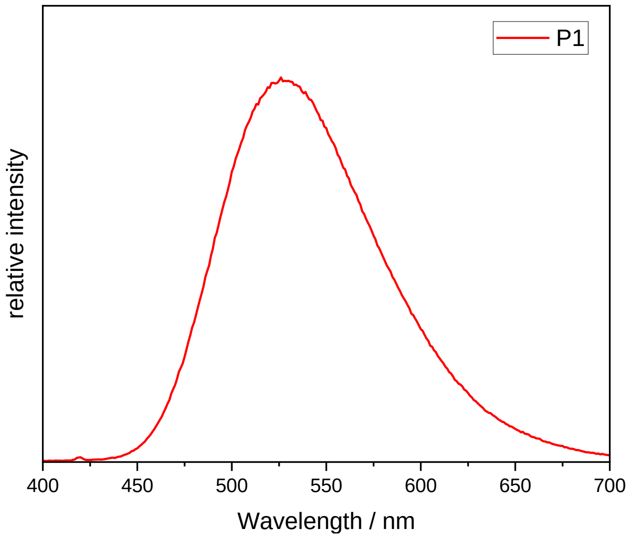

Fluorescence: $\lambda_{em,max}$ = 530 nm with λ_{ex} = 373 nm.

Synthesis of poly-(1,4-phenylene-1,2-diphenylvinylene) (**P2**)

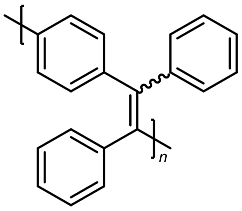

Synthesized from **M2** according to general procedure. Obtained as a yellow solid in a yield of 60% (76.3 mg, 0.300 mmol)

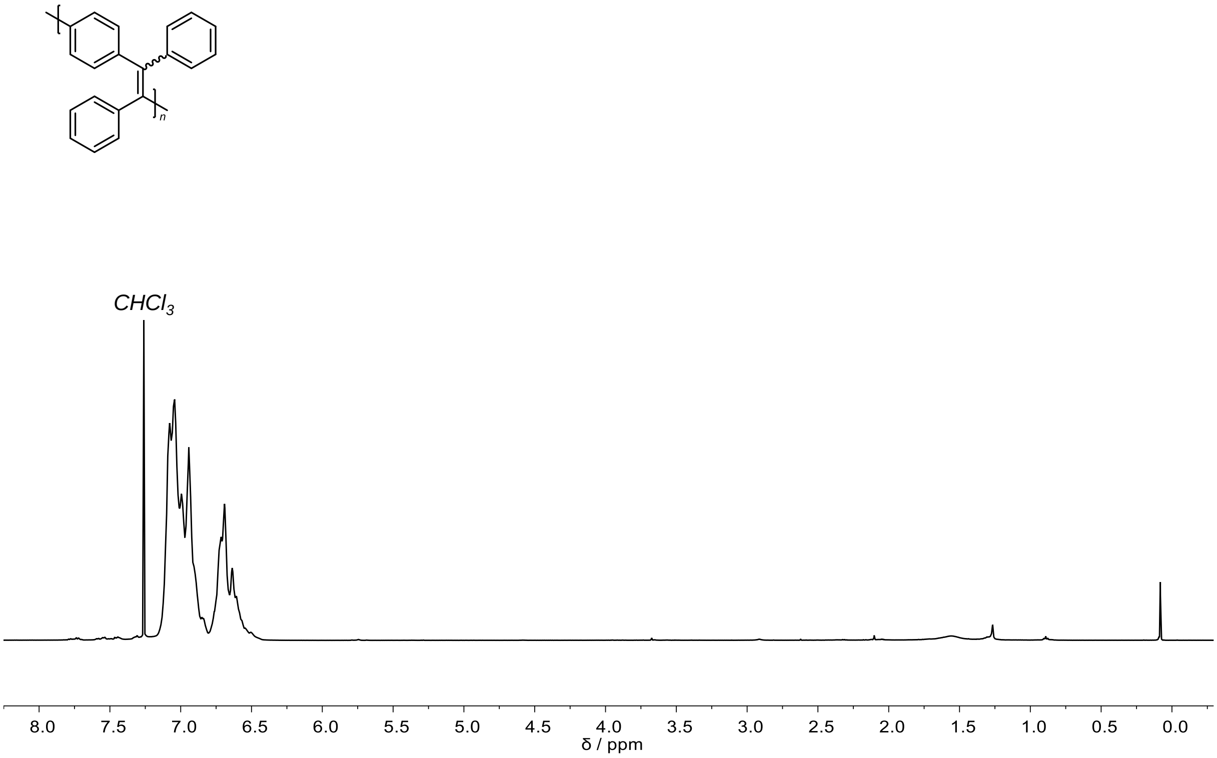

^{1}H NMR (500 MHz, Chloroform-*d*): δ/ppm 7.18 – 6.47 (m, 14H).

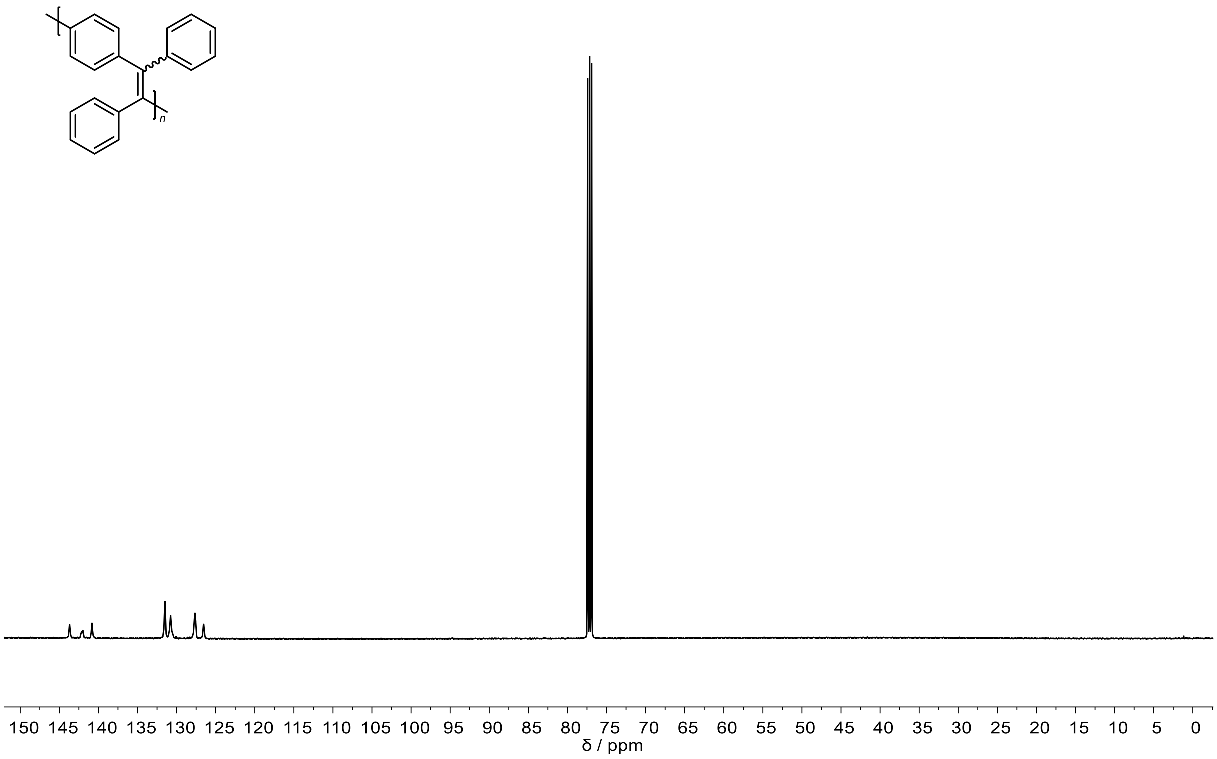

^{13}C NMR (126 MHz, Chloroform-*d*): δ/ppm = 144.02 – 143.29 (m), 142.60 – 141.62 (m), 141.19 – 140.29 (m), 131.86 – 131.17 (m), 131.13 – 130.26 (m), 128.09 – 127.29 (m), 127.04 – 126.06 (m).

IR (ATR platinum diamond): ν/cm^{-1} = 3075 (vw), 3022 (vw), 1596 (vw), 1576 (vw), 1504 (w), 1491 (w), 1442 (w), 1401 (vw), 1275 (vw), 1179 (vw), 1154 (w), 1129 (w), 1111 (w), 1074 (vw), 1028 (w), 1018 (w), 975 (m), 913 (vw), 864 (vw), 852 (vw), 802 (w), 761 (w), 736 (w), 695 (vs), 631 (w), 576 (vw), 492 (w).

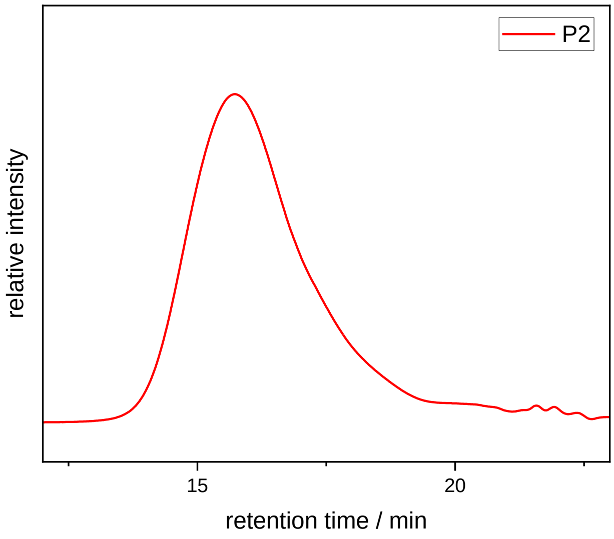

SEC (THF): M_n = 26.9 kDa, M_w = 50.1 kDa, $Đ$ = 1.86.

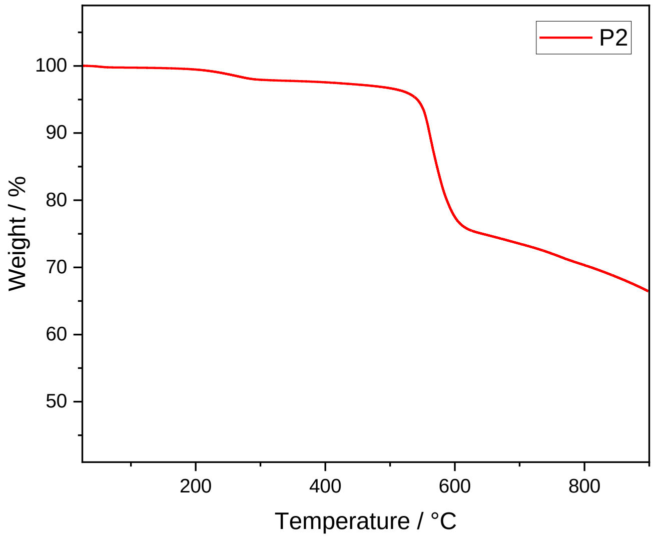

TGA: $T_{d,5\%}$ = 541 °C , Residue = 66.4 %.

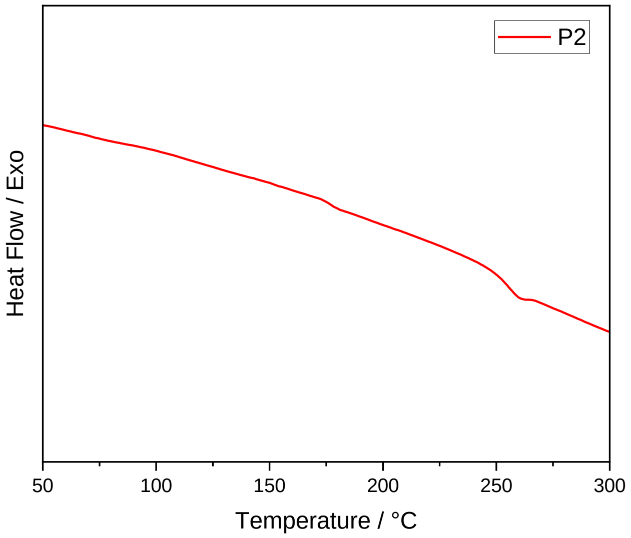

DSC: T_g = 256 °C.

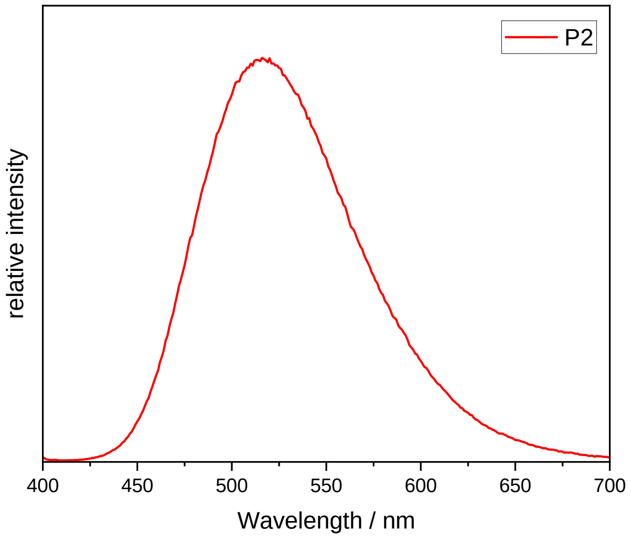

Fluorescence: $\lambda_{em,max}$ = 516 nm with λ_{ex} = 357 nm.

Synthesis of poly-(1,4-phenylene-1,2-bis(4-phenoxyphenyl)vinylene) (**P3**)

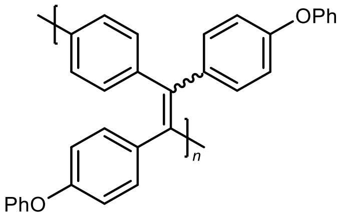

Synthesized from **M3** according to general procedure. Obtained as a yellow solid in a yield of 76% (167 mg, 0.380 mmol).

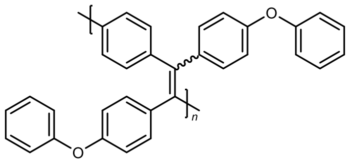

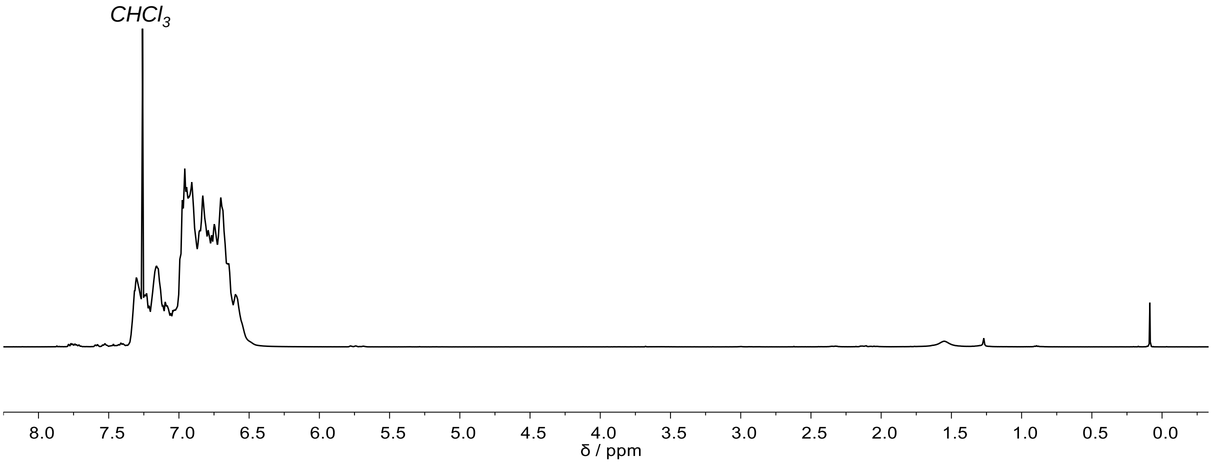

^{1}H NMR (500 MHz, Chloroform-*d*): δ/ppm 7.37 – 6.49 (m, 22H).

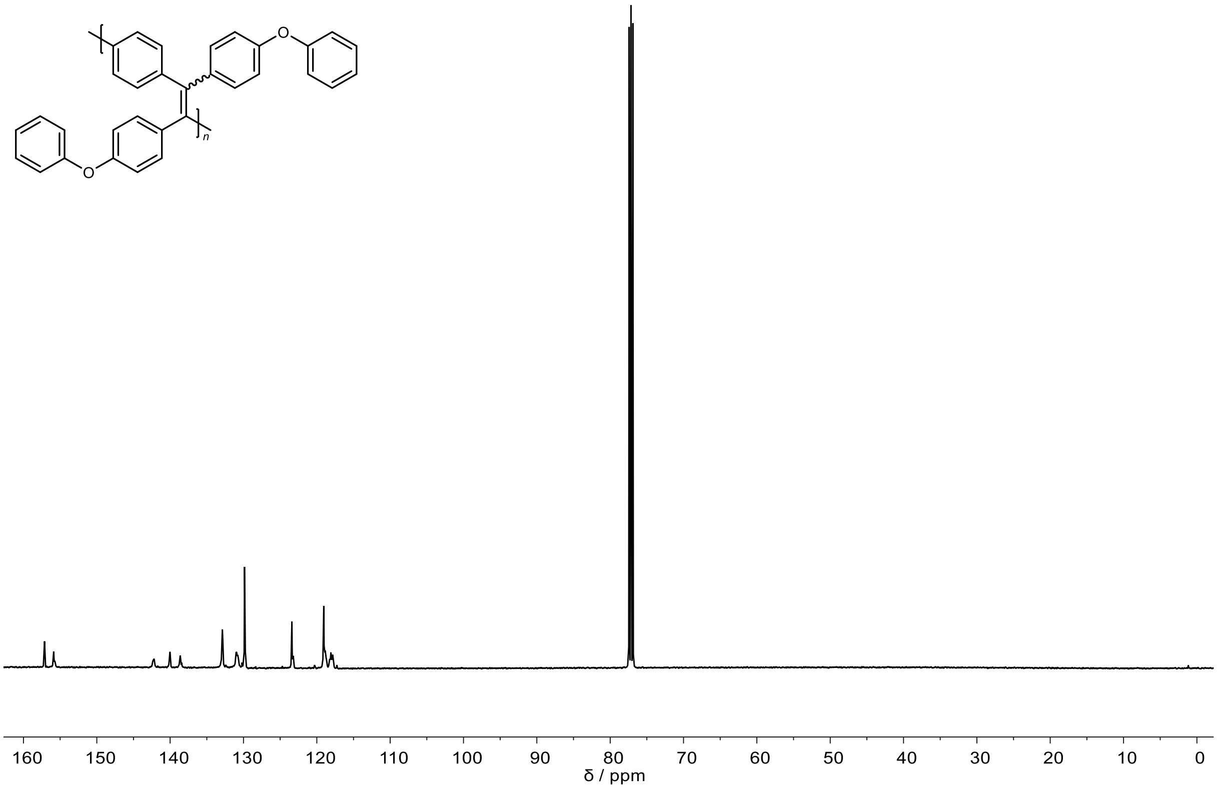

^{13}C NMR (126 MHz, Chloroform-*d*): δ/ppm = 157.42 – 156.70 (m), 156.13 – 155.40 (m), 142.58 – 141.92 (m), 140.34 – 139.76 (m), 138.90 – 138.14 (m), 133.25 – 132.59 (m), 131.29 – 130.42 (m), 130.20 – 129.47 (m), 123.69 – 122.91 (m), 119.47 – 118.62 (m), 118.52 – 117.50 (m).

IR (ATR platinum diamond): ν/cm^{-1} = 3063 (vw), 3055 (vw), 3030 (vw), 1586 (m), 1502 (m), 1485 (vs), 1456 (w), 1405 (vw), 1331 (vw), 1277 (w), 1228 (vs), 1201 (m), 1164 (m), 1107 (w), 1072 (w), 1014 (m), 977 (w), 961 (vw), 907 (vw), 862 (m), 835 (m), 800 (w), 747 (s), 689 (s), 669 (w), 625 (w), 607 (vw), 590 (w), 574 (vw), 539 (vw), 496 (w), 416 (vw), 409 (vw).

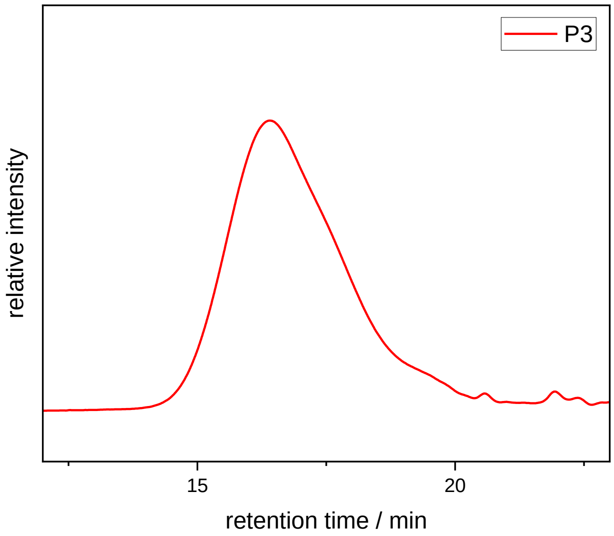

SEC (THF): M_n = 14.7 kDa, M_w = 28.1 kDa, $Đ$ = 1.90.

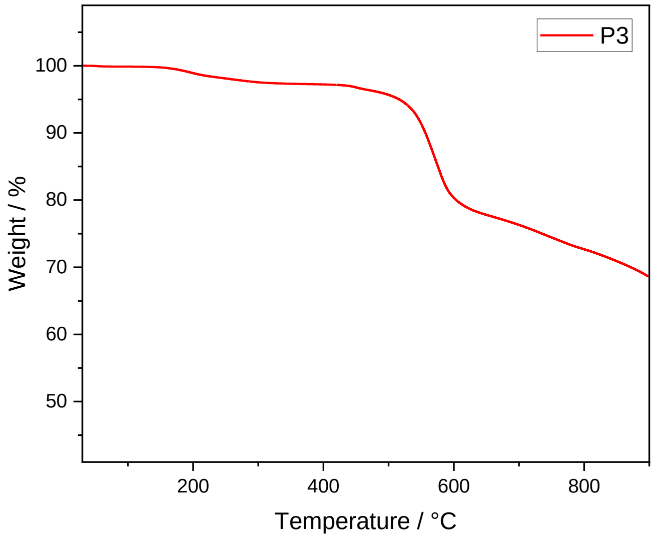

TGA: $T_{d,5\%}$ = 516 °C , Residue = 68.6 %.

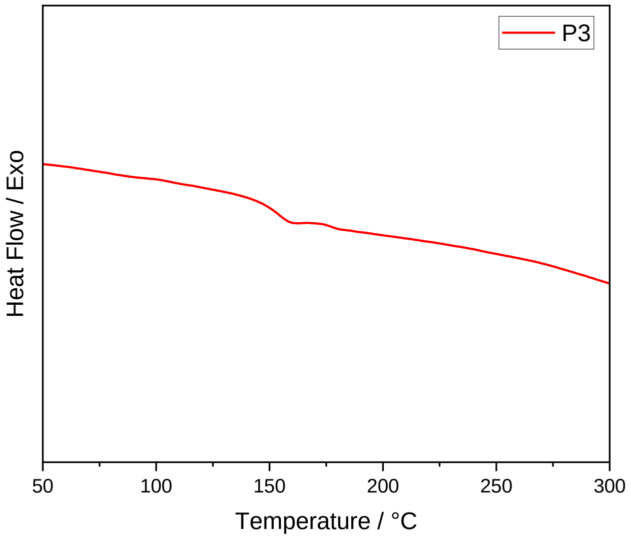

DSC: T_g = 155 °C.

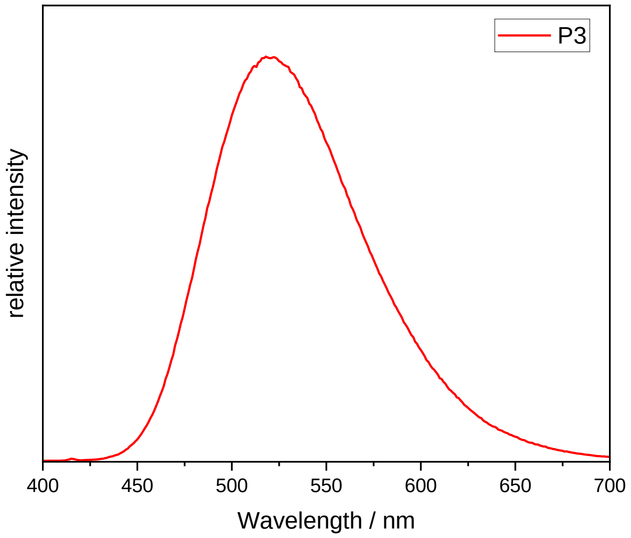

Fluorescence: $\lambda_{em,max}$ = 518 nm with λ_{ex} = 370 nm.

Synthesis of poly-(1,4-phenylene-1,2-bis(4-phenylthiophenyl)vinylene) (**P4**)

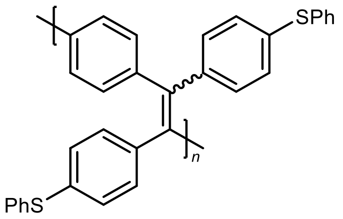

Synthesized from **M4** according to general procedure. Obtained as a yellow solid in a yield of 88% (207 mg, 0.440 mmol).

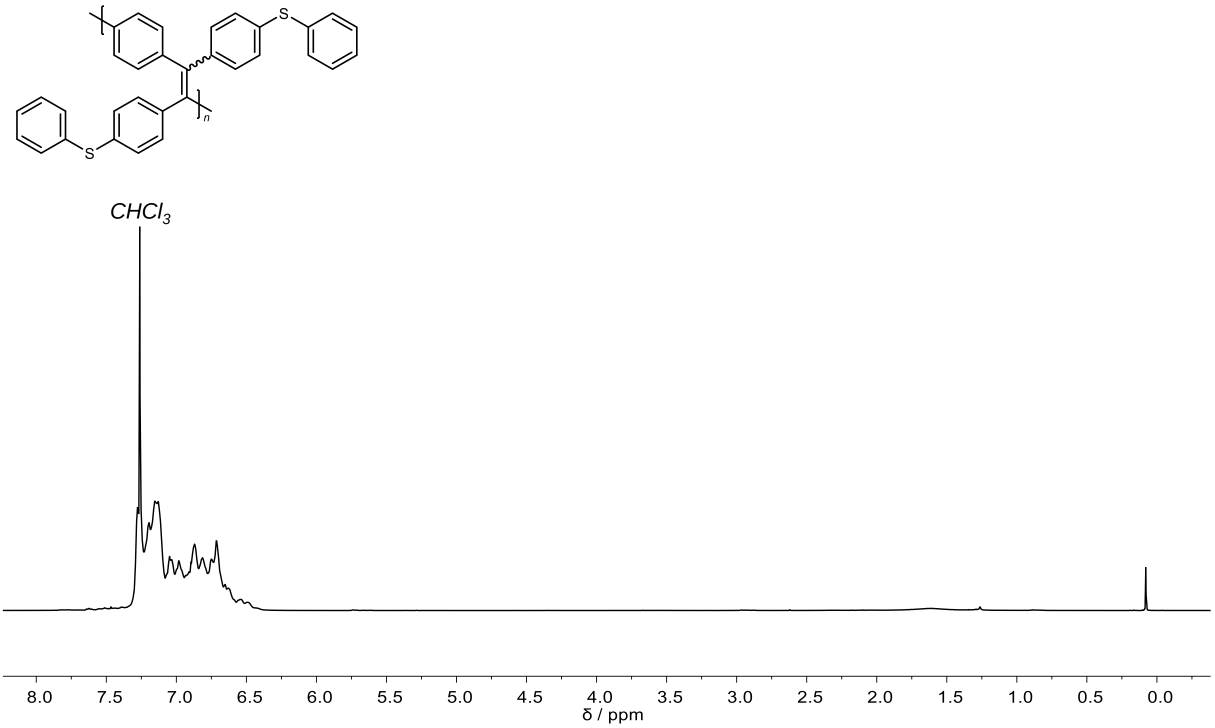

^{1}H NMR (500 MHz, Chloroform-*d*): δ/ppm = 7.37 – 6.45 (m, 22H).

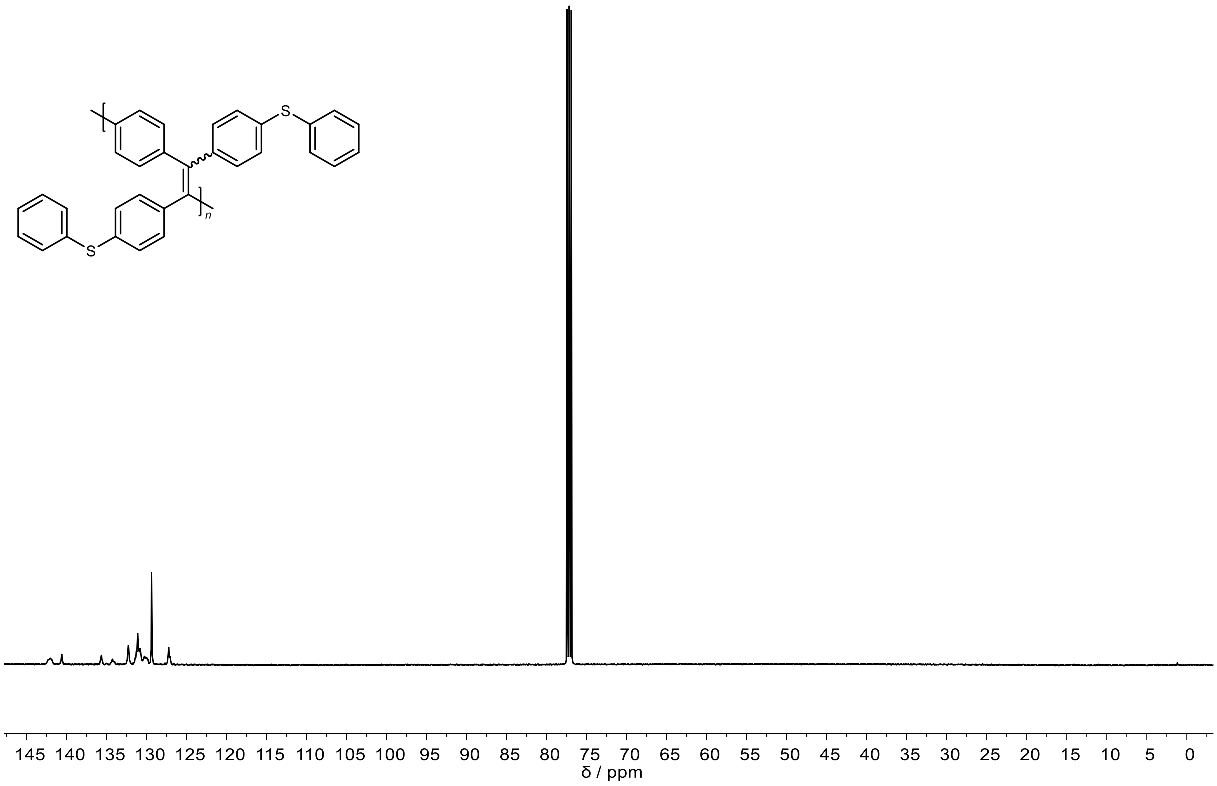

^{13}**C NMR** (126 MHz, Chloroform-*d*): δ/ppm = 142.47 – 141.67 (m), 140.89 – 140.35 (m), 136.00 – 135.34 (m), 134.58 – 133.82 (m), 132.65 – 131.99 (m), 131.79 – 129.76 (m), 129.65 – 129.04 (m), 127.45 – 126.79 (m).

IR (ATR platinum diamond): ν/cm^{-1} = 3055 (vw), 3048 (vw), 3020 (vw), 1580 (m), 1551 (vw), 1500 (w), 1487 (m), 1475 (s), 1438 (m), 1395 (w), 1302 (vw), 1269 (w), 1179 (vw), 1135 (w), 1109 (w), 1080 (m), 1014 (m), 1000 (m), 975 (w), 926 (vw), 870 (w), 858 (w), 825 (m), 790 (m), 734 (vs), 687 (vs), 627 (w), 578 (w), 568 (w), 525 (w), 514 (w), 467 (m), 438 (vw), 432 (vw), 424 (vw).

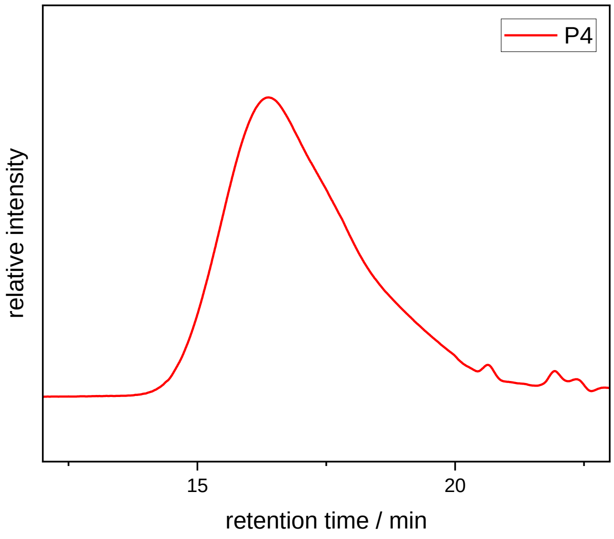

SEC (THF): M_n = 12.5 kDa, M_w = 27.8 kDa, $Đ$ = 2.23.

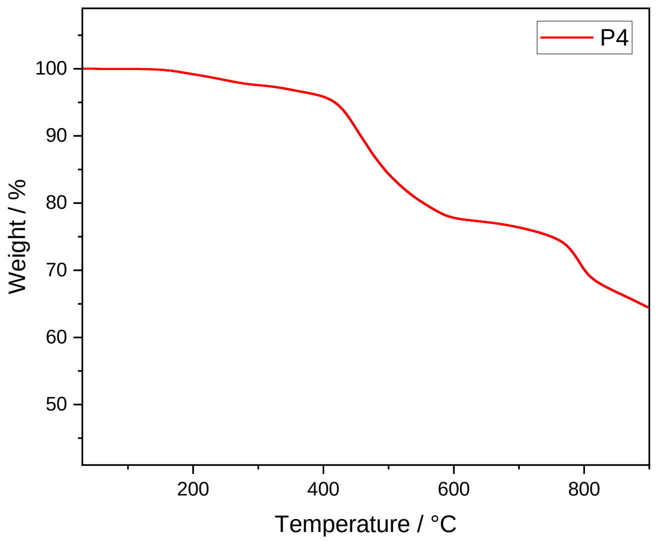

TGA: $T_{d,5\%}$ = 471 °C , Residue = 64.4 %.

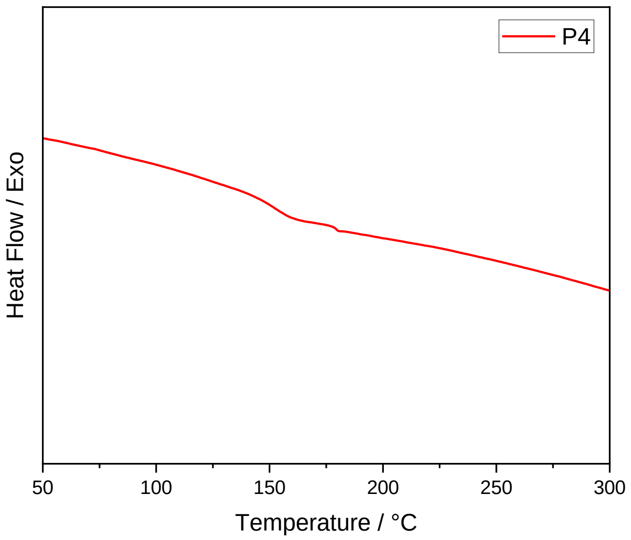

DSC: T_g = 161 °C.

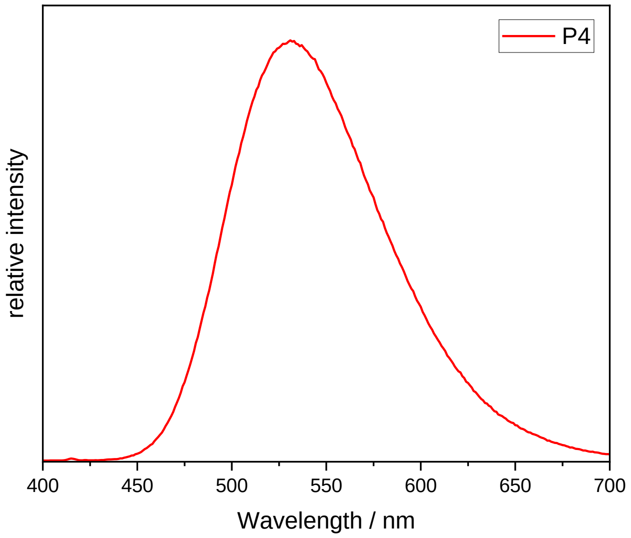

Fluorescence: $\lambda_{em,max}$ = 531 nm with λ_{ex} = 370 nm.

Synthesis of poly-(1,3-phenylene-1,2-diphenylvinylene) (**P5**)

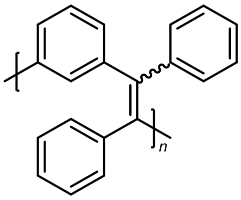

Synthesized from **M5** according to general procedure. Obtained as an off-white solid in a yield of 47% (60.2 mg, 0.237 mmol).

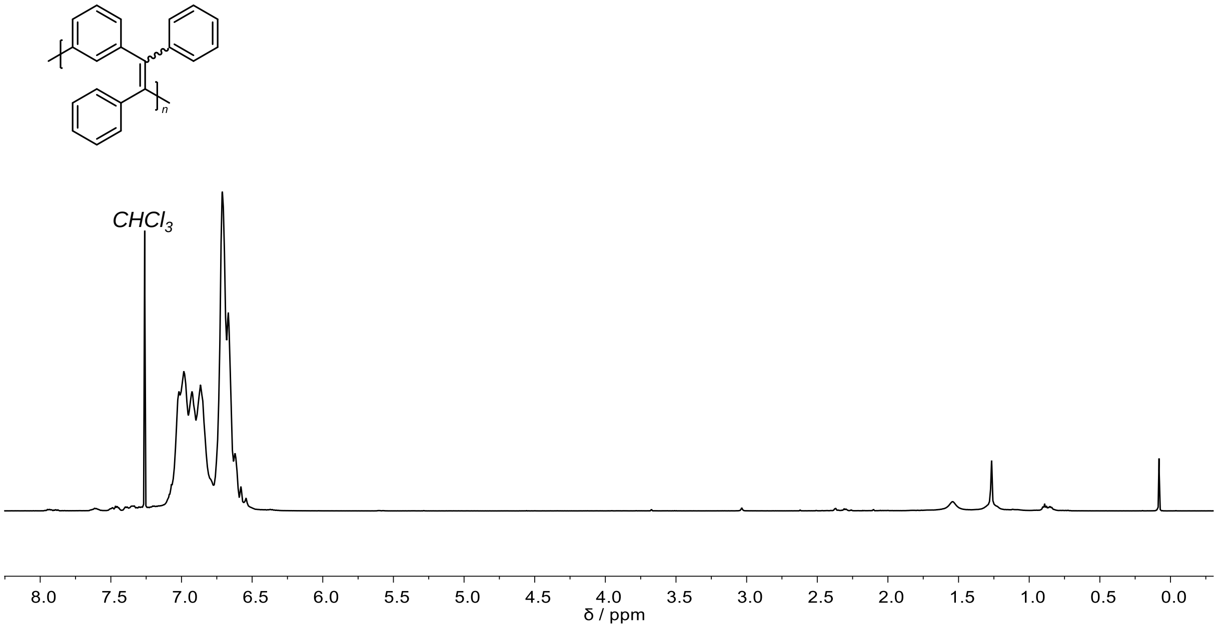

^{1}H NMR (500 MHz, Chloroform-*d*): δ/ppm = 7.12 – 6.53 (m, 14H).

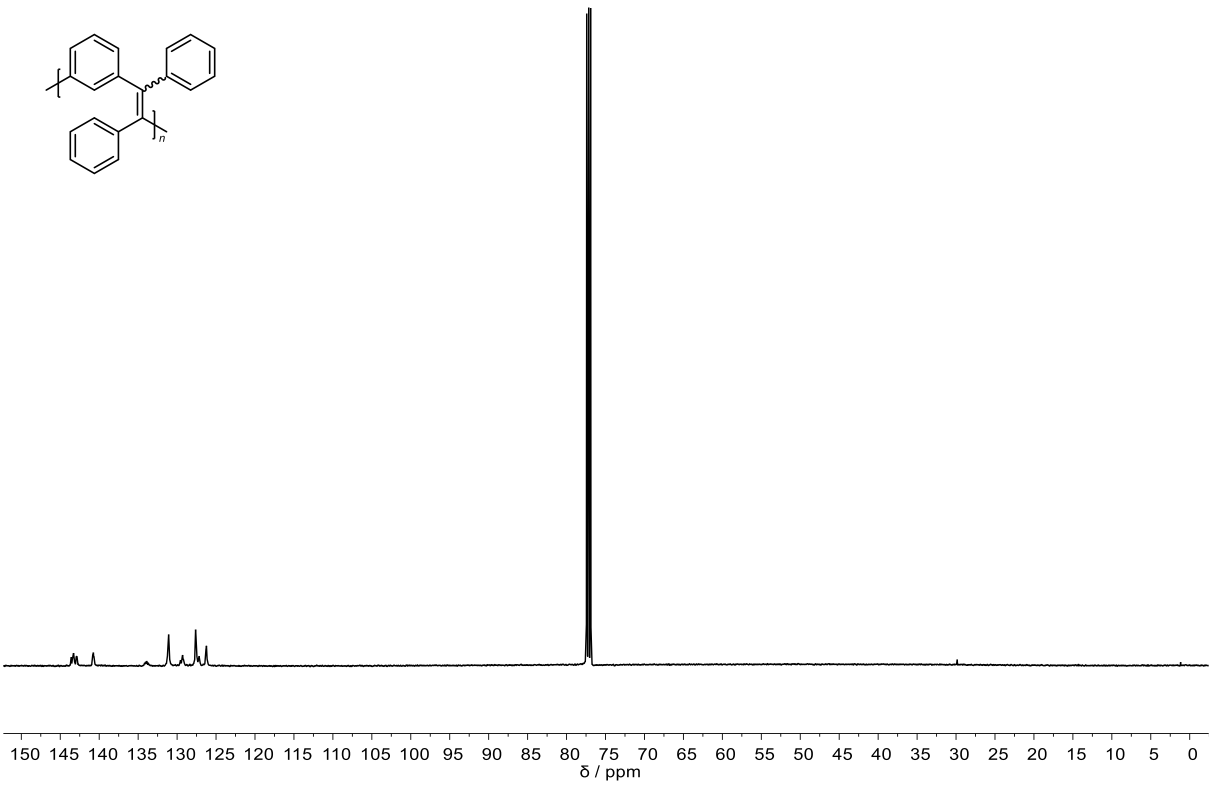

^{13}C NMR (126 MHz, Chloroform-*d*): δ/ppm = 143.82 – 142.61 (m), 141.15 – 140.43 (m), 134.28 – 133.48 (m), 131.39 – 130.74 (m), 129.82 – 128.81 (m), 127.92 – 126.91 (m), 126.52 – 125.86 (m).

IR (ATR platinum diamond): ν/cm^{-1} = 3077 (vw), 3052 (vw), 3020 (vw), 1596 (w), 1576 (vw), 1491 (w), 1442 (w), 1179 (vw), 1156 (vw), 1086 (vw), 1074 (w), 1031 (vw), 1000 (vw), 911 (vw), 788 (w), 761 (w), 695 (vs), 671 (w), 640 (vw), 619 (w), 564 (w), 487 (vw), 463 (vw), 453 (vw), 440 (vw), 434 (vw).

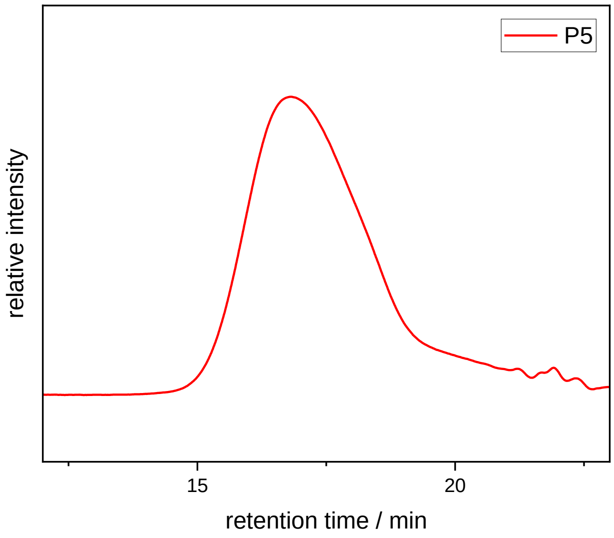

SEC (THF): M_n = 10.3 kDa, M_w = 20.7 kDa, $Đ$ = 2.01.

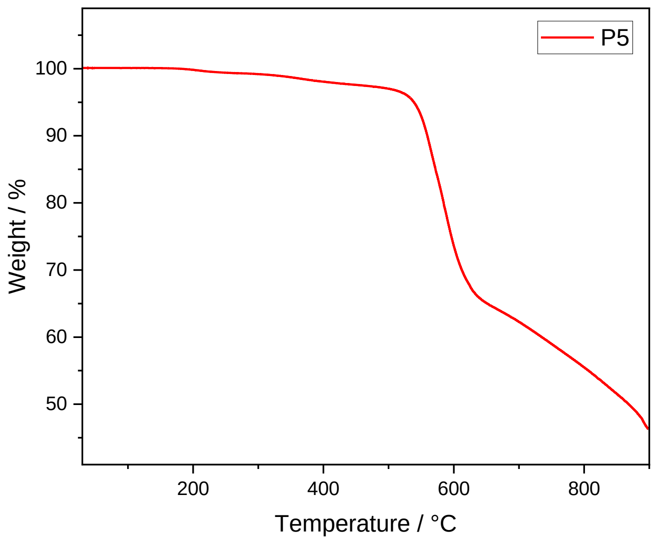

TGA: $T_{d,5\%}$ = 538 °C , Residue = 46.3 %.

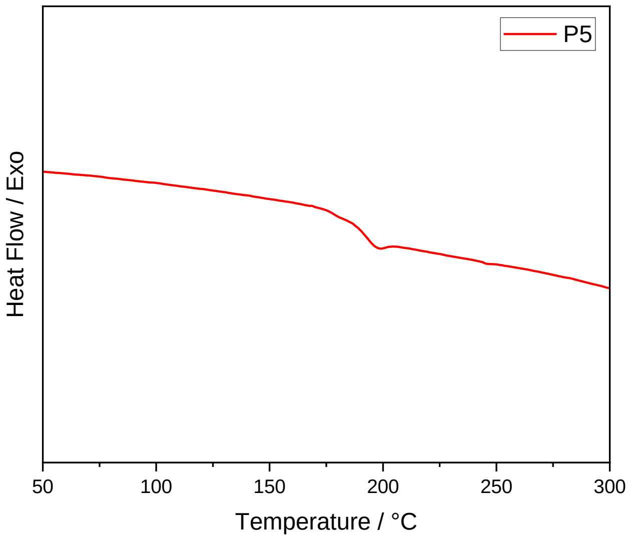

DSC: T_g = 194 °C.

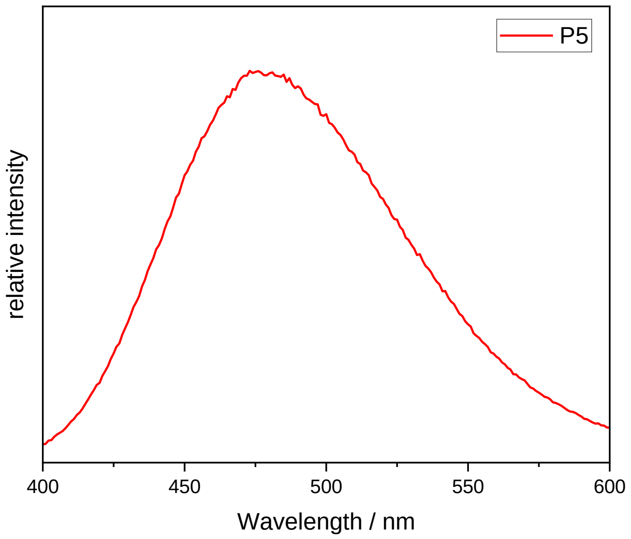

Fluorescence: $\lambda_{em,max}$ = 473 nm with λ_{ex} = 312 nm.

Synthesis of poly-(4,4'-biphenylene-1,2-diphenylvinylene) (**P6**)

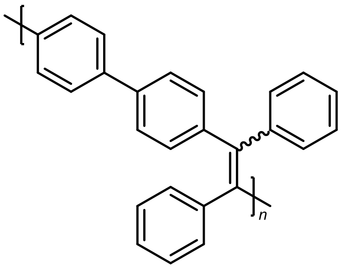

Synthesized from **M6** according to general procedure. Obtained as a yellow solid in a yield of 74% (122 mg, 0.368 mmol).

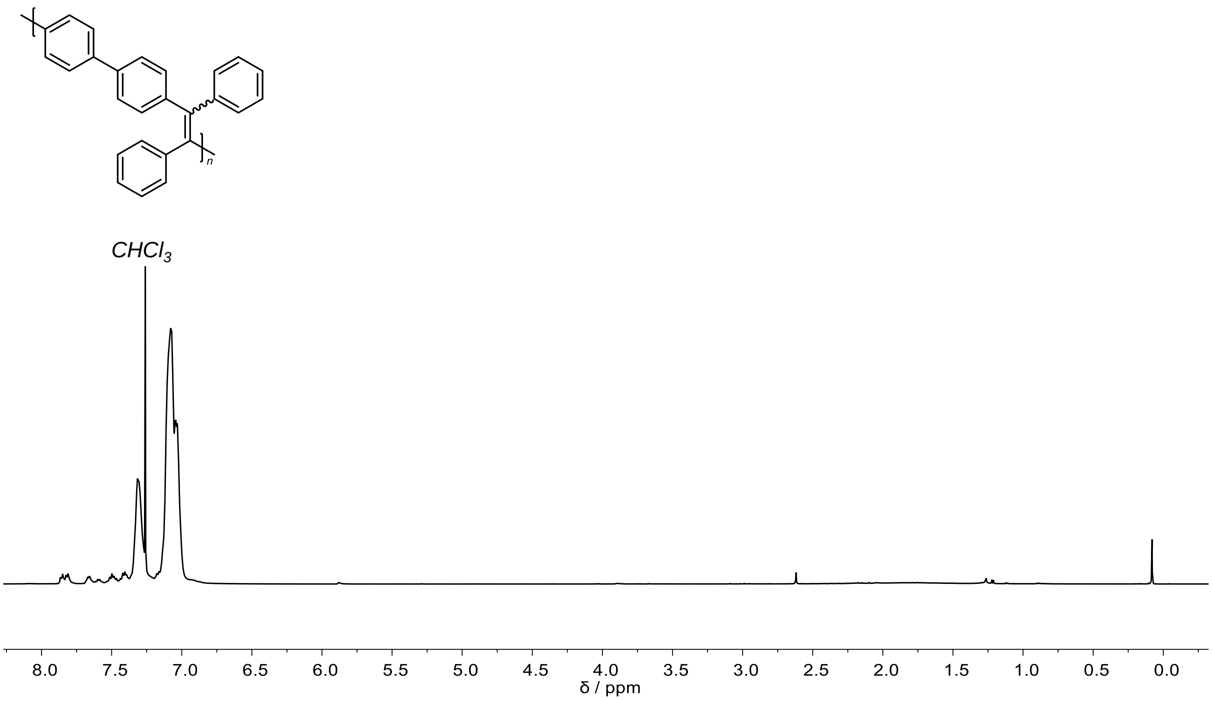

^{1}H NMR (500 MHz, Chloroform-*d*): δ/ppm 7.35 – 7.23 (m, 4H), 7.18 – 6.95 (m, 14H).

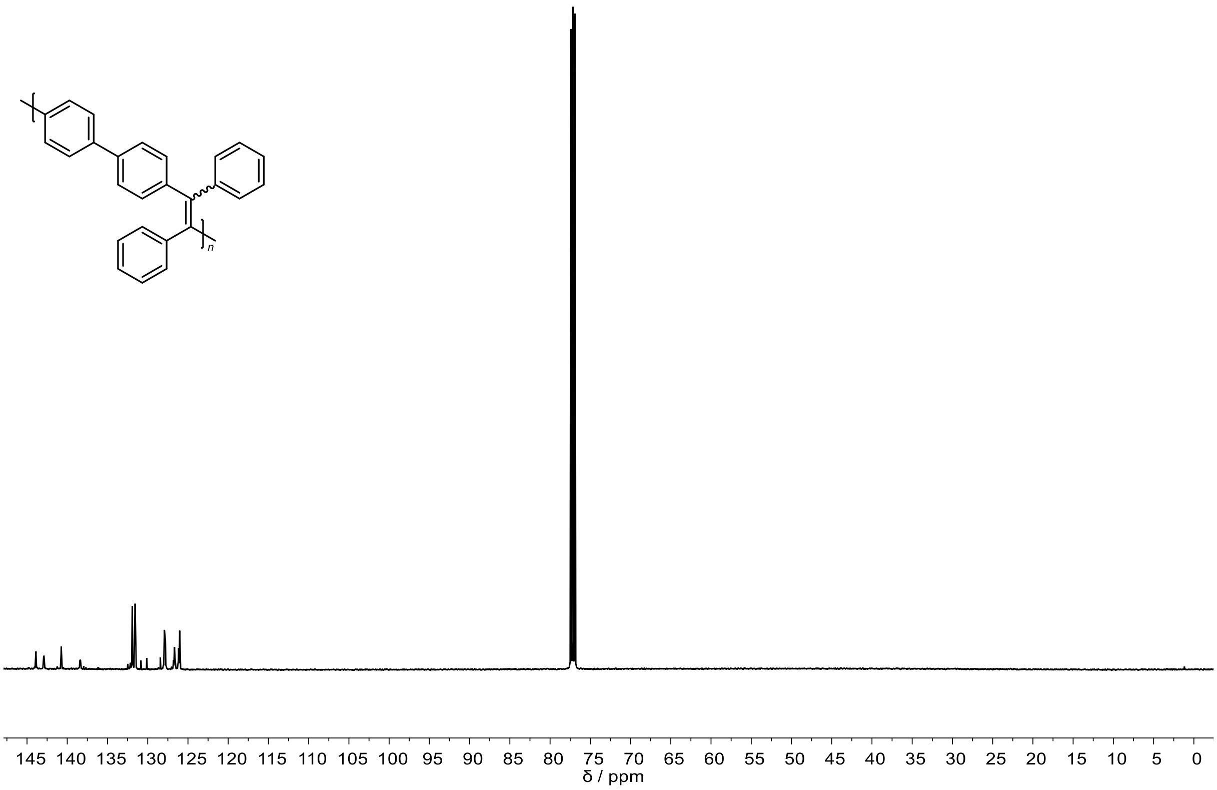

^{13}C NMR (126 MHz, Chloroform-*d*): δ/ppm = 144.12 – 143.59 (m), 143.10 – 142.60 (m), 140.88 – 140.51 (m), 138.62 – 138.05 (m), 132.05 – 131.84 (m), 128.12 – 127.65 (m), 126.98 – 126.49 (m), 126.33 – 125.72 (m).

IR (ATR platinum diamond): ν/cm^{-1} = 3075 (vw), 3048 (vw), 3024 (w), 1660 (vw), 1574 (vw), 1491 (m), 1442 (w), 1413 (vw), 1393 (w), 1312 (vw), 1275 (w), 1177 (vw), 1119 (w), 1074 (w), 1018 (w), 1002 (m), 975 (w), 938 (vw), 915 (w), 839 (w), 806 (m), 763 (m), 747 (m), 695 (vs), 664 (m), 625 (w), 619 (w), 603 (w), 574 (m), 547 (w), 539 (w), 490 (w), 481 (w), 473 (w), 463 (w).

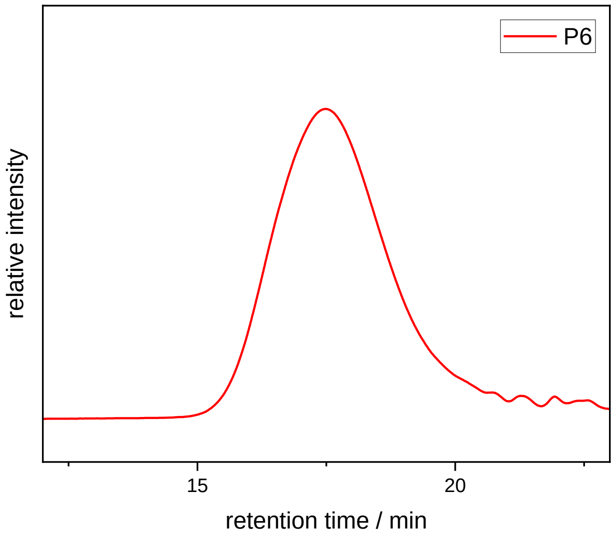

SEC (THF): M_n = 9.30 kDa, M_w = 15.0 kDa, $Đ$ = 1.61.

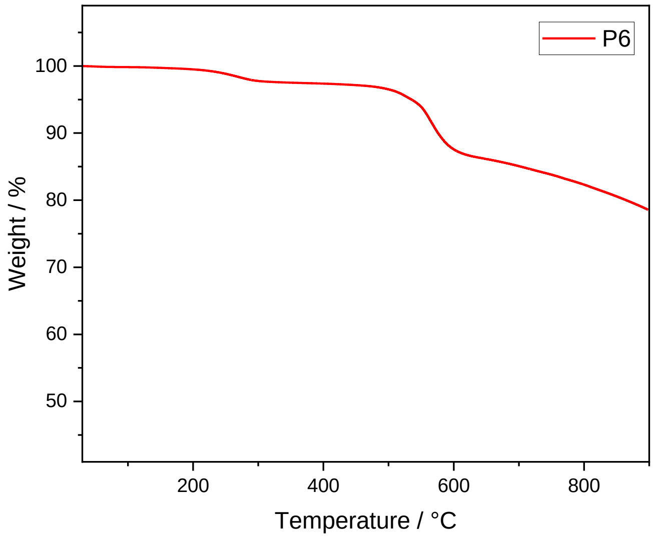

TGA: $T_{d,5\%}$ = 535 °C , Residue = 76.6 %.

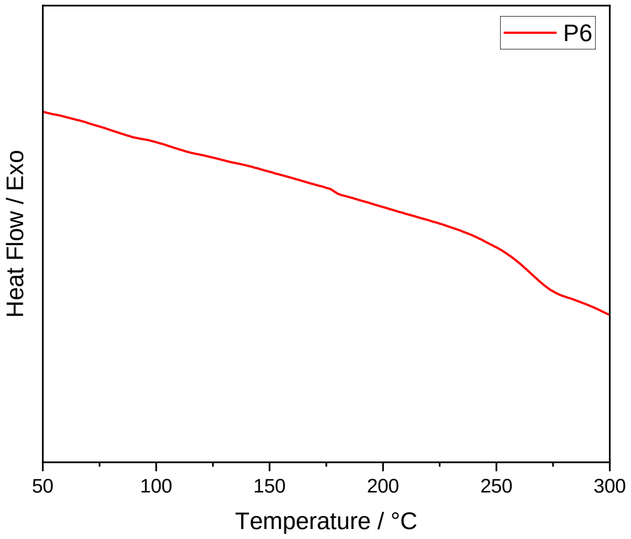

DSC: T_g = 263 °C.

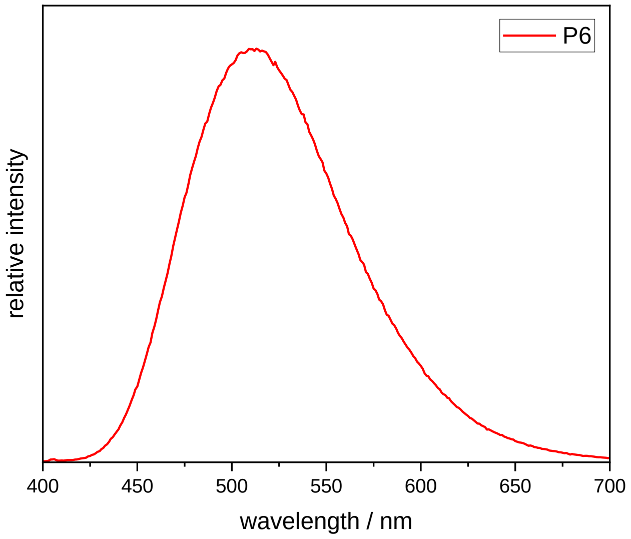

Fluorescence: $\lambda_{em,max}$ = 513 nm with λ_{ex} = 362 nm.

Synthesis of poly-(2,5-thienylene-1,2-diphenylvinylene) (**P7**)

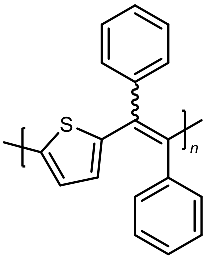

Synthesized from **M7** according to general procedure. Obtained as a dark red solid in a yield of 24% (30.6 mg, 0.118 mmol).

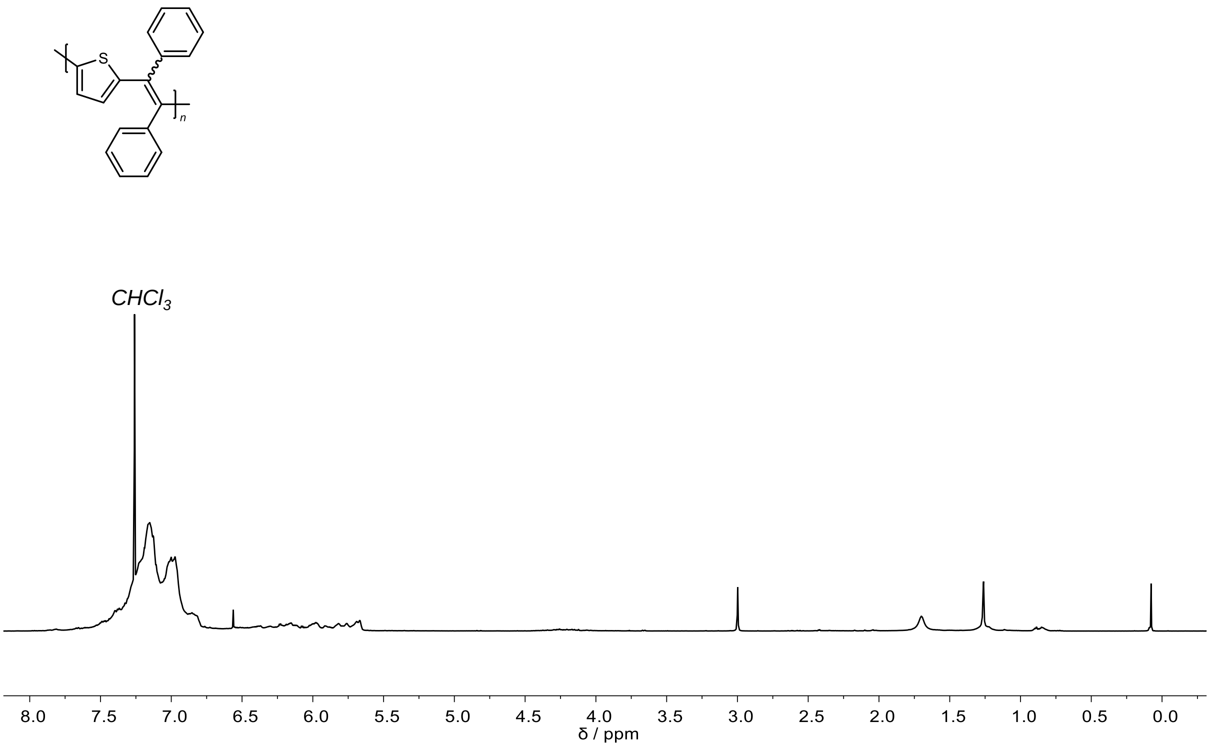

^{1}H NMR (500 MHz, Chloroform-*d*): δ/ppm = 7.54 – 6.68 (m, 12H).

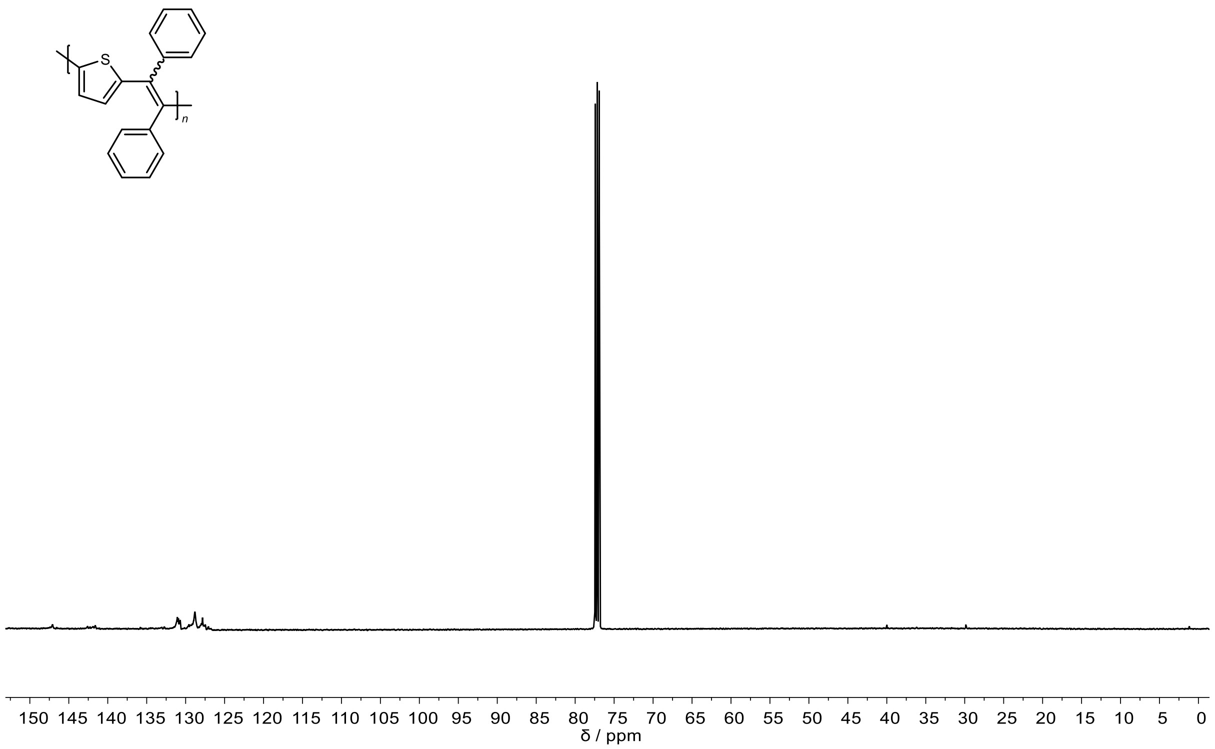

^{13}C NMR (126 MHz, Chloroform-*d*): δ 147.20 – 146.80 (m), 142.66 – 141.38 (m), 131.50 – 130.53 (m), 129.85 – 126.53 (m).

IR (ATR platinum diamond): *v*/cm^{-1} = 3055 (vw), 1598 (vw), 1489 (w), 1442 (w), 1275 (vw), 1261 (vw), 1220 (vw), 1177 (vw), 1156 (vw), 1107 (vw), 1072 (w), 1028 (w), 915 (vw), 841 (vw), 804 (w), 759 (w), 736 (w), 726 (m), 693 (vs), 640 (w), 617 (vw), 584 (vw), 496 (vw), 453 (vw), 444 (vw), 438 (vw).

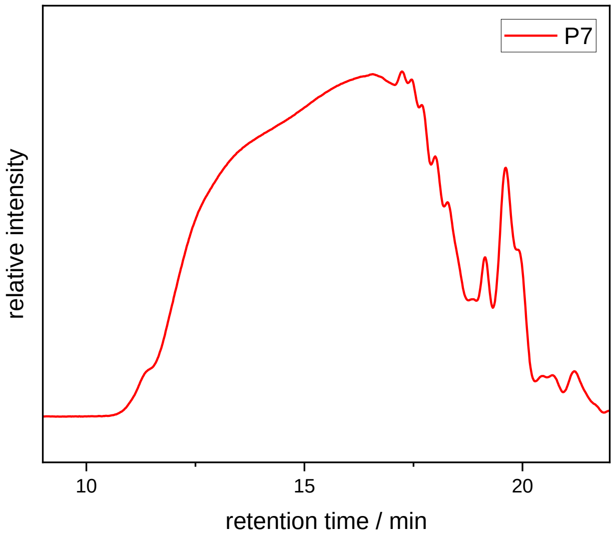

SEC (THF): M_n = 1.67 kDa, M_w = 8.28 kDa, *Đ* = 4.97. Note that a different set of columns [two PSS SDV analytical columns (3 µm, 300 × 8.0 mm, 1000 Å) with one PSS SDV analytical precolumn (3 µm, 50 × 8.0 mm)] was used for analysis due to the low molecular weight of **P7**.

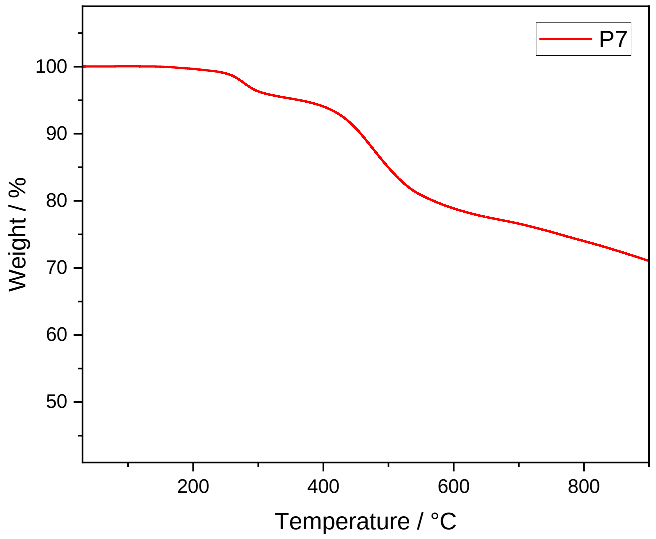

TGA: $T_{d,5\%}$ = 430 °C , Residue = 71.1 °C.

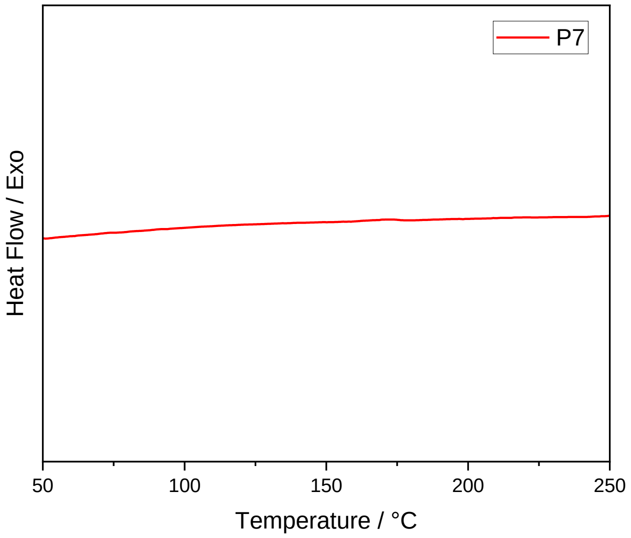

DSC: No glass transition could be detected.

Fluorescence: Since the polymer was not fluorescent, no fluorescence spectra were recorded.

*Synthesis of poly-(1,4-phenylene-1,2-bis(4-*tert*-butylphenyl)vinylene)* (**P8**)

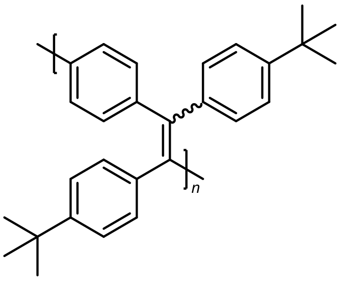

Synthesized from **M8** according to general procedure. Obtained as a yellow solid in a yield of 73% (134 mg, 0.366 mmol).

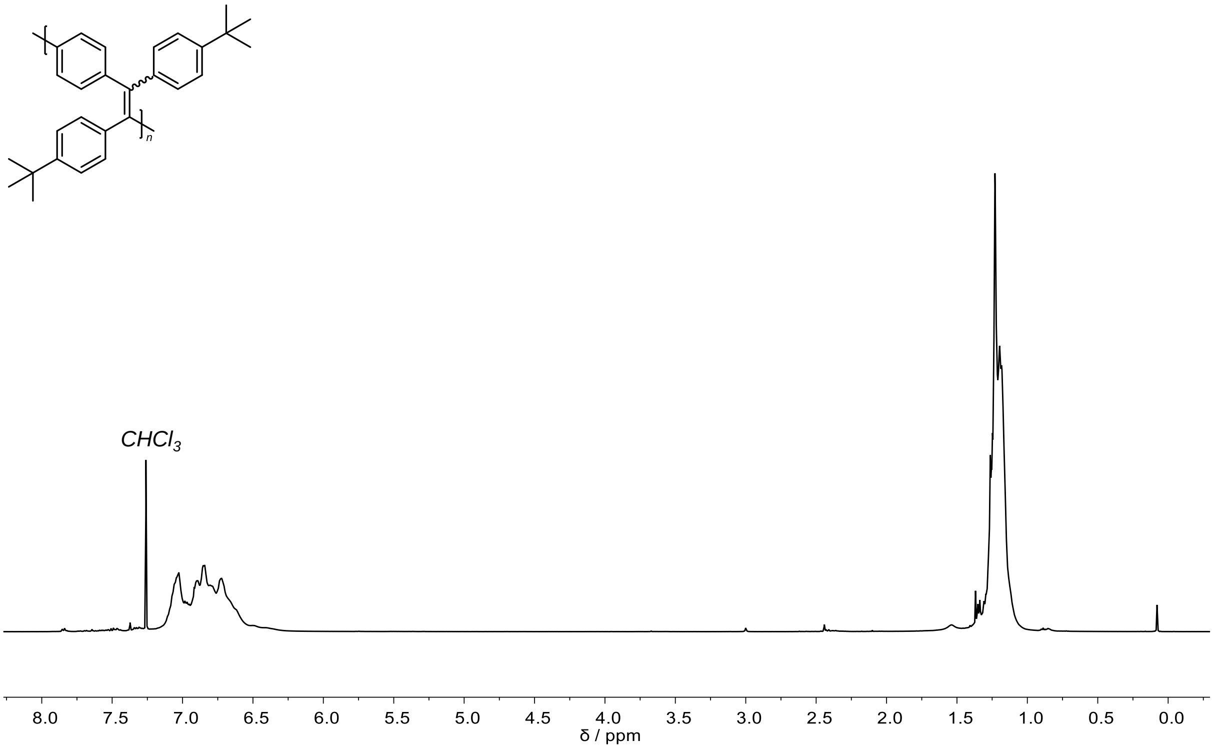

^{1}H NMR (500 MHz, Chloroform-*d*): δ/ppm = 7.18 – 6.54 (m, 12H), 1.40 – 1.01 (m, 18H).

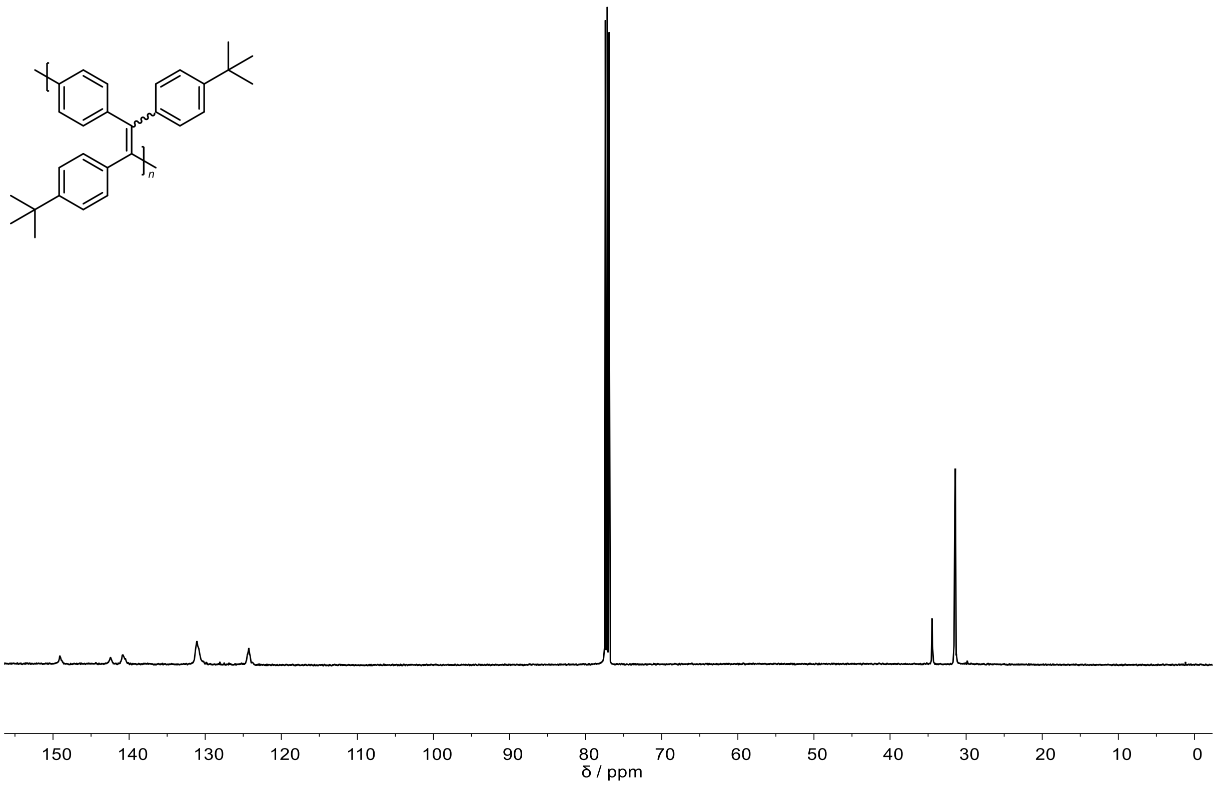

^{13}**C NMR** (126 MHz, Chloroform-*d*): δ/ppm = 149.48 – 148.40 (m), 142.81 – 141.95 (m), 141.50 – 140.15 (m), 132.05 – 129.72 (m), 125.03 – 123.61 (m), 35.47 – 33.79 (m), 33.04 – 30.79 (m).

IR (ATR platinum diamond): *v*/cm^{-1} = 3028 (w), 2958 (s), 2902 (m), 2867 (m), 1506 (m), 1475 (w), 1460 (m), 1395 (m), 1362 (m), 1269 (m), 1201 (w), 1164 (w), 1107 (m), 1018 (m), 977 (w), 860 (w), 829 (vs), 792 (s), 755 (w), 685 (m), 666 (m), 627 (w), 574 (s), 543 (m), 504 (w), 494 (w).

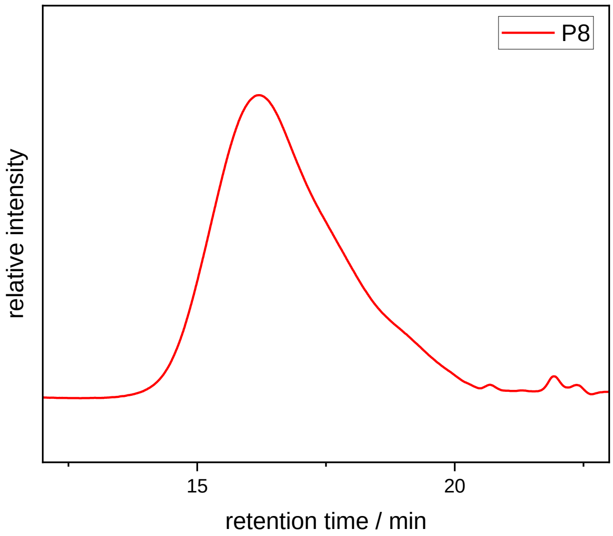

SEC (THF): M_n = 14.9 kDa, M_w = 33.5 kDa, $Đ$ = 2.19.

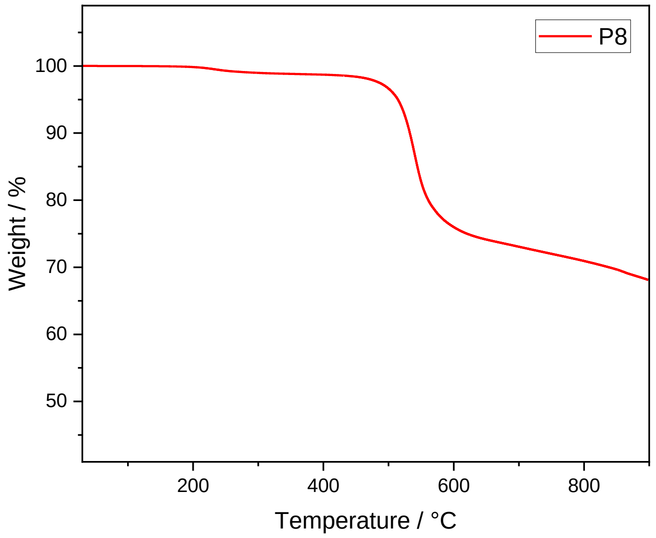

TGA: $T_{d,5\%}$ = 514 °C , Residue = 68.1 %.

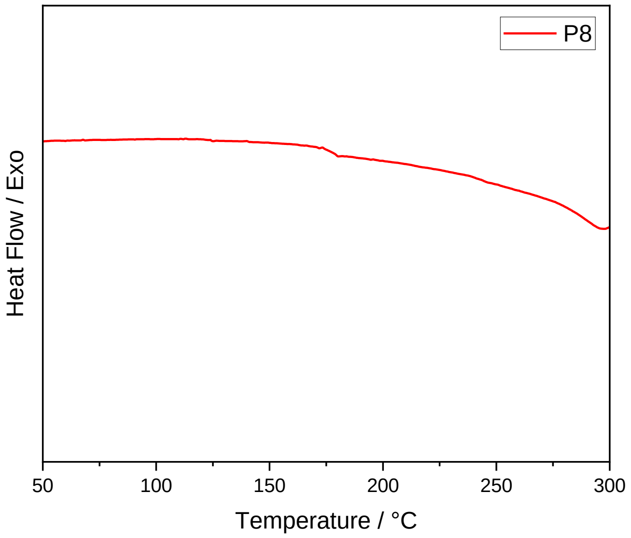

DSC: T_g = 175 °C.

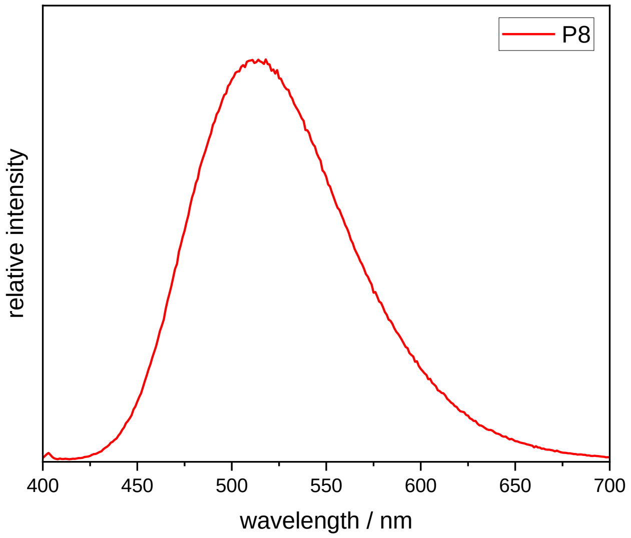

Fluorescence: $\lambda_{em,max}$ = 514 nm with λ_{ex} = 360 nm.

*Synthesis of a copolymer from **M1** and **M2*** (**CP1**)

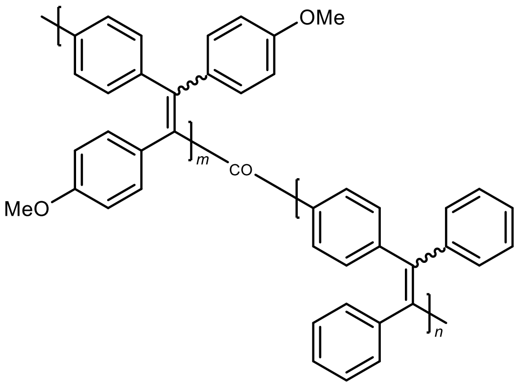

Synthesized from **M1** and **M2** in a 1:1 ratio according to general procedure. Obtained as a yellow solid in a yield of 64% (90.7 mg, 0.159 mmol).

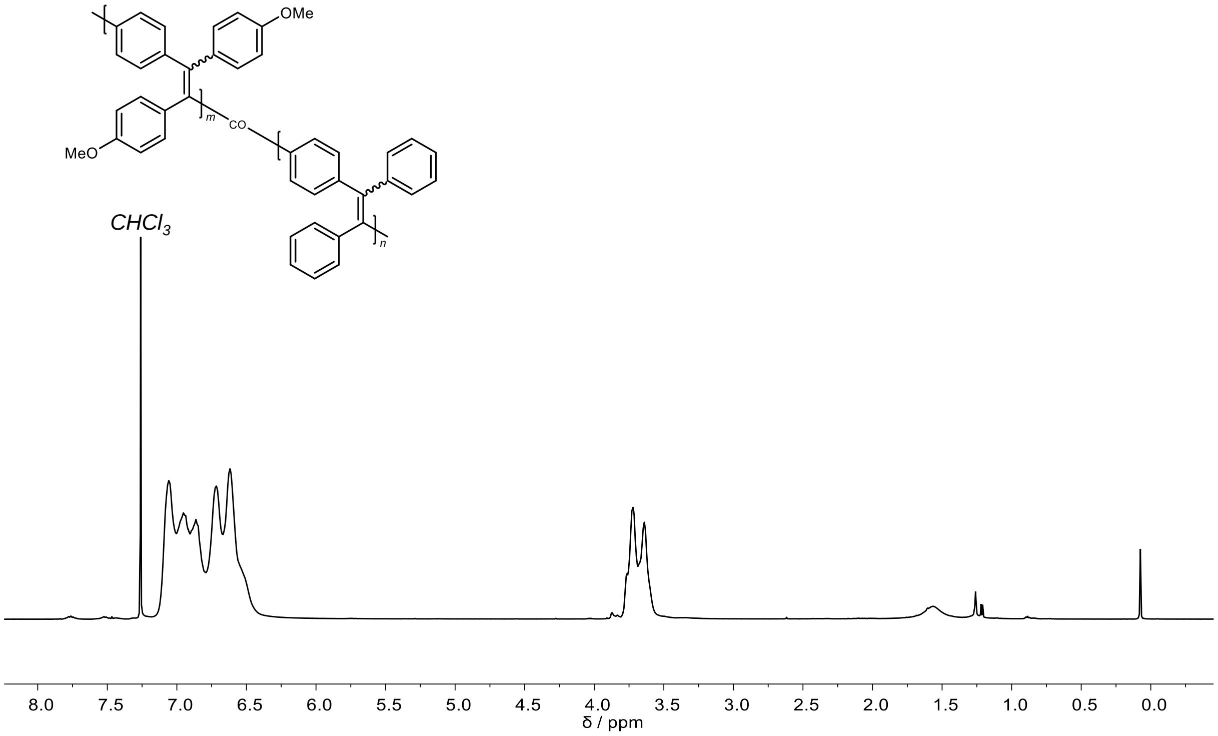

^{1}H NMR (500 MHz, Chloroform-*d*): δ/ppm = 7.16 – 6.41 (m, 26H), 3.82 – 3.50 (m, 6H).

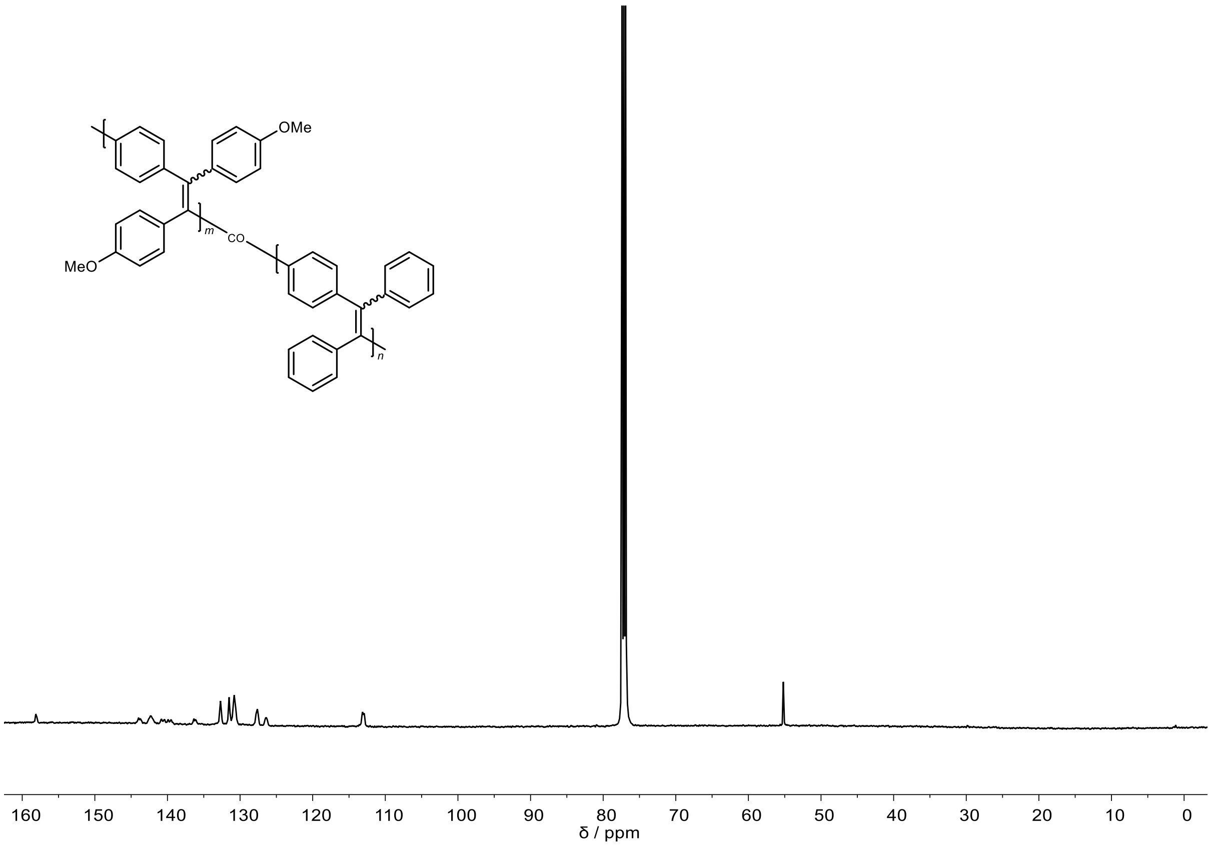

^{13}C NMR (126 MHz, Chloroform-*d*): δ/ppm = 158.59 – 157.46 (m), 144.53 – 143.28 (m), 142.74 – 141.49 (m), 136.73 – 135.82 (m), 133.63 – 132.03 (m), 132.01 – 131.28 (m), 131.14 – 130.31 (m), 128.12 – 127.19 (m), 126.73 – 126.11 (m), 113.46 – 112.49 (m), 55.31 – 55.02 (m).

IR (ATR platinum diamond): ν/cm^{-1} = 3052 (vw), 3024 (vw), 2995 (vw), 2933 (vw), 2902 (vw), 2832 (vw), 1604 (m), 1574 (w), 1506 (s), 1462 (w), 1442 (m), 1401 (w), 1288 (m), 1242 (vs), 1172 (s), 1137 (w), 1109 (m), 1074 (w), 1033 (m), 975 (w), 930 (vw), 913 (vw), 858 (w), 829 (m), 802 (m), 784 (w), 761 (m), 747 (m), 697 (s), 671 (w), 629 (w), 617 (w), 572 (w), 551 (w), 543 (w), 525 (w), 516 (w), 479 (w), 469 (w), 455 (vw), 440 (vw).

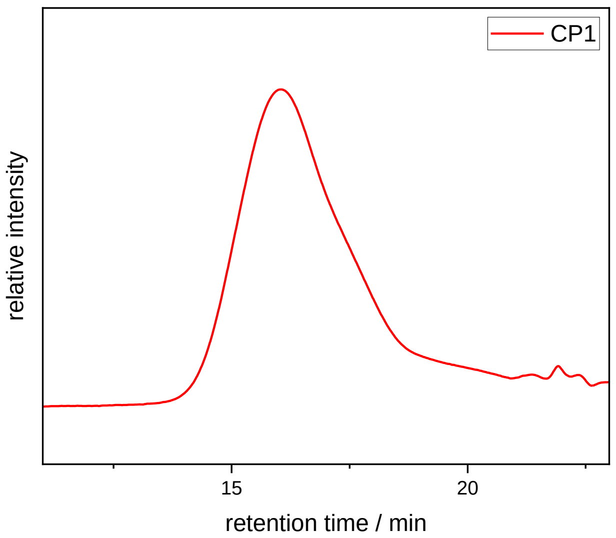

SEC (THF): M_n = 19.2 kDa, M_w = 39.0 kDa, $Đ$ = 2.03.

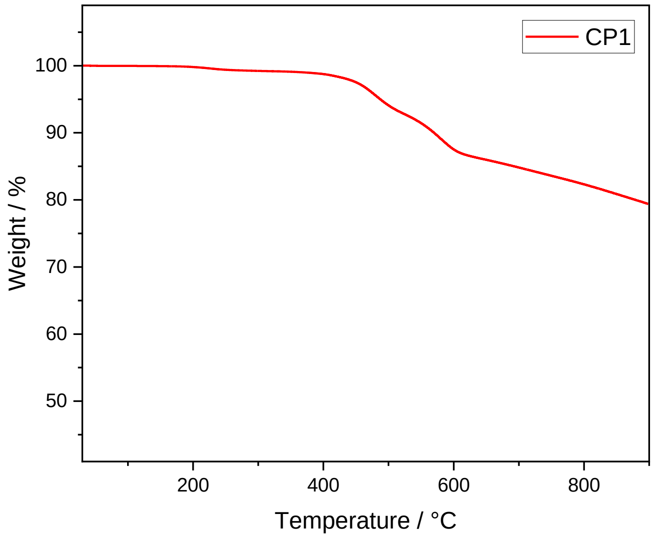

TGA: $T_{d,5\%}$ = 487 °C , Residue = 79.4 %.

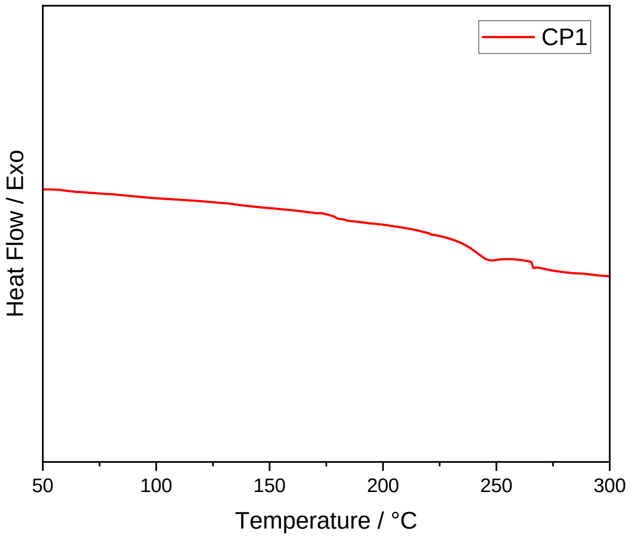

DSC: T_g = 241 °C.

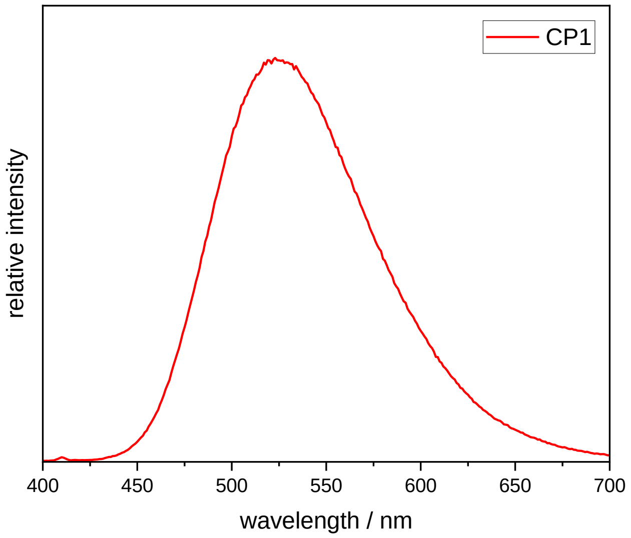

Fluorescence: $\lambda_{em,max}$ = 523 nm with λ_{ex} = 366 nm.

*Synthesis of a copolymer from **M2** and **M5*** (**CP2**)

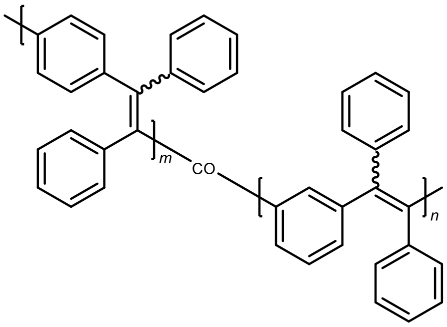

Synthesized from **M2** and **M5** in a 1:1 according to general procedure. Obtained as a light yellow solid in a yield of 63% (80.0 mg, 0.157 mmol) .

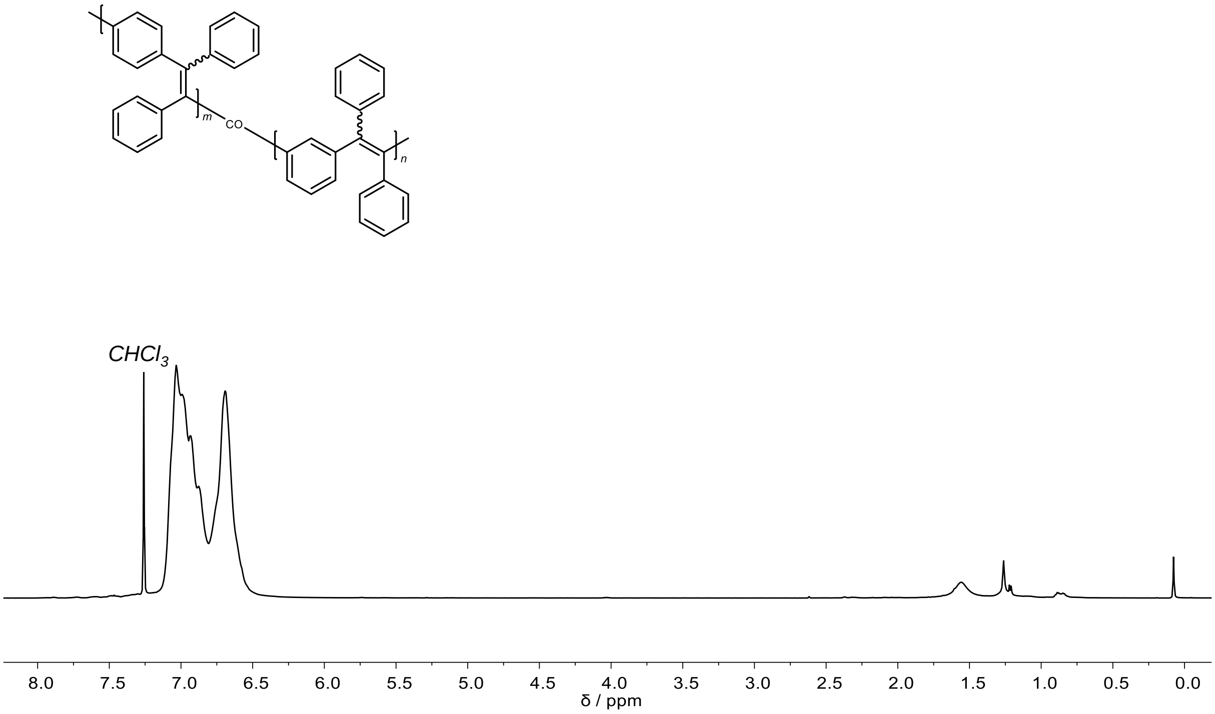

^{1}H NMR (500 MHz, Chloroform-*d*): δ/ppm = 7.17 – 6.49 (m, 28H).

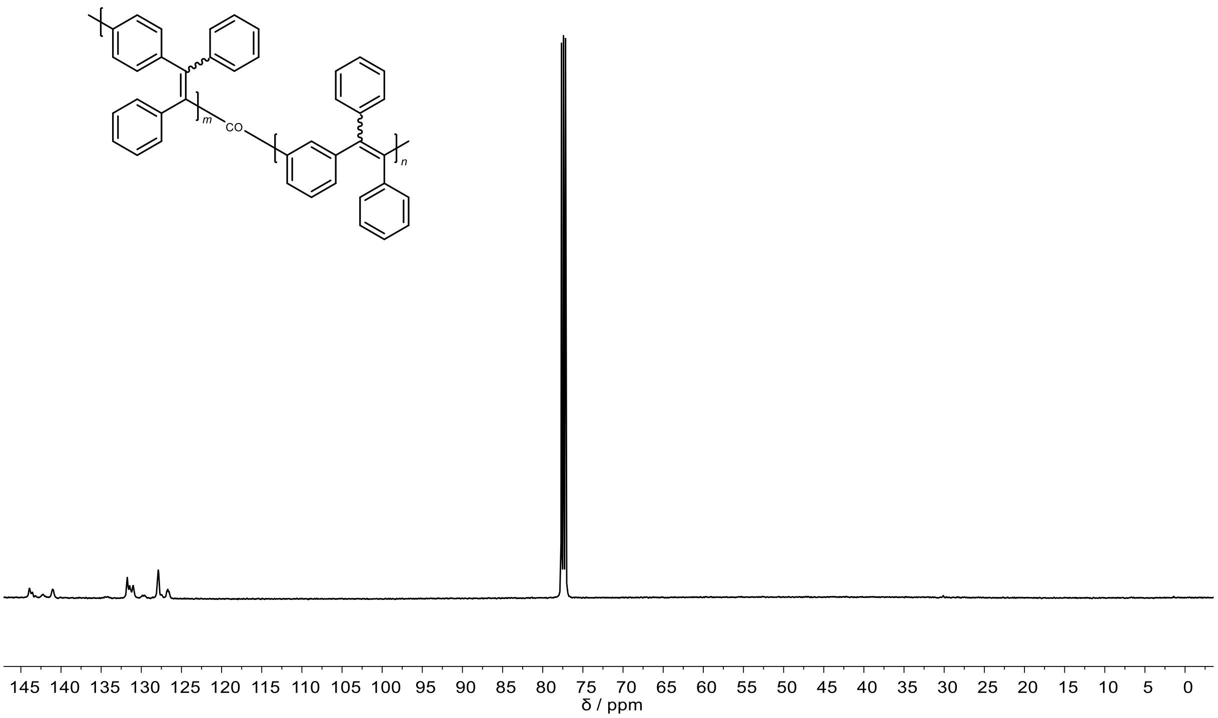

^{13}C NMR (126 MHz, Chloroform-*d*): δ/ppm = 144.42 – 143.25 (m), 142.54 – 141.82 (m), 141.48 – 140.55 (m), 132.33 – 130.35 (m), 130.16 – 129.29 (m), 128.55 – 127.26 (m), 127.13 – 126.05 (m).

IR (ATR platinum diamond): *ν*/cm^{-1} = 3052 (vw), 3020 (vw), 1596 (vw), 1504 (vw), 1491 (w), 1442 (w), 1154 (vw), 1137 (w), 1111 (vw), 1074 (w), 1031 (vw), 1020 (vw), 1008 (w), 998 (w), 913 (vw), 854 (vw), 843 (vw), 790 (vw), 759 (w), 695 (vs), 673 (w), 629 (w), 619 (w), 603 (vw), 566 (w), 539 (vw), 485 (vw), 473 (vw).

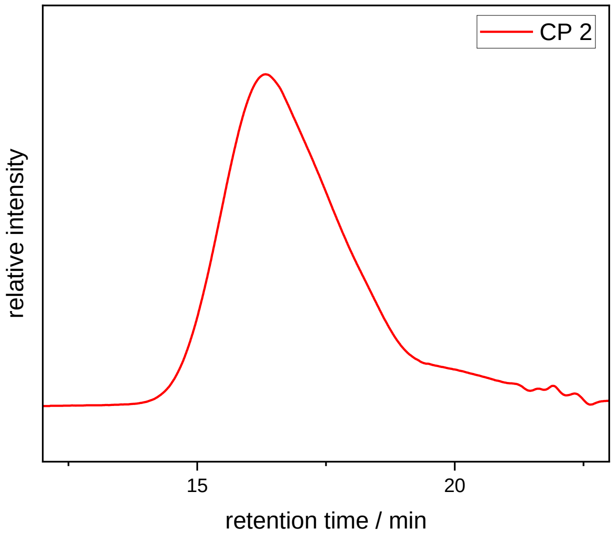

SEC (THF): M_n = 15.1 kDa, M_w = 30.1 kDa, $Đ$ = 1.99.

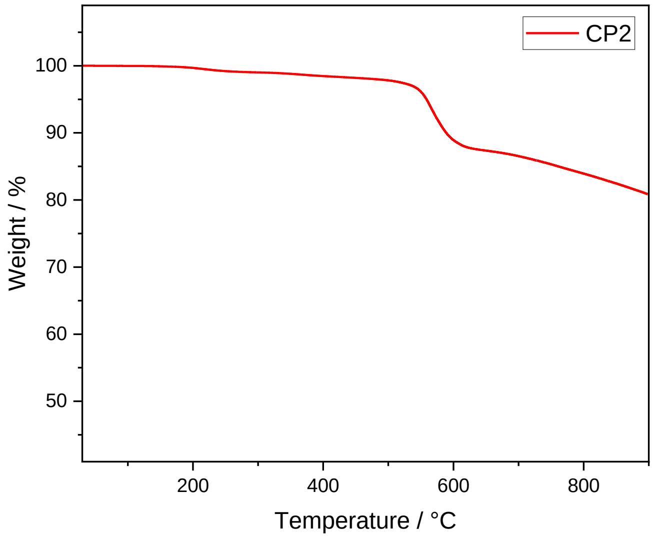

TGA: $T_{d,5\%}$ = 558 °C , Residue = 80.9 %.

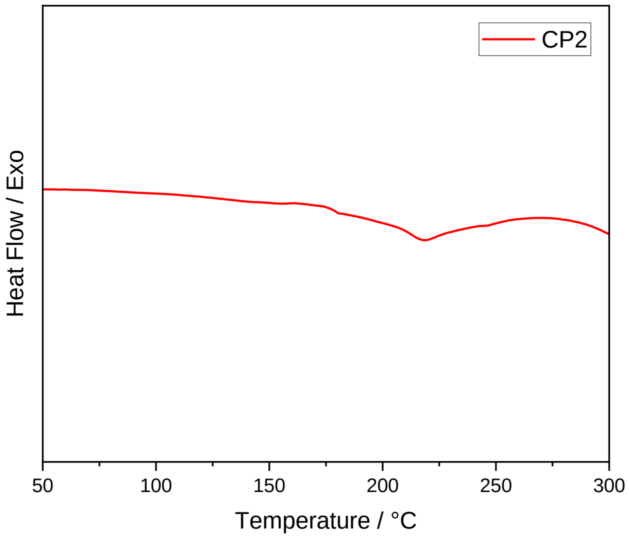

DSC: T_g = 214 °C.

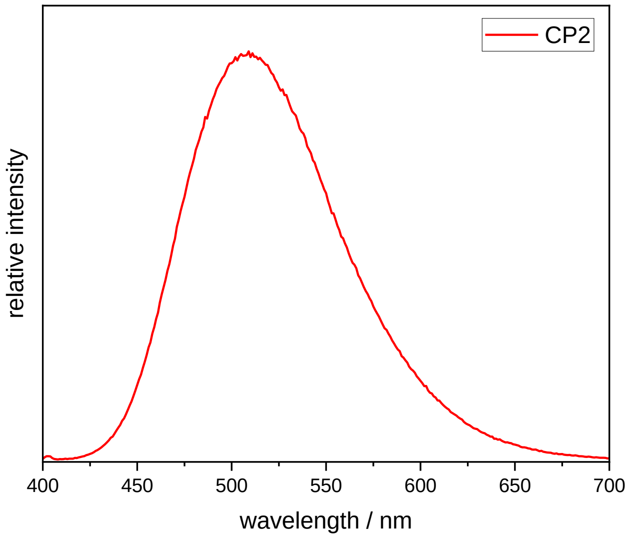

Fluorescence: $\lambda_{em,max}$ = 505 nm with λ_{ex} = 360 nm.

3.3 Other syntheses

Synthesis of 4,4'-dimethoxybenzophenone NTH (**2**)

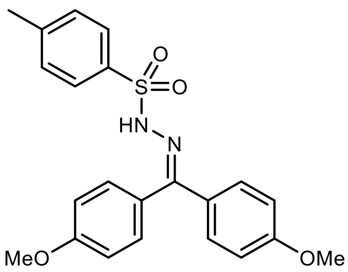

The procedure was adapted from Meier *et al.*[2]
4,4'-Dimethoxybenzophenone (**1**) (1.21 g, 5.00 mmol, 1.00 equiv.) and tosyl hydrazide (1.12 g, 6.00 mmol, 1.20 equiv.) were dissolved in 5 mL EtOH and *p*-toluenesulfonic acid monohydrate (47.6 mg, 0.25 mmol, 0.10 equiv.) was added. The mixture was refluxed overnight. The precipitate was filtered and washed with cold EtOH. The product was obtained as an off-white powder in a yield of 93% (1.92 g, 4.67 mmol).

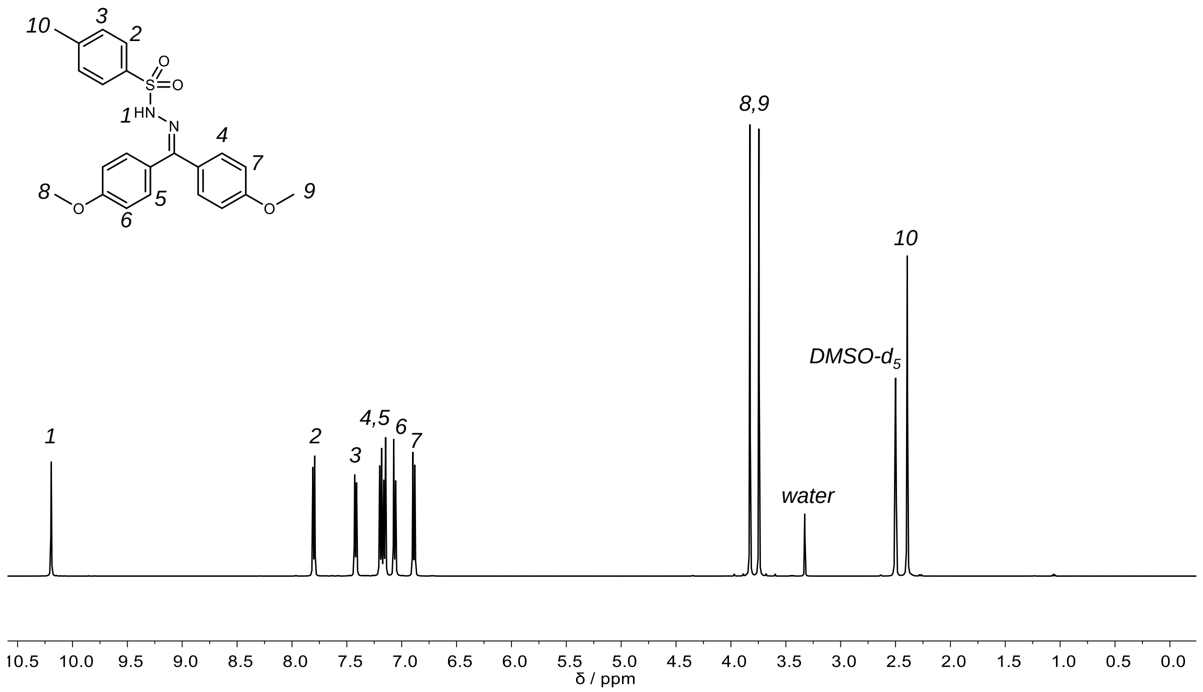

^{1}H NMR (500 MHz, DMSO-d_6): δ/ppm = 10.19 (s, 1H), 7.83 – 7.78 (m, 2H), 7.45 – 7.39 (m, 2H), 7.22 – 7.16 (m, 2H), 7.19 – 7.12 (m, 2H), 7.10 – 7.03 (m, 2H), 6.92 – 6.86 (m, 2H), 3.83 (s, 3H), 3.74 (s, 3H), 2.39 (s, 3H).

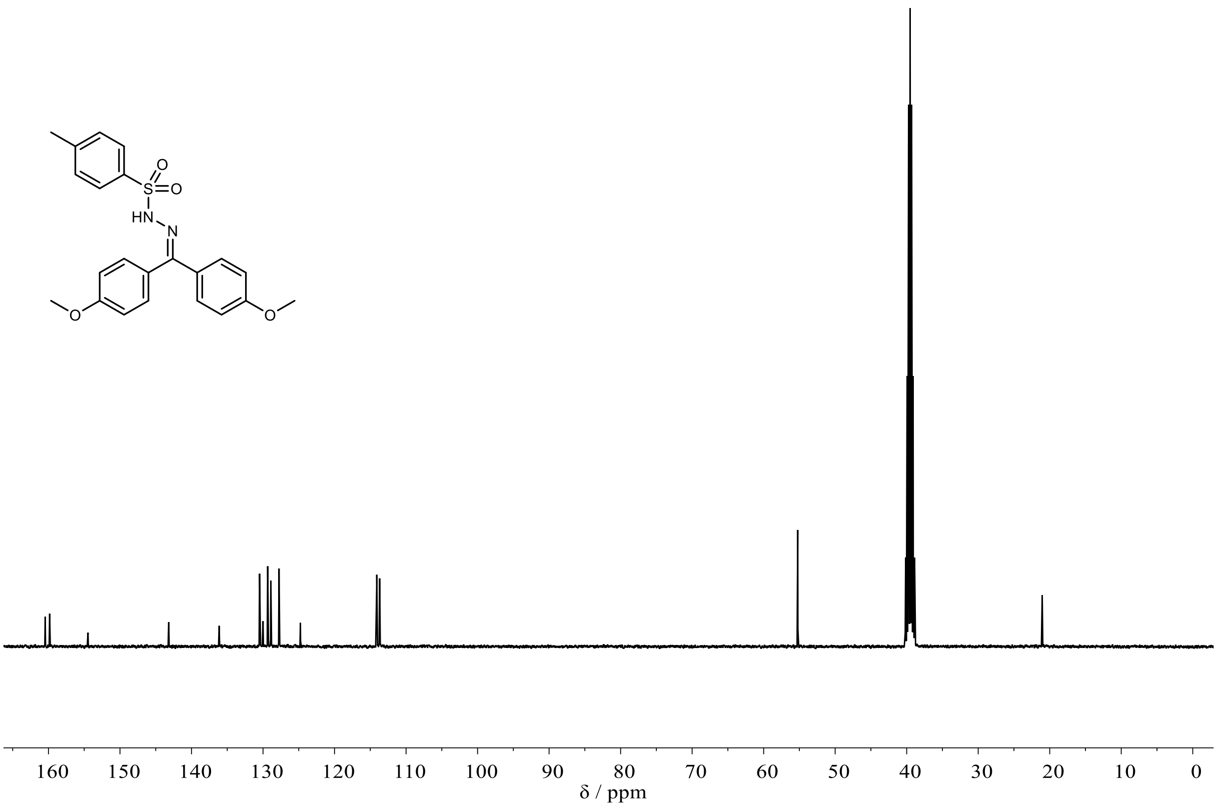

^{13}C NMR (126 MHz, DMSO-d_6): δ/ppm = 160.46, 159.84, 154.47, 143.18, 136.13, 130.48, 130.00, 129.35, 128.91, 127.77, 124.77, 114.10, 113.69, 55.23, 21.05.

IR (ATR platinum diamond): *v*/cm^{-1} = 3201 (w), 3065 (vw), 3028 (vw), 2956 (vw), 2925 (vw), 2832 (vw), 1606 (s), 1578 (w), 1510 (s), 1460 (w), 1440 (w), 1419 (w), 1386 (m), 1341 (m), 1310 (s), 1300 (m), 1249 (vs), 1187 (w), 1174 (s), 1158 (vs), 1113 (w), 1092 (w), 1053 (m), 1031 (vs), 1012 (m), 983 (s), 954 (w), 887 (m), 831 (vs), 810 (s), 782 (w), 734 (w), 706 (m), 689 (m), 664 (vs), 597 (w), 578 (w), 545 (vs), 518 (m), 498 (m), 481 (w), 444 (w).

Synthesis of p-*nosyl hydrazide*

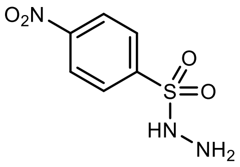

The procedure was adapted from Serrano *et al.*[3]
Hydrazine hydrate (2.50 mL, 4.43 g, 125 mmol, 2.50 equiv.) was dissolved in 8 mL of tetrahydrofuran (THF) and cooled to 0 °C. *p*-Nosyl chloride (2.50 g, 50 mmol, 1.00 equiv.) was added in small portions. After complete addition, the mixture was stirred for 2 hours at 0 °C. Afterwards, 40 mL of water were added, the precipitate was filtered, washed with water and dried *in vacuo*. The product was obtained as a white solid in a yield of 86 % (3.74 g, 17.2 mmol).

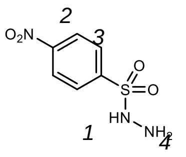

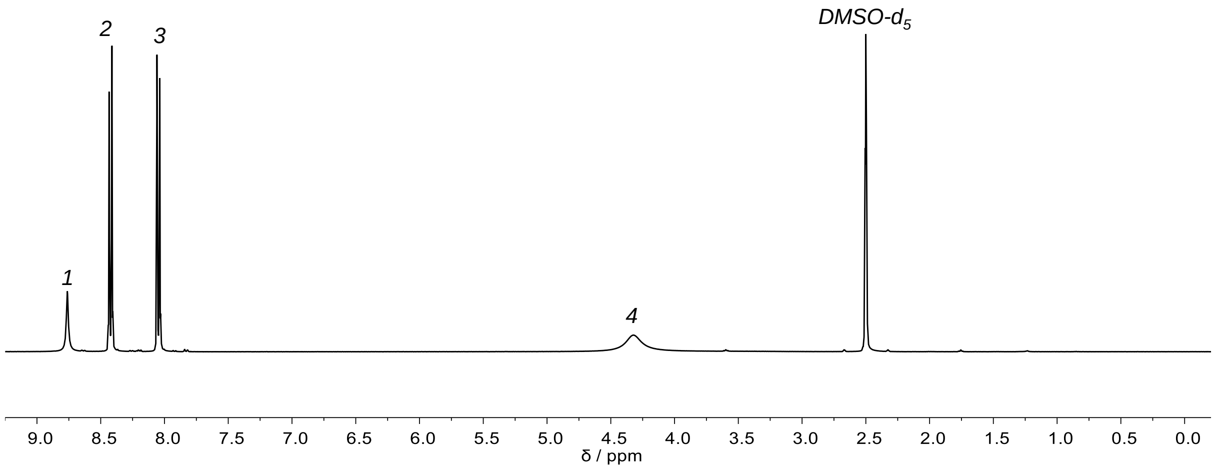

^{1}H NMR (400 MHz, DMSO-*d*6): δ/ppm = 8.76 (s, 1H), 8.46 – 8.38 (m, 1H), 8.09 – 8.01 (m, 1H), 4.32 (s, 1H).

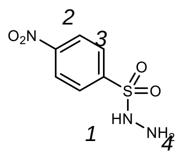

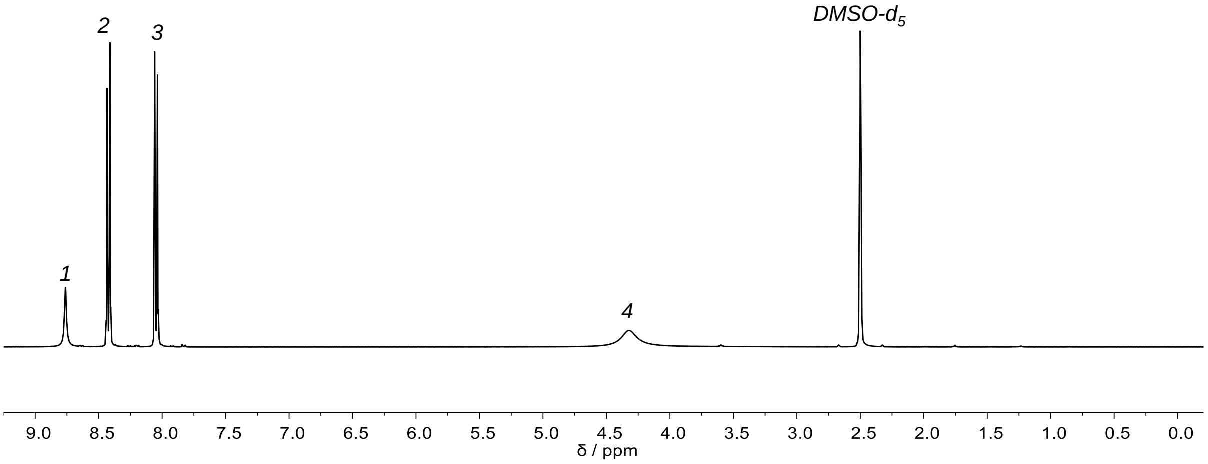

^{13}C NMR (101 MHz, DMSO-d_6): δ/ppm = 149.71, 144.20, 129.26, 124.24.

IR (ATR platinum diamond): *v*/cm-1 = 3258 (m), 3102 (w), 3013 (w), 2972 (w), 2960 (w), 2943 (w), 2913 (w), 2900 (w), 2837 (w), 1604 (m), 1565 (w), 1526 (s), 1504 (s), 1467 (w), 1442 (w), 1419 (w), 1401 (w), 1384 (s), 1343 (s), 1308 (s), 1288 (m), 1244 (vs), 1177 (vs), 1160 (s), 1115 (m), 1107 (m), 1090 (m), 1047 (m), 1028 (s), 1010 (m), 977 (s), 959 (m), 938 (w), 880 (m), 854 (s), 831 (s), 815 (s), 784 (m), 734 (s), 683 (s), 613 (s), 594 (s), 576 (m), 551 (s), 527 (s), 494 (m), 459 (s), 438 (m).

Synthesis of 4,4'-dimethoxybenzophenone p-*nosylhydrazone* (**3**)

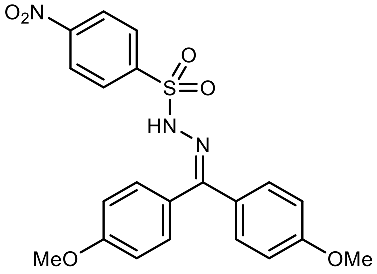

4,4'-Dimethoxybenzophenone (**1**) (1.21 g, 5.00 mmol, 1.00 equiv.) and *p*-nosyl hydrazide (1.30 g, 6.00 mmol, 1.20 equiv.) were dissolved in 5 mL EtOH and *p*-toluenesulfonic acid monohydrate (47.6 mg, 0.25 mmol, 0.10 equiv.) were added. The mixture was refluxed overnight. The precipitate was filtered and washed with cold EtOH. The product was obtained as a yellow in a yield of 92% (2.02, 4.61 mmol).

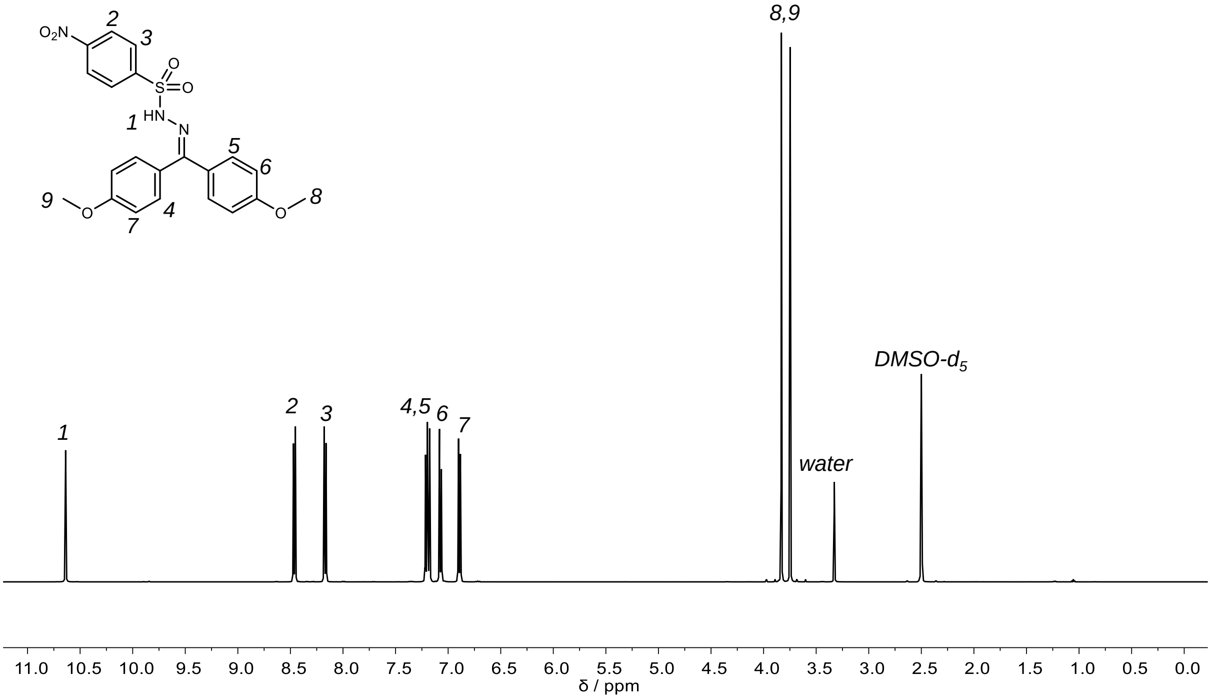

^{1}H NMR (500 MHz, DMSO-*d*6): δ/ppm = 10.64 (s, 1H), 8.49 – 8.43 (m, 2H), 8.20 – 8.14 (m, 2H), 7.24 – 7.15 (m, 4H), 7.11 – 7.04 (m, 2H), 6.93 – 6.86 (m, 2H), 3.83 (s, 3H), 3.75 (s, 3H).

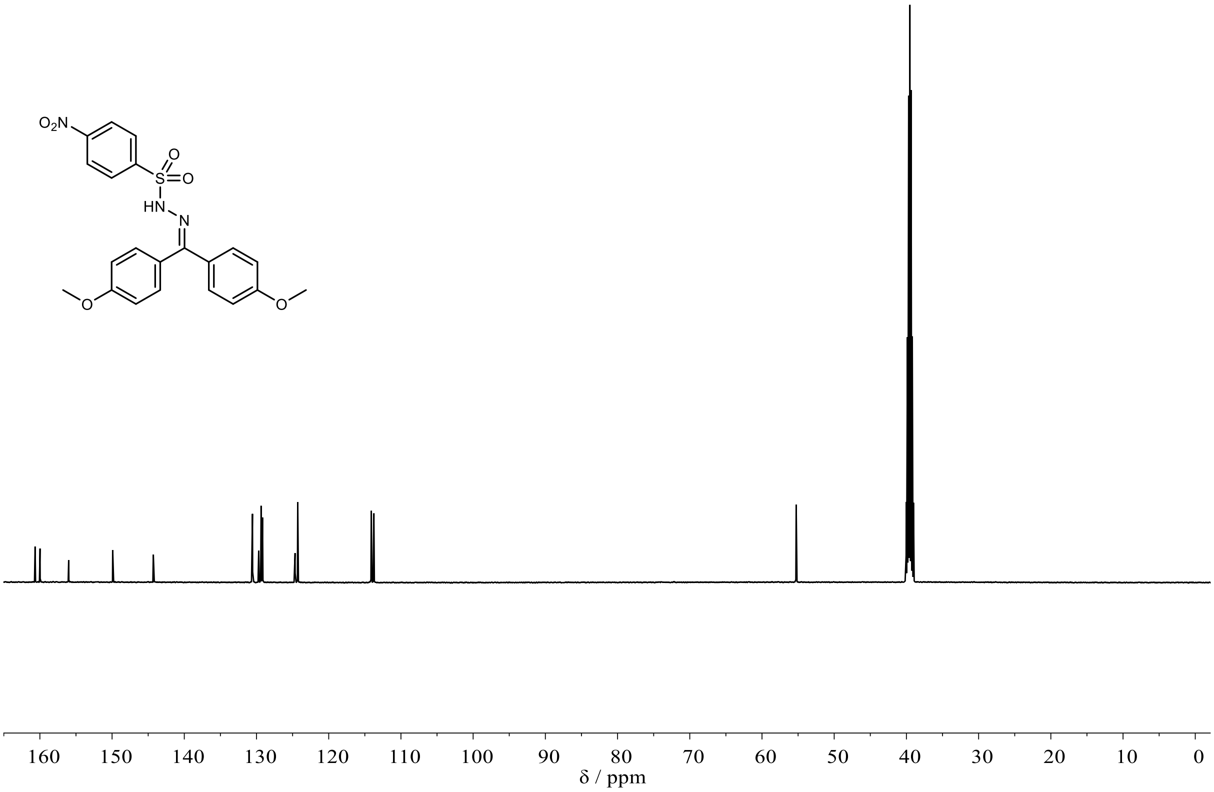

^{13}C NMR (126 MHz, DMSO-*d*$_6$): δ/ppm = 160.66, 159.98, 156.01, 149.92, 144.29, 130.56, 129.69, 129.36, 129.17, 124.66, 124.29, 114.11, 113.75, 55.27, 55.25.

IR (ATR platinum diamond): *v*/cm^{-1} = 3258 (m), 3102 (w), 3061 (vw), 3013 (w), 2972 (w), 2960 (w), 2943 (w), 2913 (w), 2900 (w), 2837 (w), 1604 (m), 1565 (w), 1526 (s), 1504 (s), 1467 (w), 1442 (w), 1419 (w), 1401 (w), 1384 (s), 1343 (s), 1308 (s), 1288 (m), 1244 (vs), 1177 (vs), 1160 (s), 1115 (m), 1107 (m), 1090 (m), 1047 (m), 1028 (s), 1010 (m), 977 (s), 959 (m), 938 (w), 880 (m), 854 (s), 831 (s), 815 (s), 784 (m), 734 (s), 683 (s), 613 (s), 594 (s), 576 (m), 551 (s), 527 (s), 494 (m), 459 (s), 438 (m).

Synthesis of o-*nosyl hydrazide*

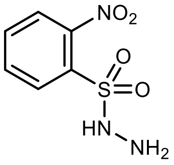

The procedure was adapter from Myers *et al*.[4]
In a Schlenk flask under argon atmosphere, *o*-nosyl chloride (2.22 g, 10.0 mmol, 1.00 equiv.) was dissolved in 20 mL of THF and the solution was cooled to -30 °C (bromobenzene / dry ice bath). Afterwards, hydrazine hydrate (1.23 mL, 1.25 g, 25.0 mmol, 2.50 equiv.) was added dropwise over a period of 10 minutes. The mixture was stirred at -30 °C for 1 hour. Afterwards, 20 mL of ethyl acetate were added and the organic phase was extracted 5 times with 15 mL of 10% NaCl solution. The organic phase was dried over sodium sulfate and precipitated in 120 mL of *n*-hexane. The precipitate was filtered, washed with n-hexane and dried under vacuum. The product was obtained as an off-white solid in a yield of 58% (1.26 g, 5.80 mmol).

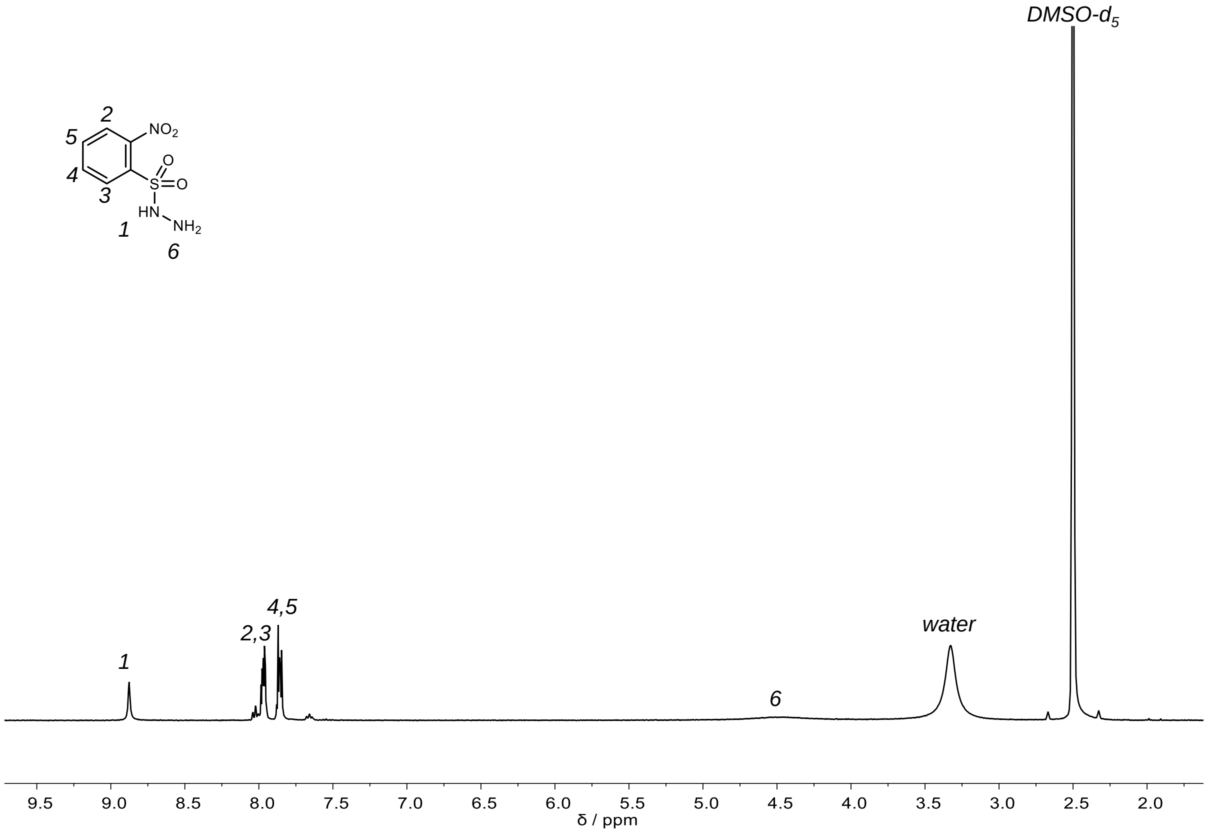

^{1}H NMR (400 MHz, DMSO-d_6): δ/ppm = 8.88 (s, 1H), 8.07 – 7.92 (m, 2H), 7.86 (dt, *J* = 5.4, 3.7 Hz, 2H), 4.47 (s, 2H).

Note: Due to the thermal instability of the compound in solution, the unassigned signals correspond to the decomposition products (presumably the corresponding sulfinic acid). No ^{13}C NMR spectra were recorded for this reason.

Synthesis of trisyl hydrazide

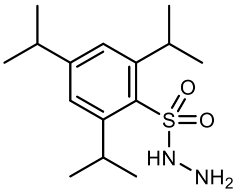

The procedure was adapter from Myers *et al.*[4]
In a Schlenk flask under argon atmosphere, trisyl chloride (3.03 g, 10.0 mmol, 1.00 equiv.) was dissolved in 20 mL of THF and the solution was cooled to -30 °C (bromobenzene / dry ice bath). Afterwards, hydrazine hydrate (1.23 mL, 1.25 g, 25.0 mmol, 2.50 equiv.) was added dropwise over a period of 10 minutes. The mixture was stirred at -30 °C for 1 hour. Afterwards, 20 mL of ethyl acetate were added and the organic phase was extracted 5 times with 15 mL of 10% NaCl solution. The organic phase was dried over sodium sulfate and precipitated in 120 mL of *n*-hexane. The precipitate was filtered, washed with n-hexane and dried under vacuum. The product was obtained as an off-white solid in a yield of 89% (2.66 g, 8.90 mmol).

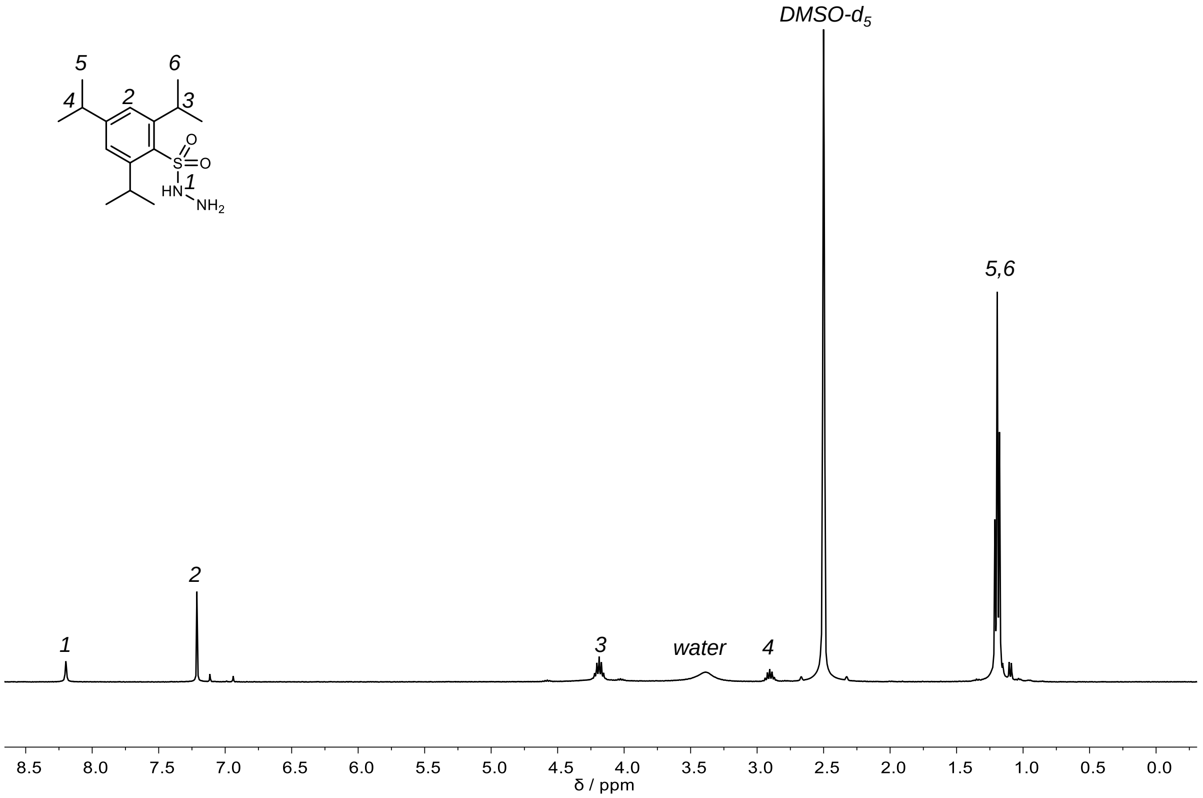

^{1}H NMR (400 MHz, DMSO-d_6): δ/ppm = 8.20 (s, 1H), 7.21 (s, 2H), 4.19 (hept, J = 6.8 Hz, 2H), 2.91 (hept, J = 6.9 Hz, 1H), 1.28 – 1.13 (m, 19H).

Note: Due to the thermal instability of the compound in solution, the unassigned signals correspond to the decomposition products (presumably the corresponding sulfinic acid). No ^{13}C NMR spectra were recorded for this reason.
Furthermore, the NH_2 signal is not visible in the ^{1}H NMR spectrum, presumably due to high water content.

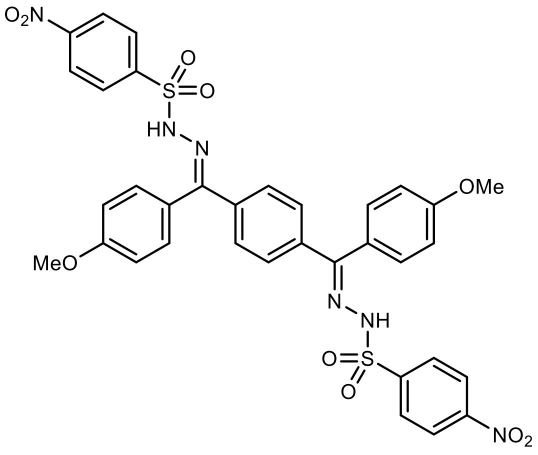

*Attempted synthesis of 4,4'-dimethoxyterephthalophenone bis-(*p*-nosylhydrazone)* (**M1'**)

In a glass pressure vial, diketone **K1** (866 mg, 2.50 mmol, 1.00 equiv.) was suspended in 10 mL of ethanol and *p*-nosyl hydrazide (1.63 g, 7.50 mmol, 3.00 equiv.) along with *p*-toluenesulfonic acid monohydrate (47.6 mg, 0.250 mmol, 0.10 equiv.) was added. The vial was closed and stirred at 80 °C for 8 hours. Afterwards, the mixture was cooled to room temperature, the precipitate was filtered, washed with cold ethanol and dried under vacuum. The ^{1}H-NMR of the isolated product indicated that only 38% of **K1** were converted into the desired product **M1'**.

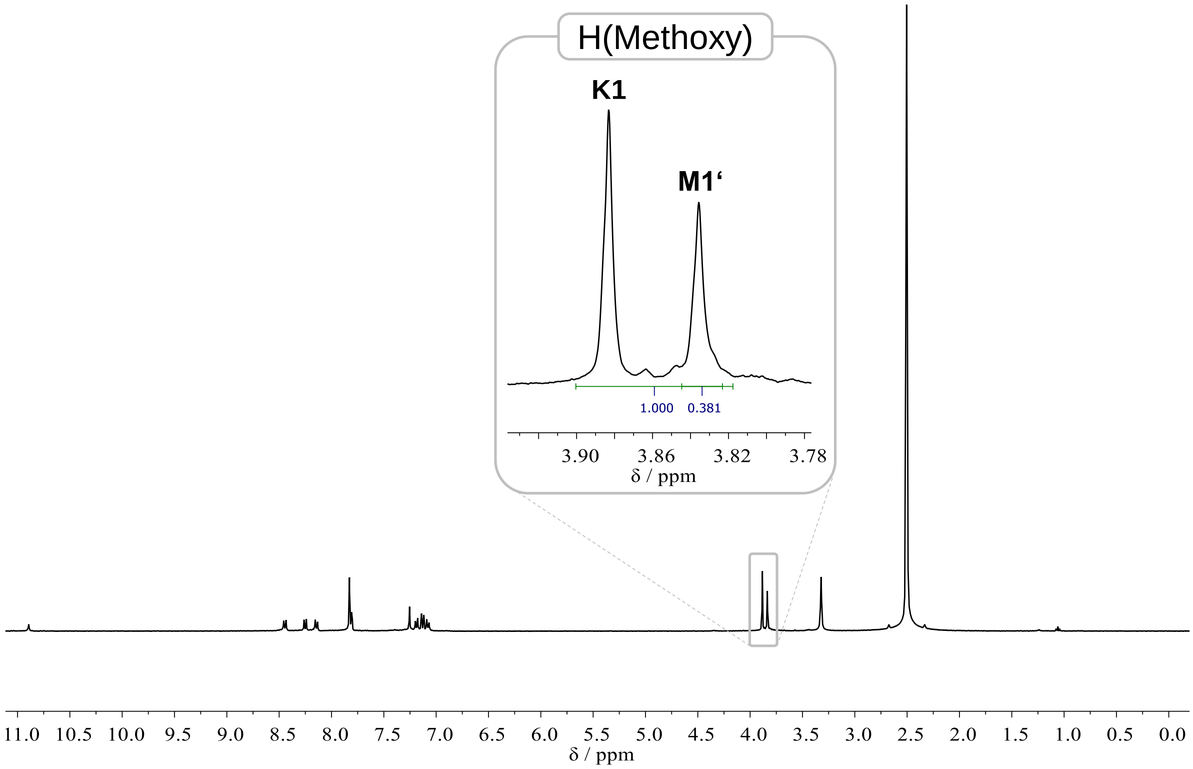

3.4 Screening Results

3.4.1 Polymerization Screenings

The results of all screening experiments conducted in the context of the optimization conditions of the polymerization of **M1** to **P1** are listed in Table S1. The screening was conducted on a scale of 0.125 mmol **M1**. The screening reactions were worked up according to the general procedure (described in section 3.2) and analyzed using size exclusion chromatography. For entries 1 and 2, additional differential scanning calorimetry measurements were conducted (Figure S1). The presence of sulfur in the polymer obtained prior to the workup optimization is apparent via the endothermic melting peak observed around 120 °C, which is absent in the polymers obtained after workup optimization.

***Table S1**: Screening Results for the Polymerization of **M1** to **P1**. Reactions were conducted in DMSO (0.25 M) using 2.40 equiv. of potassium carbonate and 0.50 equiv. of elemental sulfur.*

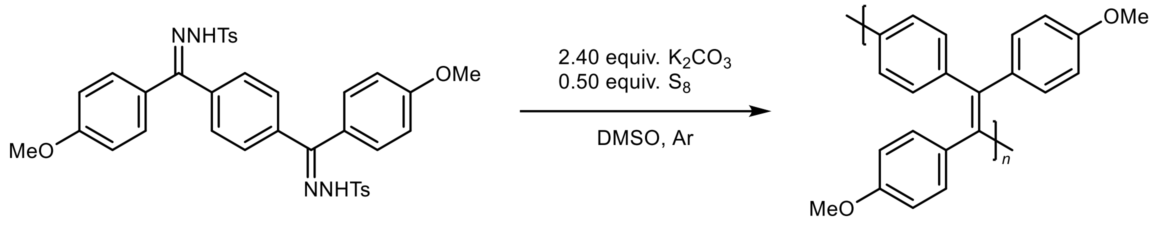

M1 **P1**

Entry	Temp. / °C	Yield	*Atmosphere*	M_n / g mol^{-1}	M_w / g mol^{-1}	*Đ*	Comments
1	100	>100%	Air	5960	12400	2.08	No optimized workup
2	100	46%	Air	6250	12300	1.93	
3	120	28%	Air	5270	9500	1.80	
4	140	48%	Air	4130	11200	2.70	
5	100	40%	Inert	7800	16600	2.12	
6	120	42%	Inert	7500	17500	2.33	
7	90	60%	Inert	8800	20000	2.28	
8	80	64%	Inert	10800	24800	2.30	
9	**70**	**76%**	Inert	**16400**	**34200**	**2.11**	
10	80	72%	Inert	10700	24100	2.25	c = 0.5 M
11	80	74%	Inert	10400	21300	2.05	c = 0.125 M

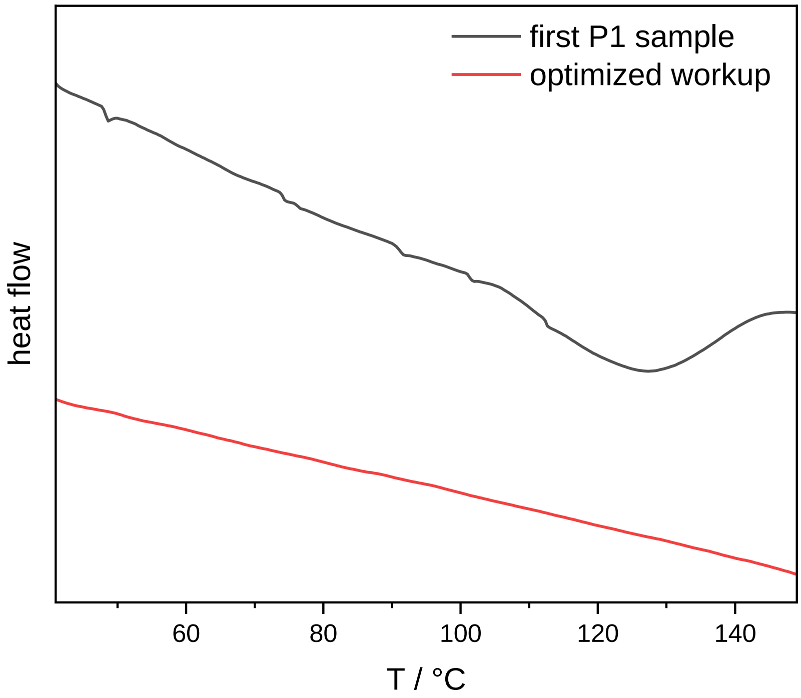

Figure S1: DSC measurements of the first obtained sample of ***P1*** *(Table S1, entry 1, black curve) and a* ***P1*** *sample obtained after workup optimization (Table S1, entry 2, red curve). The endothermic peak at ~120 °C for the pre-optimized* ***P1*** *sample indicates the presence of residual sulfur in the polymer.*

3.4.2 Screening of monofunctional sulfonylhydrazone coupling

The results of all screening reactions that were conducted using the monofunctional sulfonylhydrazones **2** and **3** are listed in Table S2. The screening reactions were conducted in DMSO-d_6 on a scale of 0.25 mmol hydrazone. The hydrazone conversion was monitored using ^{1}H-NMR spectroscopy using the methoxy signals of **2**/**3**. Full conversion was assigned if no more substrate signal was discernible in the spectra.

Table 2: Screening results using the monofunctional sulfonylhydrazones ***X2*** *and* ***X3****. Reactions were conducted in a concentration of 0.25 M using 2.40 equiv. of base and 0.50 equiv. of elemental sulfur.*

Entry	Substrate	T / °C	Atm.	Solvent	Base	t / h	Conversion
1	**2**	100	Air	DMSO	K_2CO_3	0.5	Full
2	**2**	90	Air	DMSO	K_2CO_3	0.5	Full
3	**2**	80	Air	DMSO	K_2CO_3	1.5	93
						3	99
4	**2**	80	Argon	DMSO	K_2CO_3	0.5	90
						1.5	99
						3	Full
5	**2**	70	Argon	DMSO	K_2CO_3	2	64
						4	85
						16	Full
6	**2**	60	Argon	DMSO	K_2CO_3	4	45
						23	94
						72	98
7	**3**	70	Argon	DMSO	K_2CO_3	0,5	36
						1	57
						2	92
						4	93
						16	Full
8	**3**	60	Argon	DMSO	K_2CO_3	4	51
						23	99
						48	Full
9	**2**	80	Argon	Ethylene glycol	K_2CO_3	0,5	36
10	**2**	80	Argon	Triglyme	K_2CO_3	0,5	0
11	**2**	80	Argon	MeCN	K_2CO_3	0,5	0
12	**2**	80	Argon	DMC	K_2CO_3	0,5	0
13	**2**	80	Argon	2-methyl-THF	K_2CO_3	0,5	0
14	**3**	70	Argon	DMSO	Cs_2CO_3	1	54
15	**3**	70	Argon	DMSO	K_3PO_4	1	30
16	**3**	70	Argon	DMSO	KO^tBu	1	51
17	**3**	70	Argon	DMSO	NaOMe	1	35

4. Supplementary References

[1] W. Zhao, E. M. Carreira, *Org. Lett.* **2006**, *8*, 99–102.
[2] P. Conen, R. Nickisch, M. A. R. Meier, *Commun. Chem.* **2023**, *6*, 255.
[3] C. G. Sanz, K. A. Dias, R. P. Bacil, R. A. M. Serafim, L. H. Andrade, E. I. Ferreira, S. H. P. Serrano, *Electrochim. Acta* **2021**, *368*, 137582.
[4] A. G. Myers, B. Zheng, M. Movassaghi, *J. Org. Chem.* **1997**, *62*, 7507–7507.
